# Bayesian model of tilling wheat confronting climatic and sustainability challenges

**DOI:** 10.3389/frai.2024.1402098

**Published:** 2024-08-27

**Authors:** Qaisar Ali

**Affiliations:** Department of Sustainable Land Management, SAPD, The School of Agriculture, Policy, and Development, University of Reading, Reading, United Kingdom

**Keywords:** Bayesian model, climate change, sustainable challenges, tillage preferences, NFM strategies, synthetic datasets, DSSAT simulations, GHG-CO_2_ emissions

## Abstract

Conventional farming poses threats to sustainable agriculture in growing food demands and increasing flooding risks. This research introduces a Bayesian Belief Network (BBN) to address these concerns. The model explores tillage adaptation for flood management in soils with varying organic carbon (OC) contents for winter wheat production. Three real soils, emphasizing texture and soil water properties, were sourced from the NETMAP soilscape of the Pang catchment area in Berkshire, United Kingdom. Modified with OC content at four levels (1, 3, 5, 7%), they were modeled alongside relevant variables in a BBN. The Decision Support System for Agrotechnology Transfer (DSSAT) simulated datasets across 48 cropping seasons to parameterize the BBN. The study compared tillage effects on wheat yield, surface runoff, and GHG-CO_2_ emissions, categorizing model parameters (from lower to higher bands) based on statistical data distribution. Results revealed that NT outperformed CT in the highest parametric category, comparing probabilistic estimates with reduced GHG-CO_2_ emissions from “7.34 to 7.31%” and cumulative runoff from “8.52 to 8.50%,” while yield increased from “7.46 to 7.56%.” Conversely, CT exhibited increased emissions from “7.34 to 7.36%” and cumulative runoff from “8.52 to 8.55%,” along with reduced yield from “7.46 to 7.35%.” The BBN model effectively captured uncertainties, offering posterior probability distributions reflecting conditional relationships across variables and offered decision choice for NT favoring soil carbon stocks in winter wheat (highest among soils “NT.OC-7%PDPG8,” e.g., 286,634 kg/ha) over CT (lowest in “CT.OC-3.9%PDPG8,” e.g., 5,894 kg/ha). On average, NT released minimum GHG- CO_2_ emissions to “3,985 kgCO_2_eqv/ha,” while CT emitted “7,415 kgCO_2_eqv/ha.” Conversely, NT emitted “8,747 kgCO_2_eqv/ha” for maximum emissions, while CT emitted “15,356 kgCO_2_eqv/ha.” NT resulted in lower surface runoff against CT in all soils and limits runoff generations naturally for flood alleviation with the potential for customized improvement. The study recommends the model for extensive assessments of various spatiotemporal conditions. The research findings align with sustainable development goals, e.g., SDG12 and SDG13 for responsible production and climate actions, respectively, as defined by the Agriculture and Food Organization of the United Nations.

## 1 Introduction

Climate change and sustainability challenges have appeared as substantial threats affecting numerous aspects of life, including agriculture (Whitmee et al., 2015; Cramer et al., 2018). Climatic factors are essential as unpredictable weather events directly influence farming practices. Excessive rainfall or drought conditions can intensely affect farming actions and outcomes. For instance, prolonged drought leads to water shortage, crop yield limitations, and soil erosion. Contrarily, heavy rainfalls lead to flash flooding, delay crop planting and harvesting, and impact crop yields and losses. Excessive moisture levels affect crop quality and attract insect pest attacks and disease infestations. Similarly, extreme temperatures influence key plant development stages, affecting crop growth and yield (Nnadi et al., 2013; Rial-Lovera et al., 2017). Human-induced activities are recognized as significant contributors to climate change, often resulting from unsustainable environmental actions. These actions include deforestation, land-use practices, greenhouse gas emissions, industrial activities, burning fossil fuels, and waste management (Retnowati et al., 2014). Consequently, making the right decisions and implementing appropriate methods have become pertinent, as the ramifications will have far-reaching implications for future generations (Adger et al., 2005). The solution relies on sustainability-based actions (Gibson, 2001). Identifying and adopting the right choices among alternatives can lead to embracing sustainable solutions. The potential exists to consider one decision among others to serve the wider ambit (Walker, 2014).

The primary factors in farming are local weather conditions, soil types, and soil organic carbon levels (Mkonda and He, 2023). There is little room and limited choices for these factors to play around beyond certain points. For instance, geographical & seasonal weather conditions and extreme events can limit farming choices. However, local soilscapes exhibiting diverse health conditions should prioritize enhancing and sustaining their vigor to withstand climatic pressures and ensure sustainable yield. Hence, this study investigated soilscapes to extract real soil attributes as fundamental components (Field, 2012; Vermeulen et al., 2012). Soil organic carbon (SOC) content levels are the main indicator of the soil health in the local soils for performing farming functions, which can help build good soil structure, delivering effective crop production in return (Ryan et al., 2009). SOC serves as a reservoir of essential nutrients and is pivotal in supporting nutrient availability through ascertaining soil nutrient cycling for nitrogen, phosphorus, and sulfur. Soils with higher SOC levels improve soil fertility and reduce reliance on external inputs such as synthetic fertilizers. Hence, the SOC levels have a compelling influence on crop productivity. Higher SOC levels enhance soil water-holding capacity, which helps retain moisture and resist drought stress by helping crops withstand water scarcity periods and maintain moisture levels essential for crop growth and development (Carter, 2002). Understanding the appropriate carbon-soil organic matter (C-SOM) level is challenging, which must focus on achieving environment friendly soil carbon stocks and sustainable productivity. Testing different SOC levels in real-life situations may face spatiotemporal limitations. It can make the exploration costly and ineffective, requiring resource-intensive, prolonged field experimentations (Sierra et al., 2015; Searchinger et al., 2018; Murindangabo et al., 2023).

Mathematical modeling techniques are applied to perform this task most efficiently because the scientific approach of using modeling tools can offer scenario-based choices among alternatives to evaluate the impact of one decision over the others (Collins et al., 2013). This approach is efficient and flexible in making necessary adaptations according to the situation. Among several modeling techniques, the Bayesian modeling approach provides a comprehensive and flexible framework which can represent complex relations and diverse systems, such as the influence of Climate change and sustainable production challenges in current farming practices. Heavy rainfall events can turn into flash flooding, and extreme temperature or drought situations can cause water stress in crop growth and development. Adapting appropriate farming practices, such as tillage preference, maintaining SOC levels, and limiting GHG emissions, becomes extremely important. Bayesian networks can capture uncertainties among complicated interactions between climatology, agronomy, ecology, ecosystem dynamics and socio-economic factors to enhance resilience and sustainability. Moreover, BBN can integrate multi-domain knowledge to support decision-making choosing among alternatives (Collins et al., 2013; Stockmann et al., 2013; Parton et al., 2020; Heidari et al., 2021).

The Bayesian Belief Network (BBN) modeling technique’s rationale is its ability to handle uncertainty, integrate prior knowledge, and provide probabilistic inference. Bayesian methods offer a flexible framework for analyzing complex systems, allowing for integrating various sources of information and quantifying uncertainty in model predictions. Additionally, the BBN facilitates updating beliefs as new data becomes available, enabling iterative refinement of models and decision-making processes. Bayesian network modeling is one approach that offers the flexibility to choose several relevant factors as variables of interest to include in building a BBN model structure (Natcvetova, 2021). The BBN model can represent common and scientific interest phenomena with underlying conditional associations. This feature enables the BBN model to capture the hidden uncertainty among the complex interactions (Dal Ferro et al., 2018). These interfaces offer conditional probabilities to interrelate based on the intensity of dependencies backed by causality or conditional relationships among interacting variables. This phenomenon applies under the parent and child relationship, where a parent is independent, and a child is dependent on a parent. A variable is called a node connected to another node where the parent node is the causal or independent variable, and the child node is the dependent or effect node. A parent node connects its child node through a link arrow directed from the parent to the child node to avoid having a complete cycle. This structure develops into a network model structure, which means a directed network graph without any cycle and is also known as a directed acyclic graph (DAG) (Donald, 2011; Gopal et al., 2012).

Determining conditional relationships is also very important in this modeling process. Several approaches are used in the BBN modeling frameworks to include such variables as part of a network structure. Manual, data-driven, and hybrid approaches are known methods for constructing a BBN model using expert elicitation, data-based learning, and a combination of the earlier two approaches. There are different pros and cons for each approach. Most have limitations in reaching the sources due to Spatio-temporal factors (Neil et al., 2000; Heckerman, 2008; Koski and Noble, 2012). However, modelers or model designers can opt for one or the other approaches to identify the vital variables and their pertinent interactions for building BBN models. For instance, various modeling tools use efficient algorithms representing certain phenomena or scientific systems following ascertained relations among various variables. This study pursued established interactions for developing a new BBN model and found an opportunity to evaluate the variables’ performance with new data inputs. Parts of these could be helpful to utilize where access to limited datasets becomes a hindrance (De Oliveira and Gaudio, 2000; Pearl, 2011). The technique is also helpful where datasets are highly demanding to explore long-term impacts for predictive inferences and access to real-time empirical data is limited for other reasons. These tools can help produce synthetic datasets by executing simulations based on similar experimental setups induced with available minimum dataset applications (Drury et al., 2017; Groth et al., 2019). The hierarchical model with a process-based framework may facilitate this goal.

Process Based Models (PBMs) have been used for predicting yield and environmental regulations of plant physiological processes, which increased the use of data acquisition through sensors and technologies (Sands et al., 2000). This trend amplified the use of machine learning (ML) tools, which became popular with time. Several techniques are introduced combining different tools for attaining high prediction accuracy, such as PBM and ML were combined to develop knowledge- and data-driven. This approach presented a combination of simulation models with data science in agriculture, focusing on genetic and physiological processes involved in food production, climate change mitigation and sustainability (Hailegnaw et al., 2024). A team of researchers also introduced a comparable approach for integrating machine learning and empirical evapotranspiration modeling to explore implications for agricultural water management using the Decision Support System for Agrotechnological Transfer (DSSAT). They compared three empirical models (Hargreaves Samani – HS, Priestly Taylor – PT, and Turc - TU) with three machine learning models (Multiple linear regression – LR, Random Forest – RF, and Artificial Neural Network – NN). They found that machine-learning models outperformed the empirical models. They also informed that site specific model calibration is extremely significant for higher predictive accuracy (Korb, 2009). The potential to handle target variables and crucial processes and reflect their responses in a mechanistic model (DSSAT) is an extremely important point.

The rationale for using DSSAT to generate simulated datasets is its reputation as a robust and widely used agricultural simulation model. DSSAT integrates various modules such as crop growth, soil processes, and climate data to simulate agricultural systems precisely. Using DSSAT, researchers can simulate diverse agricultural settings under different conditions, providing valuable insights into crop performance, soil dynamics, and environmental impacts (Jones et al., 2003). This approach allows for confidence in simulated data acquisition when access to real-world data is limited. A team of scientists used the soil management data module of the DSSAT model for analyzing various types of soils with contents for their crop suitability and prediction yields by observing various input parameters such as soil, weather, rainfall, etc. (Zhang et al., 2023). Hence, the integration of synthetic datasets is targeted through executing a process-based model for data simulations using the DSSAT tool. And prototype model in the DSSAT requires a minimum level of empirical data requirement to calibrate and execute the model, which delivers the output responses of the pertinent variables aimed at the BBN model for this study. A research scientist described that the BBN models are the new generation of probabilistic systems which capture uncertainty by modeling the physical, biological and social systems (Yu et al., 2004; Cheema and Singh, 2021). Combining DSSAT with BBNs allows researchers to leverage the detailed process-based simulations provided by DSSAT while incorporating uncertainties and prior knowledge into the modeling framework using BBNs. This integration enables more robust decision-making in agricultural systems by providing probabilistic assessments of crop performance, soil dynamics, and environmental impacts while accounting for uncertainties inherent in the modeling process (Finley et al., 2011).

In rainfed agricultural systems, farming has several other associated challenges, not limited to flooding risk in the context of climate change and sustainability (Shiferaw et al., 2009; Lashford et al., 2022). Member states of the Food and Agriculture Organization (FAO) of the United Nations Organization have the understanding to achieve their targets for sustainable development goals (SDGs). In farming, SDG12 for responsible production and SDG13 for climate action measures are the most relevant targets to focus on achieving sustainability in targeted areas (Louman et al., 2019; Hales and Birdthistle, 2023; Reijneveld et al., 2023). These aspects have become extremely important in farming to consider the right choices and activities which could not endanger the environment and must not result in detrimental outcomes for others. Responsible production approaches, in combination with climate action measures, could serve this purpose (Beerling et al., 2018). For instance, whether land tilling methods (conventional or no-tillage) reduce surface runoff to facilitate flood alleviation during wet seasons without compromising the main purpose of attaining sustainable crop yield?

Tilling systems in winter wheat production are the most significant area of attention in the context of climate change and sustainability in this research study. However, tilling systems can substantially influence soil structural changes, health, carbon sequestration, moisture conservation, climate-related risks and sustainable land management in dry summers and wet winters (Hermle et al., 2008). In Berkshire, United Kingdom, farming has local challenges of flash flooding and groundwater table issues. In the winter of 2000/2001, the Pang and Lambourn catchments in Berkshire suffered from groundwater flooding, and the water table reached the land surface and produced long-duration surface flows (Hughes et al., 2011). Catchment-based, natural flood management (NFM) approaches are vital for formulating solutions around other farming objectives. Adaptation of appropriate tillage systems between no-till (NT) or conventional tillage (CT) for winter wheat can deliver in the face of Climate change and sustainability challenges. NT represents limiting the interface between soil and farm machinery or tilling implements. This practice is also known as zero tillage, conservational or non-inversion tillage. At the same time, CT presents conventional methods of tilling soils with frequent, intense tilling with regular interfaces between soils and farm machinery/ tilling implements for agronomic practices for crop establishment for production. This practice is also known as traditional inversion tillage. This study hypothesized that tillage preference could be an NFM strategy and reduce GHG-CO_2_ emissions to deliver sustainable crop yield. However, local soilscapes, which have diverse soil compositions and proportions of sand, silt, gravel, bulk density, and SOC content levels, remained the key focus of the study (Broadmeadow et al., 2023).

Based on the above background, this study focuses on achieving the following.

- Comparing tilling preference as an NFM strategy in winter wheat production for climate resilience.
- Quantifying the uncertainty among variables with complex interactions using long-term simulations.
- Evaluating tilling impacts for wheat production, surface runoff and greenhouse gas carbon dioxide (GHG-CO_2_) emissions against soil carbon variability.

## 2 Methods

### 2.1 Case study and data acquisition

The study site is associated with the River Pang adjacent to the Kennet Catchment. Site selection criteria are mainly linked with a project partner, e.g., Landwise NFM Project. They held the license for data acquisition for the catchment. This research was a part of modeling work packages for exploring NFM strategies for local farm production. Moreover, the site is near the University of Reading, Berkshire, United Kingdom. The University of Reading Weather Observatory provided the weather data used in this study. Access to resources such as data acquisition remained the key consideration for catchment selection for this research. The Pang River flows through the Berkshire Downs to join the River Thames at Pangbourne. The ground water table is the main fed water, and a little water from tributaries drains the cover of tertiary clay and sand in the lower catchment. The Pang has some challenges due to physical modifications, water quality, low flow, flooding and invasive species. Physical modifications are not limited to building structures such as culverts, bridges, tracks, infrastructure, etc., next to watercourses and water bodies. They lead to the loss of natural processes hampering the water environment (Pang River, 2020).

The Environment Agency monitors water bodies and catchment areas for their ecological, physio-chemical quality elements, hydro morphological supporting elements, chemical, priority substances and other pollutants. They classified the ecological status of the Pang as moderate and the water body type as a river, and the Pang water body administered that. The length of the river is 36.57 km^2,^ and the catchment area is 170.531 km^2^ (Pang Water Body, 2024). There is no declared protected status. However, small sites of specific scientific interest are in the wider catchment, including Sulham, Tidmarsh Woods, Meadows, Coombe Wood, and Frilsham. The Pang Valley has a history of fluvial and groundwater flooding. The risk of flooding from surface water extent is about 0.1 percent annually (About the Pang, 2019). Local farming revolves around farming objectives and their interactions with businesses and lifestyles. The main land cover plus crops include grasses, wheat, oilseed rape, barley, maize, potato, beans, potatoes, etc. Winter wheat is a major cereal crop widely cultivated in arable farming systems in the catchment (Pearce, 1952; Perkin and Rehman, 1994).

This study utilized the Decision Support System for Agrotechnology Transfer (DSSAT), a software application that delivers dynamic crop growth simulation models for different crops. This tool evolved with multiple functions, incorporating weather, soil, crop management, and sample datasets for several crop models. The crop simulation models within DSSAT simulate crop development, growth, and yield based on the interaction of soil–plant-atmosphere dynamics (Jones et al., 2003). Employing three core modules, namely the Sbuild-module (for soil data file input), Weatherman-module (for climate data file input), and the Xbuild-module (for crop management data input), this tool facilitated wide-ranging crop growth modeling research (Decision Support System for Agrotechnology Transfer, n.d.). Hence, this research explored the DSSAT tool, found the most appropriate prototype experimental model for winter wheat and incorporated the available datasets (such as soils, winter wheat crop management, and climatic weather information) from the catchment area.

This research applied sample data features from the local soilscape, which describe the spatial variation and diversity of soils in a landscape. This data includes a range of soil types, textural composition, physical properties, and key features in a particular area. Various soil mapping and classification techniques delineate soilscapes and characterize spatial distribution and variability across landscapes (Farewell et al., 2011). Supplementary Table S1 shows the soilscapes explored for the Pang catchment. Soilscapes range from medium soils, deep clays/ silty, chalk and limestone soils with varying organic carbon contents. Important catchment features are highlighted in the map, shown in Supplementary Figure S1. This study used key features of data samples, which include soil features from soilscape, weather indices from climate, and winter wheat crop phenology from a prototype experiment. Data acquisition for three soils from local soilscape and weather indices of the Pang catchment area were accomplished through the relevant sources (NETMAP soilscape data) by the NFM Landwise Project and the University of Reading, United Kingdom Atmospheric Observatory, respectively. Three soilscape PDPG6, PDPG7 and PDPG8, containing key soil features, characteristics and spatial distribution, are summarized in Supplementary Table S1. These are not the entire soilscapes of the catchment but represent a considerable land in the Pang catchment area. This modeling research used detailed variability of real soils for given zones depth/ layers, soil texture, composition, SOC, bulk density and saturated hydraulic conductivity, lower limit, drained upper limit and saturated water contents are highlighted in the brown colored section of the Supplementary Table S2 for PDPG6, Supplementary Table S3 for PDPG7, and Supplementary Table S4 PDPG8, respectively. The “Weather indices” data file is available in the Supplementary material; the soilscape information is described in Supplementary Table S1, with experimental details in Supplementary Table S5 (Jamagne and King, 2002; Rameshwaram et al., 2023; Crop Cover Data Source, 2024; Geology Data Source, 2024; Land Cover Data Source, 2024; Soilscapes Data Source, 2024; Weather Data Source, 2024).

The study incorporated soils as independent variables, utilizing real soil samples with original organic carbon content from various soilscapes. Classification and characterization details are presented in Supplementary Table S1; Supplementary Figure S1. The study applied the “RB209” soil classification approach for this project. Other independent variables include weather data of seasonal rainfall (mm) and temperature (C^°^) from the Reading Atmospheric Observatory (Weather Data Source, 2024), as this research compared no-tillage versus conventional tillage practices in winter wheat crop production with tillage preference as treatment under a frequentist approach (Supplementary material S2) which represented as the main study input variable (independent variable) under the BBN modeling approach.

Four model soils were synthesized based on the three real soils mentioned above, with varying organic carbon contents in topsoil (1, 3, 5, 7%) (Table 1). The variations in organic carbon content within the modeled soils were adjusted in proportion to their existing levels in the real soil relative to the mineral particles. These variations were implemented using the Sbuild-module of the Decision Support System for Agrotechnology Transfer (DSSAT) version 4.8, with each layer adjusted accordingly (Decision Support System for Agrotechnology Transfer, n.d.). Fifteen soil profiles, representing each real soil from a soilscape along with its four modeled counterparts, were generated using the Sbuild-module of DSSAT. Refer to Supplementary Table S1 and Supplementary Tables S2–S4 for more information. Detailed mechanisms for synthesizing modeled soils are described as “Keynotes” under Supplementary Table S4. The Sbuild-module of the DSSAT tool applied the below regression equation on input data of soil texture to estimate the carbon in the stable organic matter based on the relationship developed by po Adiku (Porter et al., 2010; Prout et al., 2021). The Sbuild shell of the DSSAT tool calculated the bulk density of each layer of modeled soil.

**Table 1:** A schematic plan comparing no-till and conventional tillage methods was designed for experimenting three real soils from the soilscape, each synthesized into four modeled soils with top layer organic carbon content levels of 1, 3, 5, and 7% using the S-module of the DSSAT tool.

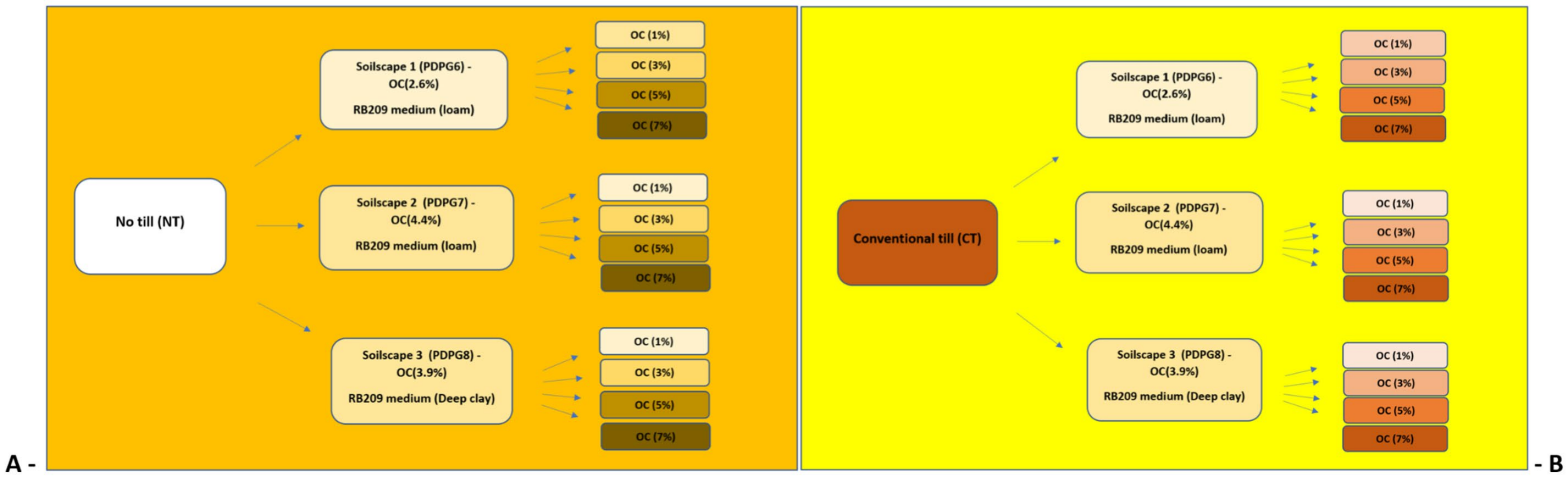

The DSSAT4.5 Version 1 describes the regression equation as under.

Stable C = 0.15 x (Clay + Silt) + 0.69 where.

Stable C = stable organic C in g/kg,

Clay = soil clay content in %,

Silt = soil silt content in %.

This study applied a given prototype experiment (RORO7401) in the DSSAT tool for implying phenological data for winter wheat crop production for N210 kg/ha treatment, matching this as similar conditions to that practiced in the study catchment area as shown in experiment details in Supplementary Table S5 (Porter et al., 2010). Moreover, this experiment was adapted to investigate having the same crop of winter wheat, reflecting comparable seasonal weather patterns, matching geographical climate, agro-ecological environment and landscape features that existed in temperate weathers practiced under rainfed agriculture in the United Kingdom. The Xbuild module is a crop management data editing program in the DSSAT. Based on the study objectives, the Xbuild module of the DSSAT tool is used to make necessary updates in the management tab for tillage applications such as tillage implements, frequency and depth, etc. Specific actions administered in the Xbuild module are depicted in the extended Supplementary Table S5. The role of the Xbuild module facilitated in setting up of diverse tillage practices for this research exploration and enabled to establishment of two diverse experiments, e.g., one for conventional tillage (CT) and the other for no-till (NT). This approach helped to have comparative responses of interacting variables through segregated long-term simulations. All other wheat crop phenological data remained unchanged in the DSSAT except for having local weather (time series) data for the respective experiment (by introducing a new weather station as “WUOR” in the weatherman module of the DSSAT tool for 48 years from 1974 to 2022) and local soilscape data file (by introducing “RD.SOL” in Sbuild-module for 15 soil profiles). Supplementary Tables S2–S4 (for soils) and Supplementary Table S5 (for experimental details) contain the necessary details pertaining to parametric attributes in the Sbuild and Xbuild modules, respectively (Table 1).

Three soils (PDPG6, PDPG7, and PDPG8) and their attributes from the NETMAP soilscapes data were acquired from the Project partner. These soils were labeled to correspond with their original identification within the soilscape. This study uses soilscape terminology interchangeably with soils to prevent technical confusion. The variable labeled ‘soilscape’ in the BBN model represents soils in this research. The soil organic carbon levels in real soils of the local soilscape are represented by PDPG6 (OC-2.6%), PDPG7 (OC-4.4%), and PDPG8 (OC-3.9%). Thirty soils, 15 for No-till (NT) and 15 for Conventional till (CT), were individually processed to simulate 48 years (1975–2022) using the DSSAT.

The Cropping System Model (CSM) was employed within DSSAT v4.8 and contains various crop models to have simulated data for longer periods (Jones et al., 2003; Li et al., 2015). The crop models use the CERES models for wheat, and various controls & management scenarios are employed within the shell to simulate crop growth. Hence, 15 soils each for No-till and Conventional till (CT) executed in the CSM of DSSAT for having 1,440 simulations over 48 years from 1974 to 2022 and a summary of experiments is described in Table 2. The rationale for conducting simulations over an extended period is not only linked with the evaluation of historical data for improved forecasting of the variable of interest but also encompasses the provision for accessing the model with training and testing datasets. This method helped to find the responses of soils demonstrating local soilscapes containing varied carbon contents to deliver influence on wheat yield, surface runoff and GHG-CO_2_ emissions through related variables over 48 years in No-till (NT) and conventional till (CT) management applications using the Cropping System Model (CSM) of the DSSAT (Zhao et al., 2024).

**Table 2:** The DSSAT model was used to generate synthetic datasets for 30 individual soils, e.g., one real (in orange) with four modeled (pink) soils from each of 3 real soils from local soilscapes, executing distinctive sets of simulations each over 48 years from 1974 to 2022.

	No-till (NT)					Conventional till (CT)				
PDPG6	Experiment 1	Experiment 2	Experiment 3	Experiment 4	Experiment 5	Experiment 6	Experiment 7	Experiment 8	Experiment 9	Experiment 10
	NT.OC-2.6%.PDPG6	NT.OC-1%.PDPG6	NT.OC-3%PDPG6	NT.OC-5%PDPG6	NT.OC-7%PDPG6	CT.OC-2.6%PDPG6	CT.OC-1%PDPG6	CT.OC-3%PDPG6	CT.OC-5%PDPG6	CT.OC-7%PDPG6
PDPG7	Experiment 11	Experiment 12	Experiment 13	Experiment 14	Experiment 15	Experiment 16	Experiment 17	Experiment 18	Experiment 19	Experiment 20
	NT.OC-4.4%.PDPG6	NT.OC-1%.PDPG6	NT.OC-3%PDPG6	NT.OC-5%PDPG6	NT.OC-7%PDPG6	CT.OC-4.4%PDPG6	CT.OC-1%PDPG6	CT.OC-3%PDPG6	CT.OC-5%PDPG6	CT.OC-7%PDPG6
PDPG8	Experiment 21	Experiment 22	Experiment 23	Experiment 24	Experiment 25	Experiment 26	Experiment 27	Experiment 28	Experiment 29	Experiment 30
	NT.OC-3.9%.PDPG6	NT.OC-1%.PDPG6	NT.OC-3%PDPG6	NT.OC-5%PDPG6	NT.OC-7%PDPG6	CT.OC-3.9%PDPG6	CT.OC-1%PDPG6	CT.OC-3%PDPG6	CT.OC-5%PDPG6	CT.OC-7%PDPG6

NT means no-till; CT means conventional tillage; OC means organic carbon level; and PDPG number represents soil ID to soilscape.

### 2.2 Synthesis of Bayesian network model structure

The BBN model structure was synthesized by retrieving evidence reported in the published literature and practical applications available and employed in comparable software. Summary of modules for variables interactions applied in the DSSAT and the list of variables with their relationships and evidence from the literature for BBN model structure are highlighted in Supplementary Tables S6, S7. The model structure for variable relationships was also confirmed through the source manuals available with the software, e.g., the DSSAT and AquaCrop. Key considerations revolved around ascertaining and validating the model assumptions as defined and identified in the relevant sections of this manuscript (Waffa and Benoit, 2015).

**Figure 1:**
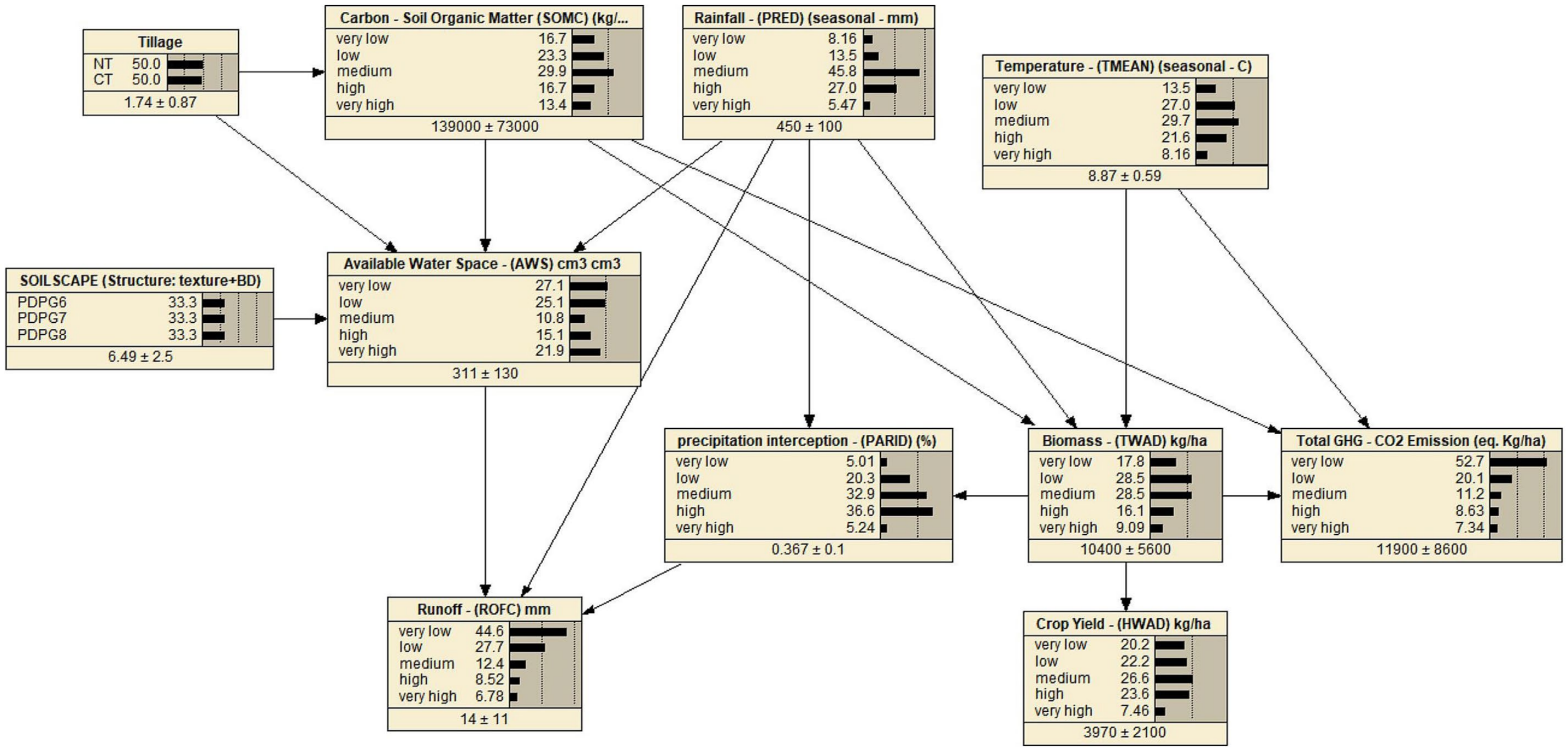
The BBN model shows 11 nodes (variables), 17 links (relationships) and their belief based on parametric learning through simulated datasets (75%) generated in the CERES-Wheat model of the DSSAT.

This study used the Netica software interface to build a graphical network structure and represented variables as nodes and their relationships as a link or arcs representing their connection. The identified conditional (ideally causal) relationships evident through the published literature determined the direction of the link or arc (Supplementary Table S7). A single relationship between two variables helped develop a simple BBN model structure foundation. This rationale is followed for every single relationship to build a Directed Acyclic Graph (DAG), which delivers a model structure having all interrelated variables connected in a network, as shown in Figure 1 (Ni et al., 2011). Additionally, the study proved the structural morphology of the BBN model, analyzed by the Netica software, which demonstrated key interactions and is shown in Supplementary Table S9. These interactions depicted their probabilistic estimates and performance accuracy, delivering the desired model outcomes. The model was evaluated through key performance indicators outlined for model outputs in the following sections.

This study selected the output (dependent) variables based on the study objectives for assessing tillage preferences toward winter wheat production, as tillage practices are practiced to prepare land for crop establishment through farm management to achieve crop yield. Hence, crop yield suits a major output (dependent) variable (Jug et al., 2011). Surface runoff is another vital variable that can measure flood risk reduction dependent upon tilling choices to facilitate or resist the surface water flow during cropping seasons. For instance, decreased runoff facilitates flood alleviation while tilling wheat if no-till or reduced tilling preference is opted. This approach will cover the land through vegetation and limit the interface between soil cover and farm machinery. Hence, surface runoff serves as another vital output (dependent) variable. Wheat tilling on bare land or cover (such as crop residue or mulches on land) can create a difference in resisting surface runoff during wet winters (DeLaune and Sij, 2012). Last but not least, greenhouse gas emissions for carbon dioxide (GHG-CO_2_) are another major output (dependent) variable because tillage preferences are linked with the disturbance of soil aggregates, impacting the decomposition rate of soil organic matter and carbon sequestration, etc. Hence, GHG-CO_2_ emissions were considered a vital output (dependent) variable based on tillage preferences (Galic et al., 2019).

To quantify output (dependent) variables in response to input (independent) variables of rainfall, temperature, soils from soilscapes, and tillage systems, this research explored the output variables response accessible through the DSSAT cropping system model (CSM). This study included the most applicable variables with conditional or causal influence among conditional interactions. For instance, the abovementioned parameters are focused as input variables in the DSSAT shell. The study investigated output variables of biomass, yield, precipitation interception, available water space, runoff, carbon-soil organic matter, and GHG-CO_2_ emissions. The investigations also explored the literature to confirm such key relationships. For instance, crop biomass accumulation is sensitive to weather indices during reproductive, flowering and grain-filling stages. A research study validated this relationship using the AquaCrop Model, which underwent Bayesian calibration involving meteorological, soil, crop, and management parameters (Zhang et al., 2018, 2019). Hence, these parameters (biomass and weather indices) were shortlisted and categorized in the BBN structure (Table 3).

**Table 3:** BBN model variables: parameters and categories.

Input variables	Intermediary variables	Output variables
Tillage system Soilscape Rainfall (mm) Temperature (C°)	Carbon-soil organic matter (kg/ha) Available water space (cm^3^ cm^3^) Precipitation interception (mm) Biomass (kg/ha)	Crop yield (kg/ha) Surface runoff (mm) GHG-CO_2_ emissions (kgCO_2_eqv/ha)

The BBN model was developed based on a few assumptions, such as weather indices with SOC influencing wheat crop yield (Droste et al., 2020). Similarly, different tillage practices influence available water space and change in SOC contents over time (Sharma et al., 2016). Temperature intensity not only affects crop development & growth rate for biomass and influences GHG-CO_2_ emissions (Tu and Li, 2017). Rainfall affects biomass production through water stress in drought conditions and can generate surface runoff where extreme rainfall occurs. Higher organic carbon content levels in soils increase soil fertility to generate higher crop yields, but lower OC levels affect the soil’s water-holding capacity. Soil textural compositions with higher rocks limit available water space in soils to impact surface runoff. Higher biomass intercepts precipitations to reduce surface runoff and deliver higher crop yield (Refer to details in Supplementary Tables S6, S7). Hence, the most pertinent variables were included in the BBN.

The BBN model included 11 nodes (variables), 17 links (relationships), and 2,800 conditional probabilities in the final BBN model structure, as shown in Figure 1. The structure of the BBN model is informed by evidence from published literature regarding variable relationships and their practical applications, as reported and utilized in other software. For example, a few software utilize crop growth simulation methods in hierarchical models, expressing these relationships through mathematical equations in the respective tools (Ahmed et al., 2020). These software functions on their fixed models for executing crop simulations. The DSSAT software uses the Crop Environment Resource Synthesis (CERES-Wheat) model to simulate wheat crop growth to climate, soil, and management, etc. and uses the default module, namely CERES-Godwin for soil organic matter and CENTURY model, to simulate SOC dynamics (Ahmed et al., 2023). A few modules and sub-modules for Actual CO_2_, Mauna Loa, Hawaii (Keeling curve) are used for daily records of global atmospheric carbon dioxide concentration and others for soil conservation service for infiltration, modified soil profile for soil layer distribution, etc. (Keeling, 1986). Tillage practices impact soil carbon dynamics by altering soil structure, aeration, and microbial actions, which induce CO_2_ emissions. The variable interface for the primary and sub-module in DSSAT is summarized in Supplementary Table S6. Then, the model executes to generate simulations after making an experimental set-up with all modules fed with the required input datasets and files.

In DSSAT, crop models can simulate single cropping and subsequent cropping systems. The soil water balance is based on a formulation by Ritchie methods, applying the concept of a drained upper limit (DUL) and drained lower limit (LL) for available soil water. The water accounting procedure for each layer in the soil profile applies to the DSSAT by Porter and Ritchie methods. Water movement from an upper layer cascades to lower layers, resembling a series of linear reservoirs. The difference between rainfall and runoff calculates the infiltration. The drainage process between layers occurs if soil water in the layer exceeds its water-holding capacity. Root water uptake drives the upward flow due to transpiration and soil evaporation. Available soil water determines the potential root water extraction and the root length density of each layer in the soil (Ritchie et al., 1984; Parton et al., 1994; Ritchie et al., 1998; Suleiman and Ritchie, 2004; Boote et al., 2008; Porter et al., 2010; Asseng et al., 2013; Howe, 2015; Khosravi et al., 2022). The study included a parameter, “available water space,” and calculated that using figures of total available water in soil profile minus field capacity in entire simulations for all seasons (Cassel and Nielsen, 1986). This study also explored the DSSAT and Aqua Crop modeling tools manuals, confirming the underlying mechanisms and the variables’ conditional relations in delivering simulations and model outputs. Some models exhibit identical relationships between variables to give respective model outputs. For instance, the simulation of crop yield relies on biomass. The conditional relationships were identified and confirmed through literature and recorded in Supplementary Table S7. The next step was to execute the DSSAT model.

Data synthesis was achieved through the DSSAT tool that generated numerous simulations executed for 30 individual soils, each for 48 years, and a summary is described in Table 2. The DSSAT tool generated multiple variable responses based on its predefined algorithms for modular and sub-modular functions. However, this research explored only specified variables identified for their potential responses in comparing tillage preference choice in relation to wheat yield, surface runoff, and GHG-CO_2_ emissions. The main user interface of the DSSAT tool was used to run the adapted model for simulations through window command selection. An information alert, “Simulations are completed,” popped up shortly with an option to click the next tab to access the analysis if the process is completed successfully. Alternatively, error messages and alerts may appear to navigate the inaccuracy for model calibrations at the appropriate modules, such as Xbuild, Sbuild, Weatherman, etc., and run the program again to complete the process successfully. A flowchart illustrating the process of generating simulated datasets in the DSSAT main interface, resulting in the creation of DSSAT OUTPUT files, is included as an annexure to Supplementary Table S6. This DSSAT data served as input data for the parametrization of the BBN (Jones et al., 2011).

Hence, the DSSAT served as a data simulator only, which provided a range of variable responses to soil attributes, weather, and crop management practices depicted in the Pang catchment area. Only pertinent variables and their responses were accessed following the DSSAT simulation compilations. This phase retrieved the system-generated output files (GHG.OUT, PlantGr2.OUT, PlantGro.OUT, SoilOrg.OUT, SoilWat.OUT, Weather.OUT) using the analysis tab and GBuild module (a plotting tool for data visualization). These output files contained the simulated datasets for all variables in the BBN model. This approach resulted in retrieving a total of 1,440 simulations. The acquired datasets were then formatted into a specified case file to parametrize the BBN.

Moreover, this study utilized the Netica software and its user interface to construct and parametrize the BBN model. The structural construction started from one variable (independent) as a parent node to the second (dependent) as a child and connected them based on their conditional relationship. All parent nodes are connected to their child nodes through an arrow link directing a conditional relationship from the parent to the child nodes by avoiding having a cycle in the network. This structure developed into a directed acyclic graph (DAG), a BBN model. All 11 nodes with 17 links were connected in a BBN and were now ready to parametrize using simulated datasets. The specified case file containing (the DSSAT outputs for specified variables) is used to parametrize the BBN model. The study applied the “Holdout Method” for model training and testing for validation. Therefore, seventy-five (75%) data were used for training the model by parametrization, as exemplified in the Supplementary Table S11. The assessment used this to populate the BBN model against unseen holdout datasets for the model, and the remaining twenty-five (25%) were then used to validate the BBN model by testing. The case files for the Holdout method’s application through 25% of datasets are available as specimens in Supplementary Table S12. The study also compared model validation results against error rates among “K-Fold Cross Validation” and “Train Test” Methods (Yadav and Shukla, 2016). The summary is highlighted in Table 4.

**Table 4:** Comparison of error rates among various model validation and testing methods.

Model output variable of interest	Model validation (Holdout Method)	K-fold cross validation (K-Fold CV Method)					Model testing
	Train test split Train = 75% (1975–2011) Test = 25% (2012–2022) Error rate	K-Fold Cross Validation CV– K1 (1975–1984) Error rate	K-Fold Cross Validation CV – K2 (1985–1994) Error rate	K-Fold Cross Validation CV – K3 (1995–2004) Error rate	K-Fold Cross Validation CV – K4 (2005–2014) Error rate	Prediction Error/ Average (Error rate)	Train (K1 + K2 + K3 + K4) (1975–2014) Test (K5) (2015–2022) Error rate
GHG-CO_2_ Emission (GHG) (Kg[CO_2_ eq]/ha)	29.39%	31%	36.33%	27.33%	31%	31.42%	25.42%
Runoff (ROFC) mm	33.64%	39%	47.33%	29.67%	36%	38%	20.83%
Crop yield (HWAD) Kg/ha	43.94%	56.33%	36.33%	40%	41.33%	43.50%	31.67%

The model yielded consistent and comparable results, evident through training with data from 1975 to 2014 (K1 + K2 + K3 + K4) and testing with data from 2015 to 2022 (K5) (Karimi et al., 2021; Mahmood et al., 2023).

The parametric details of the BBN model depend upon the relationships between parent and child nodes and their states defined for each variable in the network and their corresponding datasets (Supplementary Table S8). This aspect depends upon the nature and quality of datasets used to parametrize the BBN model. This study used synthetic datasets generated by the DSSAT tool, which delivered variable output responses following specified functions of certain phenomena, such as the pedotransfer function for soil water dynamics and CERES-Godwin and CENTURY-Parton SOC simulation. The probabilistic estimates for quantified variables in the BBN model were well-aligned with the known and established responses for the assessed parameters. The results highlight the adaptation of conservational tillage can lead to sustainable crop yield, reduced runoff, and GHG-CO_2_ emissions by offering precipitation interception and available water space in soils (Rahman et al., 2021).

### 2.3 BBN model outputs

The output variables of the BBN model are wheat crop yield, surface runoff and total GHG-CO_2_ emissions. The response of output variables is the reflection of model input variables and their vital interactions with associated variables based on their identified conditional phenomena in a hierarchical order, as shown in Figure 1. Results found model output variables have exhibited seasonal variations in their responses through long-term simulations. Weather indices have an imminent influence over crop yield, surface runoff, and total GHG- CO_2_ emissions. Tillage preference for no-till or conventional methods has a comparable influence over SOC levels in the soils cultivated over extended periods. Tillage practices have profound effects on carbon sequestration and preserving soil structure. This investigation evaluated the model performance and validated that using test cases. The study also analyzed posterior probability distributions of interacting variables to assess their comparative impacts on each other.

### 2.4 The BBN model performance and evaluation procedure

Various metrics are applied to evaluate the performance of the BBN model to measure uncertainty (Table 5). These include model complexity (e.g., the number of nodes, links, etc.) (e.g., scenario evaluations) and sensitivity analysis (e.g., sensitivity to findings, case file simulation, etc.) using Netica software (Marcot, 2012; Namdari and Li, 2019).

**Table 5:** Evaluation metrics (https://doi.org/10.1016/j.ecolmodel.2012.01.013).

Evaluation metrics	Use/Application	Caveats and assumptions	Manuscript reference
Model Sensitivity and influence			
Variance reduction (sensitivity to finding)	Applied to all response variables (continuous) variables	Input variables are set to their default priori probabilities unless specifically desired otherwise.	Section 2.4.3.
Case file simulation (model validation)	Analysis of covariation between input variables and output variables probability distributions.	Simulated cases cover all covariation conditions with an adequate sample size.	Section 2.5.
Influence analysis (scenario evaluation)	Determines incremental effects of selected inputs set to best, worst, or other specified values.	Best used for scenario analysis.	Section 2.4.2. Figure 2
Model complexity			
Number of variables (model complexity analysis)	Determine degrees of freedom	Important to include vital variables, including latent variables.	Section 2.4.1. Figure 1, Table 3, and Supplementary Figure S5.
Number of node states (model complexity analysis)	Affects model precision and the overall number of probability values in the model	Count the number of states after discretizing continuous functions to the desired precision.	Section 2.4.1. Supplementary Table S8.
Number of conditional probabilities (model complexity analysis)	Sensitive to model structure, including variable connections and precision.	Doest not include prior (unconditional) probability tables; one could include them if desired.	txt file of Conditional Probability Tables (CPTs) - 75% data-(1975–2011) of Supplementary material.
Model prediction performance			
Confusion matrix/ error (model validation)	Depicts rate of Type I and Type II errors in classification or prediction models.	Typically based on the highest probabilities state, which may over simplify the model’s utility if other results could be equally useful.	Section 2.5. Tables 6–8
Logarithmic loss value Spherical payoff Brier score Quadratic loss (model validation)	Indexes performance of classification models.	Influenced by several states in the response variable.	Section 2.5. “Scoring rule results” Tables 6–8

**Figure 2:**
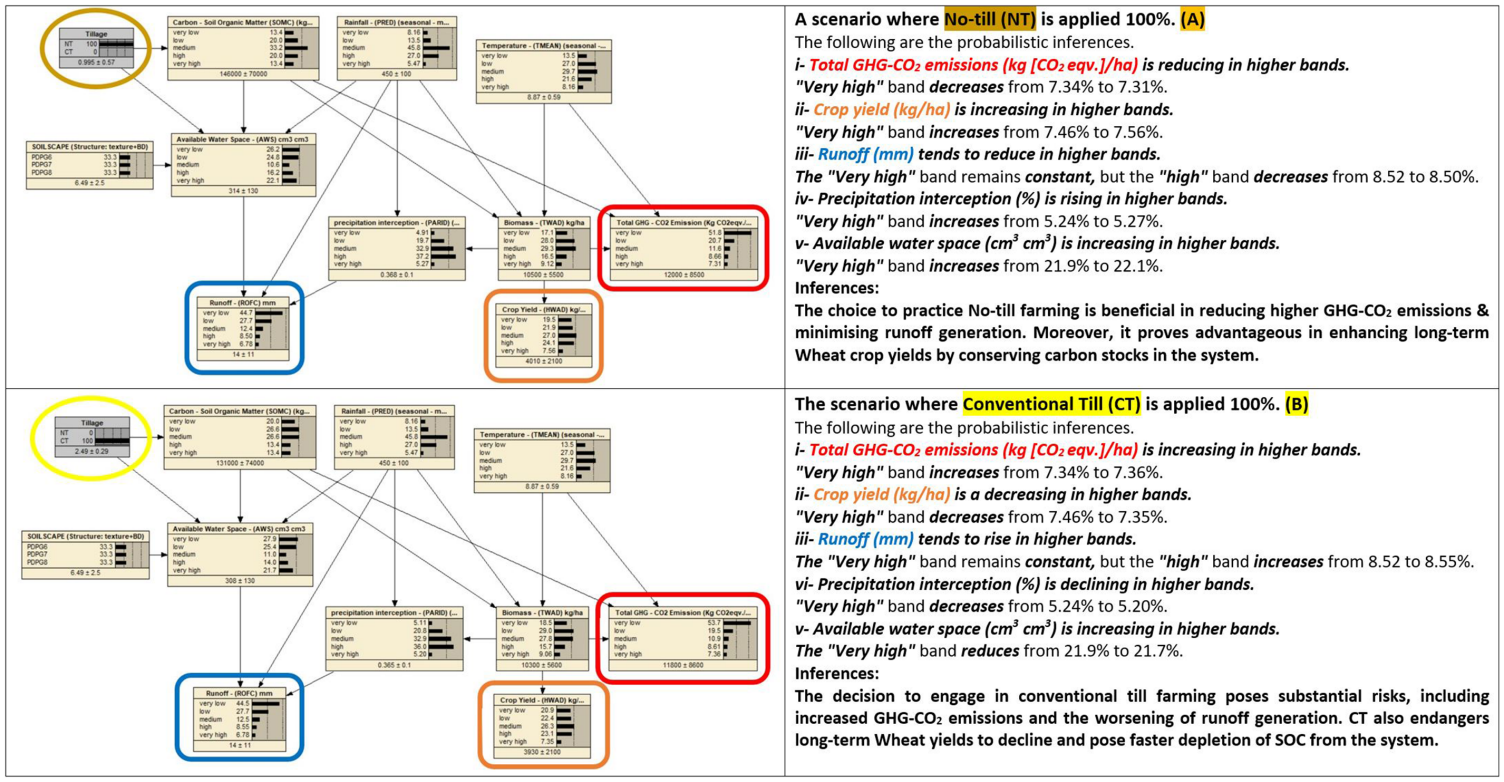
This comparison shows an influence analysis of the BBN model evaluation based on comparing two scenarios, e.g., **(A,B)**, one with favorable and the other with unfavorable effects outcomes.

#### 2.4.1 Model complexity analysis

The BBN model network is a directed acyclic graph, and complexity depends upon the number of variables and their interactions included in the BBN model; e.g., many nodes with numerous links/ arrows can make a BBN model more complex and inefficient. The BBN model consists of 11 nodes (variables), 17 links (conditional relationships), and 2,800 conditional probabilities learnt through parametric learning from simulated datasets by the DSSAT (Thogmartin, 2010). The number of discretized states of continuous variables also determines model complexity, as highlighted in Supplementary Table S8. The BBN model network contains the following junction trees with member nodes, as shown in Supplementary Table S9. A Clique is a set of nodes connected to another set of nodes, and clique size represents a complete subgraph of a given graph which contains the maximum number of nodes, e.g., vertices and edges in a graph (Almond and Almond, n.d.). Hence, this BBN model is neither very complex nor very simple shown in Figure 1 and Table 3.

#### 2.4.2 Influence analysis

Influence analysis evaluates posterior probability distributions from chosen input variables and results in good-to-best or bad-to-worst-case scenario values. This information helps in decisions by measuring individual or set input variables’ impact on outcomes, as shown in Figure 2. Probabilistic inferences are illustrated on uncertainty estimates measured in 0–100% percentages. These probabilities represent the likelihood or confidence level associated with different states or outcomes of the variables in the model (Hájek, 2002). It’s important to note that even small changes in certain influential factors or inputs can lead to variability in probabilistic estimates. Model parametric changes can also reveal fluctuations in these values. Sensitivity analysis and other evaluation metrics were performed to highlight this and described in the relevant sections.

#### 2.4.3 Sensitivity analysis

Using Netica, the BBN model performed a sensitivity analysis called sensitivity to findings for GHG-CO_2_ emissions, runoff, and yield. This analysis ranked and ordered the input variables in the Supplementary material, providing a results list in Supplementary Tables S10a–c. Tabulated variables are listed in descending order regarding the above variables (output variables) as per the generated report by Netica. Variance reduction, mutual information, and variance in belief values are reduced in descending order, depicting the comparable sensitivity of each variable with that of subject (output) variables (Supplementary Tables S10a–c). This method quantitatively compares the variables’ ability to reduce uncertainty or variance (entropy reduction) and allows for mutual comparisons (Thogmartin, 2010; Marcot, 2012; Namdari and Li, 2019).

### 2.5 Model validation procedure

The BBN model is validated using simulated datasets due to the scarcity of real data availability. Access to real-world data sources, resource constraints, and complications linked to data protection are common challenges in most modeling works. Establishing a long-term tillage-based experiment in competing time frames with challenges that arose due to COVID-19 pandemic implications has prompted this shift toward assessing data through simulation methods. This approach brings some limitations while using the simulated dataset for model validation. These may not capture the adaptation in real-world situations, face result validation concerns, have limited capacity in capturing factual variability, and carry bias embedded in algorithms defined in tools used for data simulations. However, simulated datasets offer a great opportunity to explore complex scientific systems where access to real-world data is a valid concern. This approach presents probabilistic solutions and diverse scenarios for comparing consequences, which could support decision-making for the right choices.

The assessment employed 25% of the synthetic dataset obtained from the DSSAT-generated simulations to test the BBN model for performing model validation in this research (Morgan and Granger and Max Henrion, 1990; Netica, 2024). The test cases representing datasets are used for response variables for 2012–22, the specimen listed in Supplementary Figure S7. This approach is also known as the holdout method for model validation (Jones et al., 2011). Confusion matrices GHG-CO_2_ Emissions, Runoff, and Crop yield are tabulated in 6, 7, and 8, respectively. Error rate illustrated two types of error by the BBN model, e.g., type 1 error for false positive, which means rejecting a true hypothesis, and type 2 error means failing to reject a false hypothesis. The sum of these is reflected in the confusion matrix.

The study utilized Netica software to evaluate the model’s performance, which applied scoring rules based on its system-generated computations. The scoring rules are the model evaluation metrics and assess the model’s performance of probabilistic estimates or predictive distributions. These rules measure the predicted distribution, the observed outcome, and the model’s accuracy and calibration. Netica software retrieved the report with a confusion matrix carrying the scoring rule outcomes of the individual output variable. Scoring rule outcomes highlighted the actual beliefs of the states that followed the values in the case file. Results found three types of scoring rules (Logarithmic loss, Quadratic loss, and Spherical loss) performed by Netica software (Pearl, 1978; Netica, 2023).

Logarithmic loss value was calculated based on a natural log and ranged from zero to infinity, where zero represents the best performance at an acceptable level in all the above cases. For instance, the Logarithmic loss value is depicted as 0.9555 from the confusion matrix (for GHG-CO_2_ emissions) under Table 6. This value is closer to zero than infinity and declares an acceptable performance level. Logarithmic loss penalizes incorrect predictions more seriously when they are confidently wrong. Hence, the Logarithmic loss value was calculated based on a natural log and ranges from zero to infinity, where zero represents the best performance and having the value of 0.9555 is an acceptable level of model performance in the given example.

**Table 6:** Confusion matrix [For GHG Emissions – as CO_2_ equivalent Kg (CO_2_ eq)/ha].

Predicted						
Very low	Low	Medium	High	Very high		
220	8	4	0	0	Very low	Actual
60	10	0	0	0	Low	
17	1	3	0	0	Medium	
3	1	1	0	0	High	
1	1	0	0	0	Very high	

Error rate = 29.39% (Model predicted accurately 70.61% of the provided cases). Scoring rule results: Logarithmic loss = 0.9555; Quadratic loss = 0.4859; Spherical loss = 0.7122.

Quadratic loss provides another metric to evaluate the performance of a model and is also known as the Brier score and ranges from zero to 2, where zero is the best. Quadratic loss penalizes larger errors more severely than smaller errors. Therefore, this value becomes sensitive to outliers, as larger errors contribute disproportionately to the overall loss. The quadratic loss value was depicted as 0.4859 from the confusion matrix (for GHG-CO_2_ emissions) under Table 6. This value is closer to zero and sets an acceptable level for the model performance. Spherical payoff is another matrix to measure the performance of the BBN model by considering the accuracy and calibration of predictions. This scoring value is linked to the balanced risk–reward profile of a sphere. The spherical payoff scoring rule aims to reward the model for achieving high accuracy through making correct predictions following a well-calibrated model for accurate estimates of the likelihood of those predictions. This matrix measures the model performance using an index ranging [0,1], where 1 is the highest value for illustrating better model performance. In the above example, the Spherical loss value was depicted as 0.7122, which is closer to one and considered an acceptable level for the model performance. Similarly, this BBN model performed better than average for all output variables in dealing with the nuances of probability values under scoring rule results as depicted through the Confusion matrix (for Runoff) and Confusion matrix (for Crop yield) and shown under Tables 7, 8, respectively (Marcot, 2012; Netica, 2023).

**Table 7:** Confusion matrix (For Runoff – ROFC - mm).

Predicted						
Very low	Low	Medium	High	Very high		
168	33	0	0	0	Very low	Actual
45	50	0	0	0	Low	
1	23	1	0	0	Medium	
0	3	1	0	0	High	
0	4	1	0	0	Very high	

Error rate = 33.64% (Model predicted accurately 66.36% of the provided cases). Scoring rule results: Logarithmic loss = 0.9443; Quadratic loss = 0.4885; Spherical loss = 0.7101.

**Table 8:** Confusion matrix (For Crop yield – kg/ha).

Predicted						
Very low	Low	Medium	High	Very high		
29	6	0	0	0	Very low	Actual
8	72	16	0	0	Low	
0	29	36	26	0	Medium	
0	0	26	48	0	High	
0	0	1	33	0	Very high	

Error rate = 43.94% (Model predicted accurately 56.06% of the provided cases). Scoring rule results: Logarithmic loss = 0.9699; Quadratic loss = 0.5732; Spherical loss = 0.6533.

However, the model was also validated using the K-Fold cross-validation method and tested through the train split approach. The comparative results are summarized in Table 4. The details are provided in the Supplementary material S1. Moreover, the multiple regression model was employed in this analysis and findings are detailed as Supplementary material S2. Such comparisons evaluate the modeling performance among different models, such as multiple regression and network models and promote the use of the business value approach (Bansal et al., 1993).

## 3 Results and discussion

### 3.1 Posterior probability distribution analysis

The BBN model was developed to integrate prior knowledge using simulated datasets. It highlights analyzing model inferences through probabilistic estimates represented by the modeled parameters. Salient features are described below. The BBN model captured uncertainties linked to seasonal variations by providing posterior probability data distributions reflecting probabilistic relationships across all interacting variables in the model and offering decision choices for competing tillage preferences. This study analyzed the posterior probability distribution of intermediary and output variables in the BBN model. This evaluation technique revealed the performance of the best and worst-performing variables individually and on average (Solheim, 2021).

#### 3.1.1 Wheat crop yield

**Figure 3:**
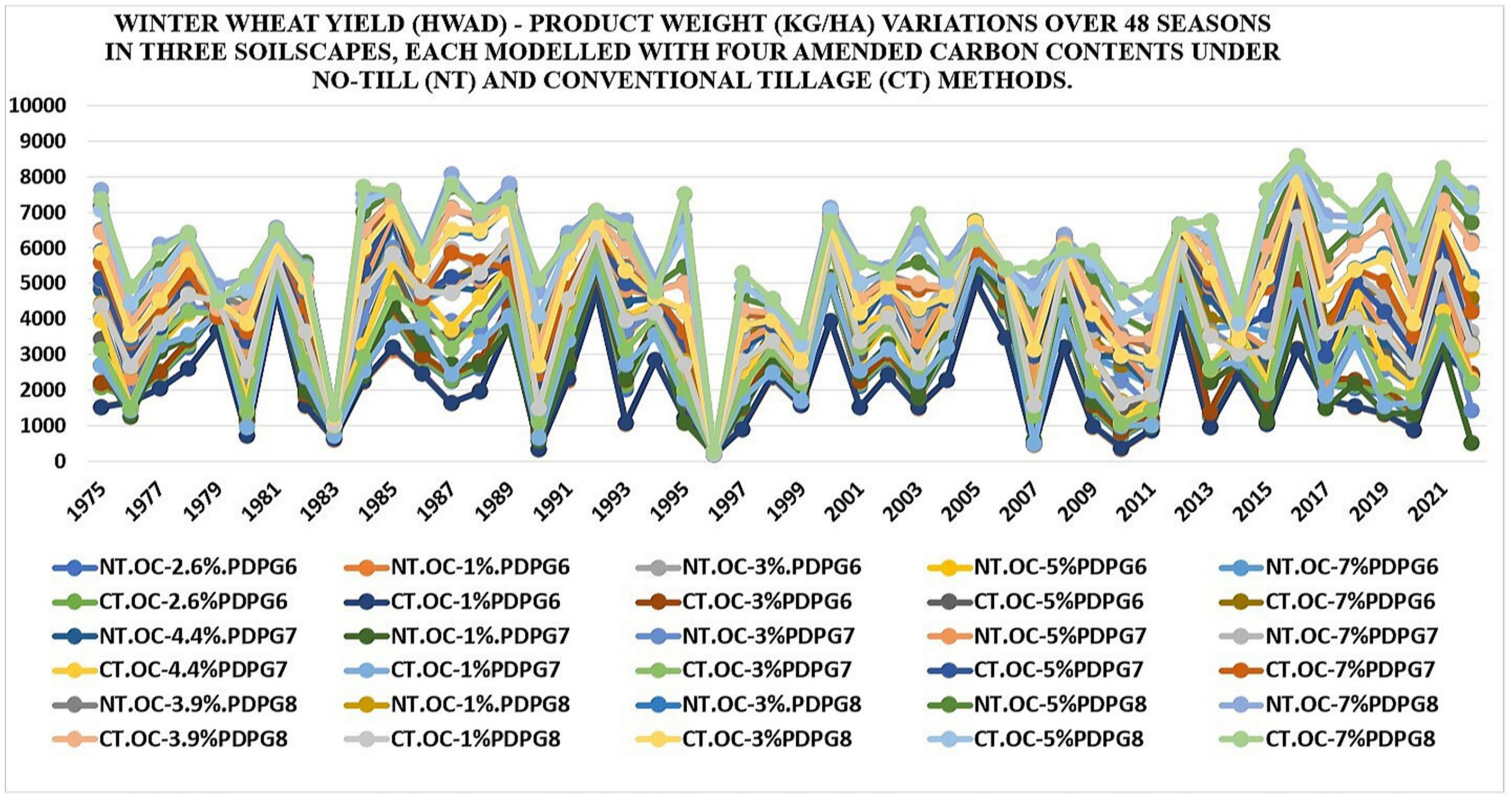
Seasonal variation in Wheat yield (kg/ha) in 30 soils under no-till versus conventional till using simulated data from DSSAT v4.8 since 1975–2022.

**Figure 4:**
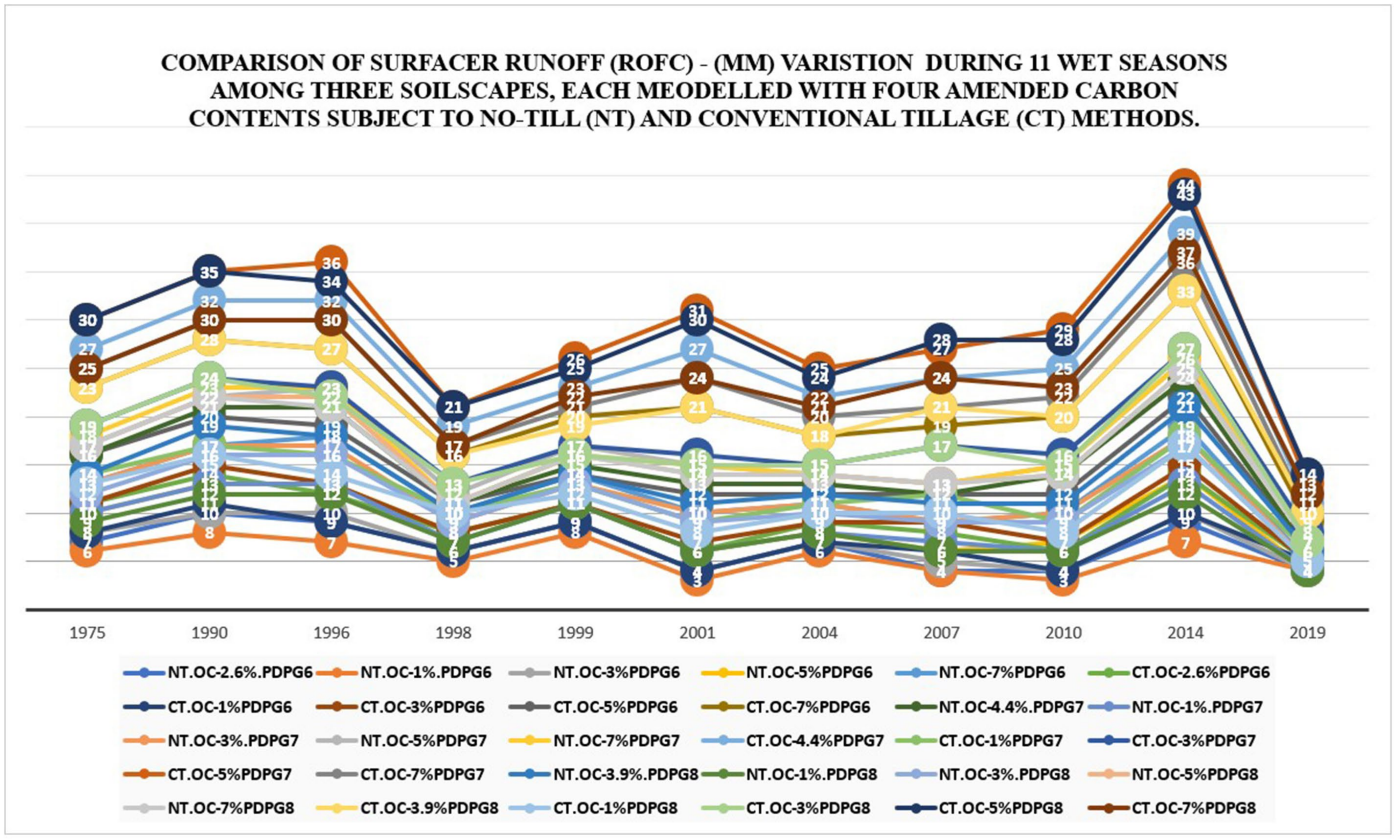
Comparison of cumulative surface runoff (mm) in 11 wet seasons among 30 soils under no-till versus Conventional till using simulated data from DSSAT v4.8 since 1975–2022.

Wheat crop yield varied in quantitative results among three real soils and their four modeled soils, each under No-till versus Conventional till cultivations using simulated data generated in the DSSAT v4.8 for over 48 years. Results found the maximum product weight (kg/ha) in the “NT.OC-7%PDPG8,” e.g., 8,575 kg/ha in the year 2016 and the minimum product weight (kg/ha) in “CT.OC-1%PDPG6,” e.g., 174 kg/ha in 1996 (Refer to Figure 3; Table 9). This year is ranked among the top three wettest seasons in the entire time series (Refer to Figure 4) and correlates with lower wheat production in soils with lower levels of SOC. Similarly, “CT.OC-7%PDPG8” averaged the highest product weight at 6,008 kg/ha, while “NT.OC-1%.PDPG6” averaged the lowest at 2,087 kg/ha. Therefore, the No-till method produced the highest wheat crop yield in soils with 7% SOC from PDPG8, while the conventional till method yielded the minimum with 1% SOC from PDPG6. Results are aligned with several research findings (Soane et al., 2012; Nunes et al., 2018). Seasonal variation in Wheat yield (kg/ha) is available in Figure 3 with basic statistics highlighted in Table 9.

**Table 9:** Statistical analysis of wheat yield (kg/ha) distribution in 30 soils under No-till versus Conventional tillage - Refer to Figure 3.

Statistical description	NT.OC-2.6%.PDPG6	NT.OC-1%.PDPG6	NT.OC-3%.PDPG6	NT.OC-5%PDPG6	NT.OC-7%PDPG6	CT.OC-2.6%PDPG6	CT.OC-1%PDPG6	CT.OC-3%PDPG6	CT.OC-5%PDPG6	CT.OC-7%PDPG6
Minimum	203	175	208	231	258	205	174	211	235	258
Maximum	5,541	5,004	5,682	6,606	7,750	5,645	5,035	5,739	6,701	7,792
Average	2596.54	2086.77	2695.19	3428.06	4271.63	2653.46	2097.46	2761.83	3560.04	4464.81
Standard deviation	1387.78	1245.66	1416.67	1533.17	1547.05	1397.90	1243.08	1424.07	1522.24	1495.05
Statistical description	NT.OC-4.4%.PDPG7	NT.OC-1%.PDPG7	NT.OC-3%PDPG7	NT.OC-5%PDPG7	NT.OC-7%PDPG7	CT.OC-4.4%PDPG7	CT.OC-1%PDPG7	CT.OC-3%PDPG7	CT.OC-5%PDPG7	CT.OC-7%PDPG7
Minimum	246	208	233	254	272	230	175	216	254	269
Maximum	6,970	5,490	6,246	7,275	7,794	6,728	5,668	6,278	7,265	7,860
Average	4046.69	2617.44	3503.08	4190.92	4569.29	3615.58	2793.96	3270.88	4416.08	4717.71
Standard deviation	1564.87	1459.59	1545.57	1602.86	1563.11	1646.17	1393.91	1546.03	1455.57	1431.53
Statistical description	NT.OC-3.9%.PDPG8	NT.OC-1%.PDPG8	NT.OC-3%.PDPG8	NT.OC-5%PDPG8	NT.OC-7%PDPG8	CT.OC-3.9%PDPG8	CT.OC-1%PDPG8	CT.OC-3%PDPG8	CT.OC-5%PDPG8	CT.OC-7%PDPG8
Minimum	242	205	233	244	266	235	206	234	246	265
Maximum	8,127	6,861	7,883	8,332	8,575	8,122	6,853	7,877	8,316	8,562
Average	5286.73	3918.19	4897.33	5602.35	5967.17	5266.19	3907.96	4884.63	5724.54	6008.44
Standard deviation	1585.03	1561.85	1563.45	1619.49	1614.27	1572.85	1565.88	1555.29	1606.07	1602.82

#### 3.1.2 Comparison between observed data and simulated data of wheat yield (kg/ha) among 30 soils under no-till versus conventional till from 2016 to 2021

**Figure 5:**
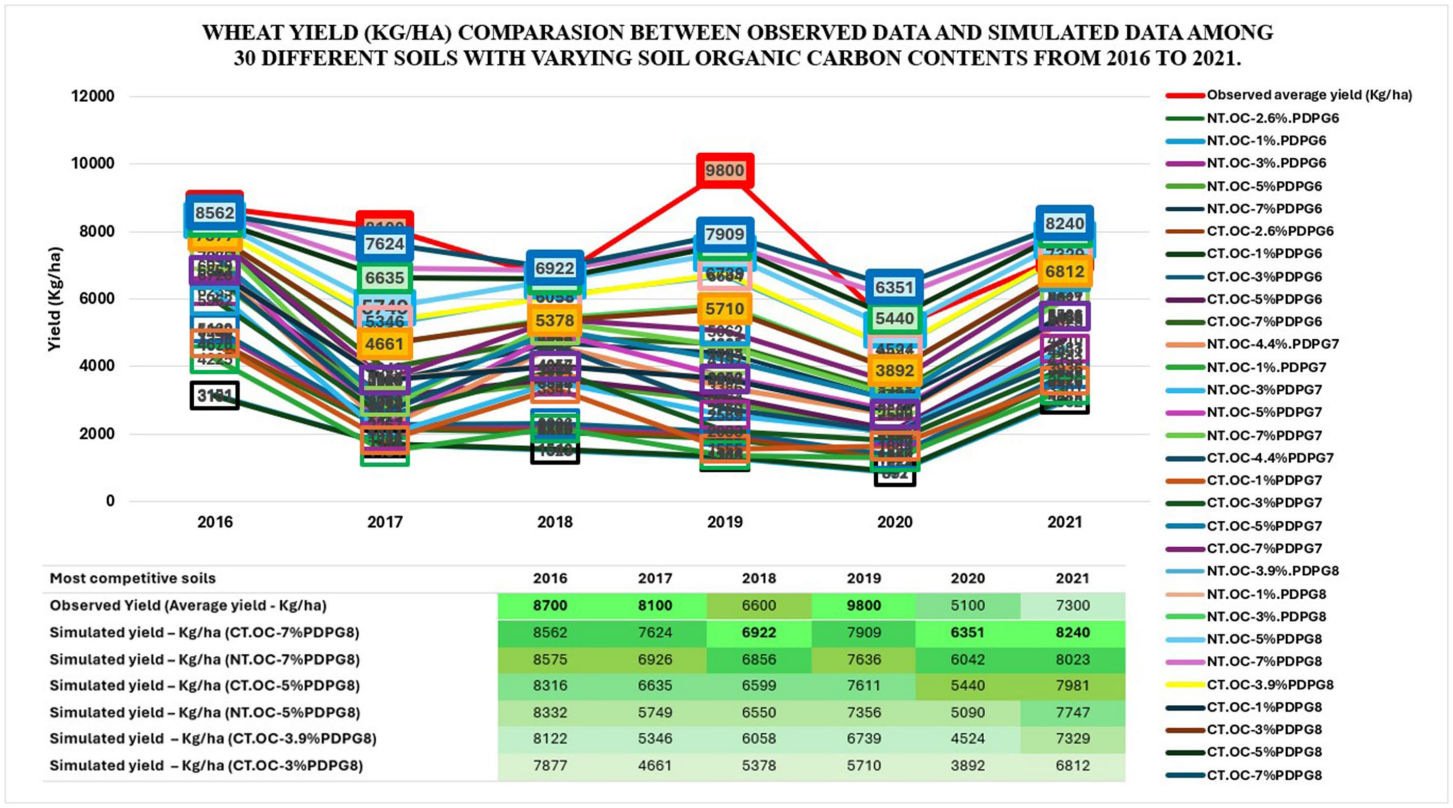
Comparison between observed data and simulated data of wheat yield (Kg/ha) among 30 soils under No-till versus Conventional till since 2016–2021.

Wheat yield was compared between observed and simulated data among 30 soils under No-till versus Conventional till from 2016 to 2021. Observed data of average wheat yield from the same catchment was tracked from published data (Boote et al., 2008). The comparison showed that soils with higher carbon content levels showed higher yields overall. PDPG8 soils demonstrated strong performance, closely aligning with the observed average yield. However, the observed yield showed the highest produce level in 2016, 2017 and 2019, followed by PDPG soils with higher soil carbon content levels from 3 to 7% among NT and CT practices in all simulations. The PDPG8 soil with organic carbon contents of 7% outperformed in 2018, 2020 and 2021 by yielding 6,922 kg/ha, 6,351 kg/ha, and 8,240 kg/ha, respectively. However, the soils “CT.OC-7%PDPG8 & NT.OC-7%PDPG8” consistently produced sustainable yields over the competing period. The observed and simulated data for the competing soils are depicted by comparative wheat yields in Figure 5. Another study presented a probabilistic model for improved forecasting of crop yield under environmental uncertainty. (Supit, 1997; Mahmood et al., 2023)

#### 3.1.3 Surface runoff

**Figure 6:**
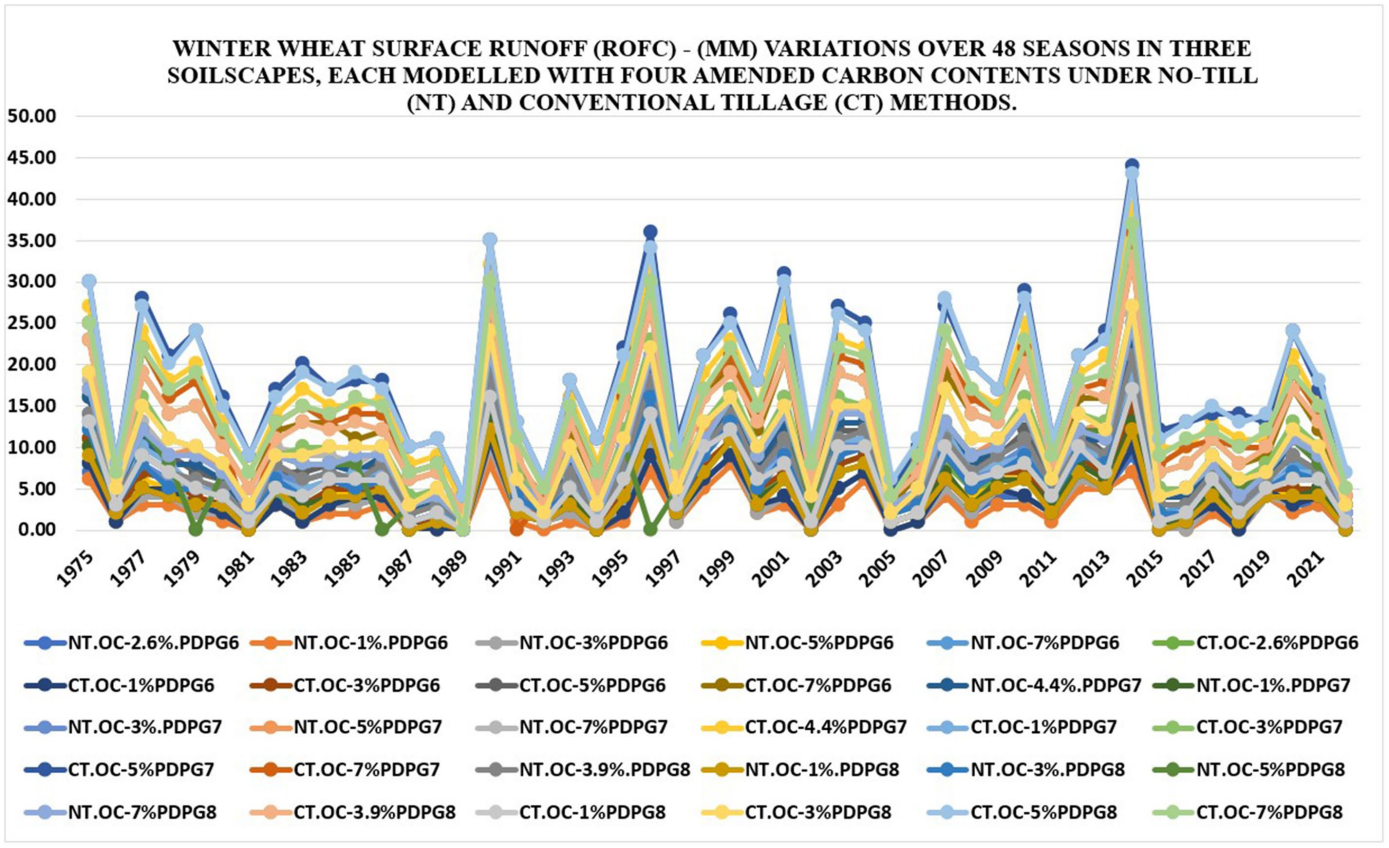
Seasonal variation in surface runoff (mm) in 30 soils under no-till versus conventional till using simulated data from DSSAT v4.8 since 1975–2022.

Cumulative runoff (mm) varied in quantitative results among all real and modeled soils under No-till and Conventional tillage, simulated over 48 years with DSSAT v4.8. The highest cumulative runoff of 44 mm was recorded in “CT.OC-5%PDPG7” in 2014, a year characterized by flooding. In the same year, “NT.OC-5%.PDPG7” and “NT.OC-7%.PDPG7” both experienced a maximum runoff of 26 mm. Among 30 soils, 18 had a seasonal minimum runoff of 0 mm. On average, “CT.OC-5%PDPG8” had the highest cumulative runoff (18.15 mm), while “NT.OC-1%.PDPG6” had the lowest (2.38 mm). Seasonal variation is evident in Figure 6, with basic statistics in Table 10. These findings are comparable with other studies (DeLaune and Sij, 2012). This indicates that NT results in decreased runoff in soils with both higher and lower organic carbon levels. This response assumes that higher SOC levels could no longer help water retention after reaching saturation in a high rainfall season. This aspect highlights the level of resilience or vulnerability to challenging surface runoff characteristics among soils with high organic carbon (OC) levels under conventional tillage (CT).

**Table 10:** Statistical analysis of surface runoff (mm) distribution in 30 soils under No-till versus conventional tillage - See Figure 6.

Statistical description	NT.OC-2.6%.PDPG6	NT.OC-1%.PDPG6	NT.OC-3%.PDPG6	NT.OC-5%PDPG6	NT.OC-7%PDPG6	CT.OC-2.6%PDPG6	CT.OC-1%PDPG6	CT.OC-3%PDPG6	CT.OC-5%PDPG6	CT.OC-7%PDPG6
Minimum	0	0	0	0	1	0	0	0	1	2
Maximum	10	8	10	14	19	14	11	15	22	33
Average	3.04	2.38	3.19	4.52	6.63	4.75	3.38	5.02	7.73	12.71
Standard deviation	2.71	2.30	2.86	3.51	4.43	3.64	2.91	3.76	4.99	6.87
Statistical description	NT.OC-4.4%.PDPG7	NT.OC-1%.PDPG7	NT.OC-3%PDPG7	NT.OC-5%PDPG7	NT.OC-7%PDPG7	CT.OC-4.4%PDPG7	CT.OC-1%PDPG7	CT.OC-3%PDPG7	CT.OC-5%PDPG7	CT.OC-7%PDPG7
Minimum	1	0	0	1	0	3	0	1	0	0
Maximum	24	13	18	26	26	39	18	27	44	36
Average	8.48	4.38	6.33	9.46	9.23	15.83	6.46	10.23	18.10	13.88
Standard deviation	5.17	3.44	4.32	5.54	5.73	8.00	4.52	5.94	9.19	7.92
Statistical description	NT.OC-3.9%.PDPG8	NT.OC-1%.PDPG8	NT.OC-3%.PDPG8	NT.OC-5%PDPG8	NT.OC-7%PDPG8	CT.OC-3.9%PDPG8	CT.OC-1%PDPG8	CT.OC-3%PDPG8	CT.OC-5%PDPG8	CT.OC-7%PDPG8
Minimum	1	0	0	0	1	2	0	1	4	0
Maximum	21	12	17	25	25	33	17	27	43	37
Average	7.25	4.00	5.85	8.13	8.96	12.88	5.81	9.90	18.15	14.92
Standard deviation	4.68	3.30	4.10	5.46	5.34	6.86	4.15	5.88	8.46	7.53

#### 3.1.4 Comparison of cumulative surface runoff (mm) in 11 Wet seasons among three soilscapes under no-till versus conventional till methods using simulated data generated in the DSSAT v4.8 for over 48 years

The results show that in 2014, during a flooding year, “CT.OC-5%PDPG7” had the highest cumulative runoff of 44 mm over 11 wet seasons. Conversely, “NT.OC-5%PDPG6” had the lowest cumulative runoff at 3 mm in 2001 and 2010. On average, “CT.OC-5%PDPG7” had the highest cumulative runoff (28.82 mm), while “NT.OC-1%.PDPG6” had the lowest (5.55 mm) across all wet seasons. These findings align with previous research (Gautam et al., 2015). The comparison is depictable in Figure 4. There are strong implications of higher levels of cumulative surface runoff that can lead to soil erosion and deterioration of water quality. Runoff over the soil surface removes soil particles and sediments away from their location and becomes a cause of soil loss, which results in soil fertility loss and impacts soil health and productivity. Runoff carries substances and residuals of agrochemical pollutants to the hydrological system, which endangers the ecosystem’s balance. Hence, suitable tillage approaches such as reduced to no-tillage can help conserve soil aggregates’ moisture level and facilitate land cover by reducing evapotranspiration through retaining crop residuals.

#### 3.1.5 Carbon – soil organic matter

Soil organic matter represents all organic components of soil, while organic carbon (OC) refers to the carbon components within the SOM. Carbon in soil organic matter (C-SOM) varied in quantitative results across real and modeled soils under both No-till and Conventional tillage, simulated over 48 years with DSSAT v4.8. Seasonal changes in C-SOM were observed across all soils. Positive changes in C-SOM were found in soil “CT.OC-7%PDPG8” (e.g., +862 kg/ha in 2012), while negative changes were seen in “NT.OC-1%.PDPG7” (e.g., −200 kg/ha in 1996, a wet season). On average, “NT.OC-7%PDPG8” had the highest C-SOM (e.g., 637 kg/ha), while “NT.OC-1%.PDPG7” had the lowest (e.g., −151 kg/ha). These findings are comparable with other research findings (Pinheiro et al., 2015). Seasonal change in C-SOM is confirmed in Supplementary Figure S2. This response assumes that NT practices support higher organic carbon levels in soils. Carbon–soil organic carbon (C-SOM) is vital in maintaining soil health and carbon sequestration. C-SOM is not limited to its functional role in nutrient cycling, maintaining soil structure, enhancing water retention, facilitating microbial activity and soil biodiversity support. Tillage modifications can contribute to carbon sequestration by reducing soil carbon loss by minimizing the direct contact of soil particles and tilling equipment. Hence, with higher C-SOM levels in soils, soil resilience and resistance to soil erosion will result in better soil health conditions. Suitable C-SOM levels also support a balance between soil flora and fauna for increased soil biodiversity and facilities for ecosystem services.

#### 3.1.6 Comparison between minimum and maximum C-SOM (kg/ha) in 30 soils under no-till versus conventional till methods using simulated data generated in the DSSAT v4.8 for over 48 years

The maximum soil carbon content (C-SOM) of 286,634 kg/ha was found in “NT.OC-7%PDPG8” in 1984, while the minimum of 5,894 kg/ha was observed in “CT.OC-3.9%PDPG8” in 2009. These results indicate higher C-SOM levels in NT compared to CT cultivation (Ferreira et al., 2020). Analysis from 1975 to 2022 using DSSAT v4.8 simulated data showed that NT had a higher average minimum C-SOM (148,752 kg/ha) than CT (130,315 kg/ha). Conversely, NT contained 148,920 kg/ha more on average for maximum C-SOM, whereas CT contained 131,995 kg/ha. This information is evident in Supplementary Figure S3. The observed differences in C-SOM levels between different soil types and tillage methods have ecological implications for carbon sequestration, erosion control, soil health, soil biodiversity, nutrient cycling, and water quality. Hence, tillage methods which can facilitate these aspects objectively should be preferred and promoted. For example, reduced tillage, non-inversion, zero or conservational tillage could endorse achieving the above phenomena for advancing ecological stability.

#### 3.1.7 Comparison between minimum and maximum GHG-CO_2_ emissions (eqv. Kg/ha) among 30 soils under no-till versus conventional till methods using simulated data generated in the DSSAT v4.8 for over 48 years

**Figure 7:**
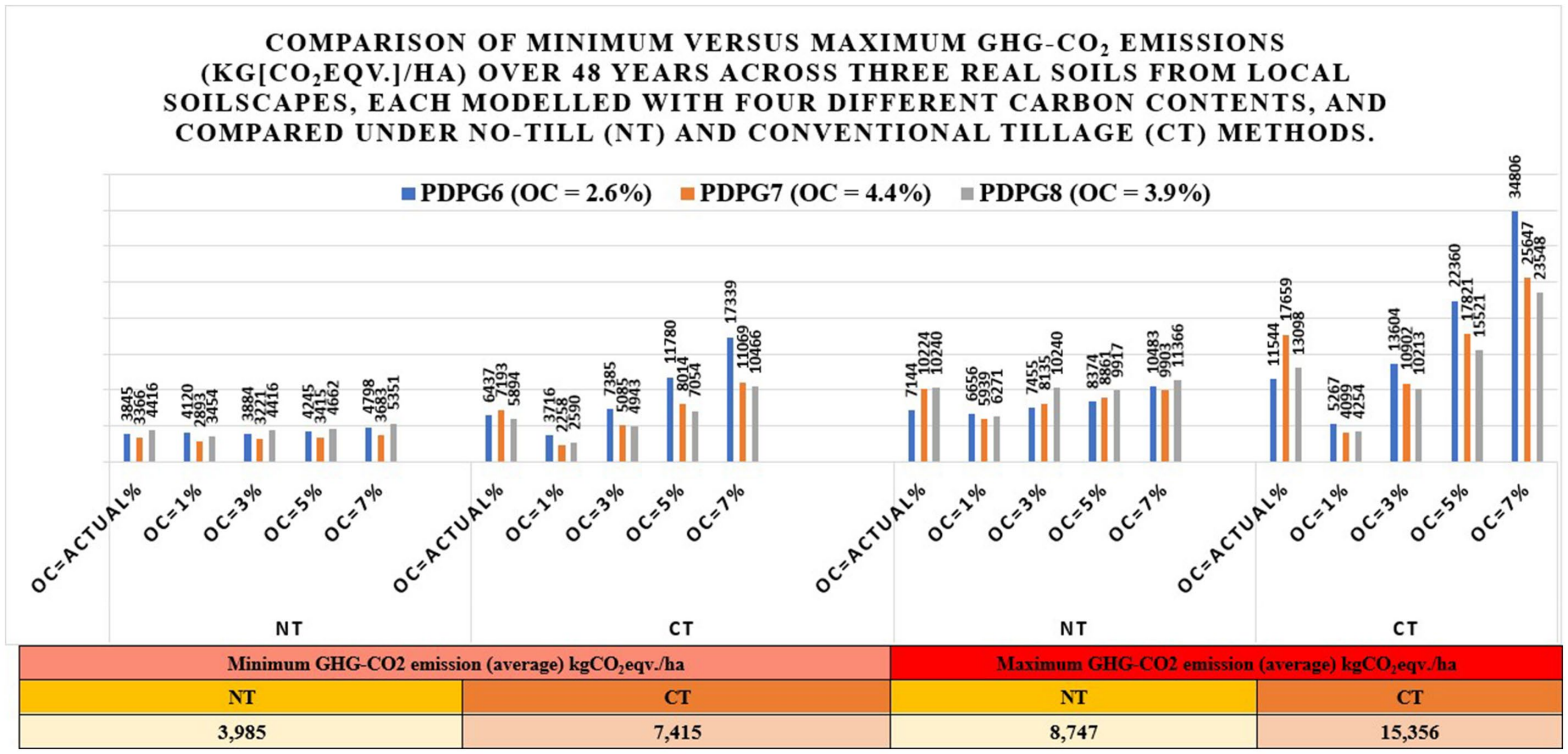
Comparison between minimum and maximum GHG-CO_2_ emissions (KgCO2eq./ha) among 30 soils under no-till versus Conventional till cultivation using simulated data from DSSAT v4.8 since 1975–2022.

Results found that “CT.OC-7%PDPG6” soil emitted the highest GHG-CO_2_ at 34,806 kgCO_2_eqv./ha in 1996, while “NT.OC-7%PDPG6” emitted 10,483 kgCO_2_eqv./ha in the same year. This information suggests higher emissions in soils with higher organic carbon content under CT cultivation and significantly lower under NT. Analysis from 1975 to 2022 using DSSAT v4.8 simulated data showed that NT had lower average minimum GHG-CO_2_ emissions (3,985 kgCO_2_eqv./ha) than CT (7,415 kgCO_2_eqv./ha). Conversely, NT emitted 8,747 kgCO_2_eqv./ha less average for maximum emissions, whereas CT emitted 15,356 kgCO_2_eqv./ha. These findings revealed that NT cultivation is better for having minimum and maximum GHG-CO_2_ emissions (total average scale) at reduced levels (Six et al., 2004; Mangalassery et al., 2014). The comparison is obvious in Figure 7. Soil organic matter contents, crop residue management, crop fertilization, soil moisture, soil temperature, and tillage intensity are the potential drivers of GHG-CO_2_ emission between different soils and tillage methods. However, suitable strategies can be adopted to mitigate them. These are not limited to adapting tillage modification (reduced to no-till), cover cropping (cropping between cash crops), crop rotation (rotating crops), agroforestry (tree planting in agri-landscapes), manuring and organic amendments (compost manuring), and precision agriculture (precision agriculture technologies).

#### 3.1.8 Available water space

Significant variation in available water space (cm^3^ cm^3^) was observed among three real soils and four modeled soils under No-till and Conventional till methods using simulated data from DSSAT v4.8 over 48 years. The maximum available water space was recorded in “CT.OC-7%PDPG8” at 528 (cm^3^ cm^3^) in 1996, followed by “NT.OC-7%PDPG8” at 520 (cm^3^ cm^3^) in the same year. In contrast, the minimum was observed in “NT.OC-1%.PDPG7” at 127 (cm^3^ cm^3^) in 2022. On average, “CT.OC-7%PDPG8” exhibited the highest available water space at 508 (cm^3^ cm^3^), while “NT.OC-1%.PDPG7” had the lowest average available water space at 138 (cm^3^ cm^3^). These findings show that soils with higher OC levels under CT and NT in both cultivations provide more available water space. However, the choice and management of tillage practices can particularly influence soil water dynamics, impacting water space availability in soils by altering soil structure, compaction, organic matter content, and surface cover to mitigate evaporation. That’s why Available Water Space (AWS) has the highest number of relationships (arc links) with other variables (nodes) in the BBN (see Figure 1). Additionally, AWS is sensitive to soil features and carbon-soil organic matter, as indicated in Supplementary Table S10d in the Supplementary material. Results are comparable with a similar study in the US (Hill, 1990). Seasonal variation in available water space (cm^3^ cm^3^) is illustrated in Supplementary Figure S4.

#### 3.1.9 Precipitation interception

Among the three real soils and their four modeled soils, precipitation interception (%) revealed significant variation under both No-till and Conventional till methods using simulated data generated in DSSAT v4.8 over 48 years. The maximum “precipitation interception (%)” was noted in soils labeled “NT.OC-7%PDPG8” and “CT.OC-7%PDPG8,” up to 0.59 percent in 2016. In contrast, the minimum “precipitation interception (%)” was noticed in soils labeled “NT.OC-1%.PDPG6” and “CT.OC-1%PDPG6,” with only 0.11 percent recorded in 2010. However, considering average values, soils labeled “NT.OC-7%PDPG8 & CT.OC-7%PDPG8” exhibited the highest precipitation interception (%) at 0.44 percent. Conversely, soils labeled “NT.OC-1%.PDPG6 & CT.OC-1%PDPG6” pointed to the lowest average precipitation interception (%) at 0.27. The findings are comparable with other studies (Leuning et al., 1994). Supplementary Figure S5 shows seasonal variation in precipitation interception.

### 3.2 Strengths and limitations of the BBN model of tilling wheat conflicting climatic and sustainability challenges

This BBN model assessed tilling systems under climate change and sustainability challenges for winter wheat crop production. The assessment compared 48 cropping seasons of simulations for each included variable response and assessed their long-term impacts on wheat yield, surface runoff, and GHG-CO2 emissions as output variables (Nielsen and Vigil, 2010; Krauss et al., 2017; Nath et al., 2017). This model gained insight into those parameter responses where empirical data is not readily available except for soil, weather, wheat management and yield data. However, the synthetic datasets were generated using established machine-learning models in DSSAT. Then, the BBN model utilized a crop growth hierarchal model DSSAT output data from its prototype model experiment. The BBN model offers integration of identified variables using standardized responses to capture uncertainties among model parameters. These responses reflect conditional dependencies and are consistent with the established knowledge in the domain.

It is important to understand BBN, which provides a graphical probabilistic model representing a set of variables and their probabilistic dependencies using a directed acyclic graph. It modeled uncertain relationships between variables and made probabilistic inferences based on observed evidence. Contrarily, an emulator is a simplified mathematical model that approximates the behavior of a more complex, computationally expensive model. Emulators are typically used to speed up the simulation process by quickly estimating model outputs based on a subset of input values and mimicking the source (Aakula et al., 2024). However, the DSSAT is a mechanistic crop growth hierarchical model and serves as a simulator in this study. This tool operated based on its embedded machine learning capabilities and provided the DSSAT output data used as an input in this BBN. This BBN model presents probabilistic inferences and reasoning, serves different purposes, operates on different assumptions. However, this approach may have the potential to emulate functions not evaluated in this study (Liu and West, 2009). BBN models could have other challenges in building and maintaining large complex networks, and computational expansive, especially data-driven networks are highly dependent on the amount and quality of data. The BBN models can not represent feedback loops or cyclic dependencies between variables which could challenge applicability to certain domains. In such cases, alternative probabilistic models such as hidden Markov models or Markov random fields might be a better substitute. Nonetheless, this BBN model did not come across the challenges above (Wang, 2004).

The BBN model is appraised per established metrics and summarized in Table 5. No contradictory responses were observed from model performance except for a higher error rate in predictive accuracy highlighted in the confusion matrix. The error rate is higher for GHG, runoff, and yield and is reported as 29.39, 33.64, and 43.94%, respectively. This outcome could be due to a greater variation in the organic carbon contents defined with varying fluctuations among 30 soils (Tibshirani and Tibshirani, 2009). Three real soils with built in soil organic level contents have been used to simulate each into four varying OC levels. Hence, the variation in GHG, runoff, and yield must appear in the results and are also subject to a higher error rate. However, the model’s predictive accuracy is still over 56% among all output variables of interest using the holdout method of model validation. The model was trained using data from 1975 to 2011 and tested against 2012–2022. However, the model was also validated using the K-Fold Cross Validation (CV) method using K-fold splits into training and test datasets. Then, the model was trained using data from 1975 to 2014 (K1 + K2 + K3 + K4) and tested using data from 2015 to 2022 (K5) to yield consistent and reliable results, proved (Table 4; Supplementary material S1). This challenge commonly appears among models in several agroecological studies, and an example is presented in Figure 5 by comparing published crop yield datasets (2016–2021) from research covering the same catchment area (Hazarika et al., 2009; Karimi et al., 2021; Mahmood et al., 2023). Model performance could be improved by employing parameter tuning, varying (scaling) parametric categories, changing (normalization) datasets, data homogeneousness, etc (Singh and Singh, 2022). Further exploration of the DSSAT tool for its interacting functionalities could be another area of interest for comparing model parameters’ performance against those of other competing tools.

The BBN model illustrated that the No-till (NT) choice of cultivation outclassed the Conventional till (CT) by reducing GHG-CO2 emissions probability estimation in the highest band from “7.34 to 7.31%.” Probabilistic estimates are expressed in percentages (e.g., 0 to 100%), representing the likelihood or probability of different outcomes based on the available evidence and model assumptions. They can be presented as ranges or uncertainty intervals, with one bound (lower or upper) specified (Hohle and Teigen, 2018). NT also exhibited a probability of a rising trend in higher wheat yield bands, increasing from “7.46 to 7.56%.” Cumulative runoff probability decreased in higher bands, with the “high band” diminishing from “8.52 to 8.50%.”

In contrast, the CT option had a snowballing trend in GHG-CO_2_ emissions in the highest band from “7.34 to 7.36%.” CT resulted in probabilistic estimates of lessening in wheat yield probability in the highest band from “7.46 to 7.35%,” while cumulative runoff amplified in higher bands, with a probability of the “high band” rising from “8.52 to 8.55%” (Radford, 2007; Lu et al., 2015). The impact of tillage methods on GHG-CO_2_ emission is linked with the potential phenomenon of creating soil disturbance in adapting tilling frequency and intensity, which can trigger disturbing soil aggregates, impacting water retention and moisture availability for plant uptake and influencing root development. These methods also influence soil microbial communities and their activities in decomposing soil organic matter. Nutrient cycling is another aspect affected by tilling choices, which can favor or resist underlying mechanisms. Tilling preference influences SOC contents. Higher levels of OC contents favor maintaining soil health conditions for building better soil structure and more resilient soils to help achieve better crop production.

The BBN model effectively apprehended uncertainties linked to seasonal variations by providing posterior probability data distributions reflecting probabilistic relationships across all variables and offered decision choice for NT favoring soil carbon stocks (highest among soil “NT.OC-7%PDPG8,” e.g., 286,634 kg/ha) in winter wheat. Furthermore, the model favors reduced GHG-CO_2_ emissions through NT practices and naturally restraining surface runoff generations for flood mitigation (Maraseni and Cockfield, 2011). For additional improvement of the BBN model, examination of early and late seasonal planting, supply rates of nutrients, and the use of various crop varieties under diverse farming systems are to be explored. A few constraints are not limited to access to empirical and synthetic datasets used in this model. Simulated datasets are used to calibrate and validate the BBN model (Aliferis and Cooper, 1994; Sebastiani and Ramoni, 2001; Newlands et al., 2014; Ali, 2023), so results variation could be expected where entire real/ empirical datasets will be applied.

This study focuses only on limited output variables, e.g., GHG-CO_2_ emissions and does not cater to nitrous (N_2_O) and methane (CH_4_) emissions. Surface runoff measurement presents only standard soil-specific features in the S-module of the DSSAT and could consider slope and topographic features for future exploration. The study utilized simulated wheat yield and compared that with real field data from the catchment area for 2016–2021. Utilizing catchment-wise yield data could enhance yield estimations on a larger scale. While simulated datasets were generated solely using the DSSAT tool, exploring other competing tools could yield comparable results. These areas offer futuristic research on this model. Moreover, variation in model response could also be expected due to spatio-temporal factors and the extent of noisy datasets once employed. Hence, perfect datasets can deliver more reliable model outcomes (Merino et al., 2016).

This research employed simulated datasets, offering valuable research, experimentation, and decision-making prospects. However, using simulated datasets entails implications regarding predictive accuracy, cost-effectiveness, experimental flexibility, risk mitigation, and ethical considerations. Researchers must carefully consider these factors when utilizing simulated data in their studies. Seeking real-world datasets whenever possible allows for comparing predicted results with observed data, mitigating some of these concerns. This study compared wheat yield datasets for comparing against simulated datasets and found few soils exhibited comparable results.

Overall, valuable improvements were made to the BBN, which would not have been possible without data access. This was particularly relevant in this study, where dataset access for a longer period was nonexistent. This approach helps integrate DSSAT simulated data into a BBN, provides an opportunity to measure against actual wheat yield (Figure 5) and provides a good insight to compare the model and identify the higher-performing soils under competing tilling methods. Hence, the model can potentially improve the accuracy, realism, and robustness of the model’s predictions and enable more informed decision-making in agricultural systems.

The current iteration of the BBN is in its alpha stage and recommends testing in diverse real-world scenarios to progress into beta versions. Successful scalability may lead to the development of a commercial gamma-level BBN model, customizable for diverse applications. Furthermore, enrichments in decision support functionalities, informed by real-life data testing, could enable the introduction of individual predictive models as independent software tools for mobile apps and portal-based interfaces.

The following are evident advantages achieved by this BBN model.

- The BBN model uniquely compared two different tilling systems, such as No-till (NT) and Conventional till (CT), magnifying the superiority of NT practices over CT.
- This BBN model effectively captured the uncertainties of key parameters responsible for winter wheat tilling systems toward climatic and sustainability challenges (Refer to Supplementary Tables S7, S8).
- The BBN model highlighted the dynamic nature of model variables and accurately reflected the responses of output variables based on the conditionality between interacting variables.
- This BBN model highlighted the role of C-SOM levels influenced by the competing tilling systems. This helped analyze the carbon sequestration impact between competitive tilling methods, e.g., NT methodology favors C-SOM over CT.
- The BBN model provided probabilistic precipitation retention/resilience estimates, comparing tilling techniques. This analysis can aid in assessing flood alleviation impacts, with the No-till approach showing a higher capacity for precipitation interception than Conventional tillage.
- The BBN model showed that adopting the No-till method reduced GHG-CO_2_ emissions compared to Conventional tillage practices.

The model can be used to inform decision-making and improve agricultural sustainability. For instance, adapting suitable tillage preferences is a crucial management choice with a commercial focus on obtaining crop yield. Conventional tillage choice could deliver instant yield gains at the cost of hampering long-term soil health and structural resilience. This approach will cater only to the interest of acquiring optimum crop yields at the cost of attracting several complicated issues of soil compaction erosion, consequently attracting higher surface runoff & greater flooding risk in the long run. For example, CT promotes frequent and intense tillage application, exerting mechanical pressure through interfacing soils. Heavy and frequent tillage can expose the soil organic matter to increased oxygen levels and microbial decomposition, affecting soil functioning for crop growth and triggering GHG-CO_2_ emissions. Tillage methods can also influence soil nutrient cycling processes by disturbing soil physical, chemical, and biological properties. Conventional tillage practices could accelerate the mineralization of organic matter, releasing nutrients and raising nutrient availability for plant uptake with an increased risk of loss of nutrients. These changes in nutrient availability affect crop growth, yield and nutrient use efficiency, which could influence soil carbon dynamics and GHG-CO_2_ emissions.

However, the reduced-to-no-till choice of cultivation can bring multifaceted benefits along with attaining sustainable crop yields over time, limiting the loss of SOC, preserving soil health conditions, limiting surface runoff due to land cover vegetation preference favoring flood alleviation, and reducing the risk of soil compaction and erosion (Farahani et al., 2022). At the helm of climate change and sustainability challenges, the importance of carbon sequestration has become manifold. This model supports reduced to no-till (NT) as the right choice of tillage practices through which crop residues and re can be retained on the land to get incorporated into soils, supporting land cover and increasing SOC contributions. This aligns with sustainable crop farming practices.

## 4 Conclusion

This paper presents a novel contribution by introducing a BBN model for no-tilling as an NFM strategy for wheat crop cultivation. This model presents valuable insight into the effects of tillage preferences on soil carbon dynamics, greenhouse gas emissions, and crop yield. The study promotes understanding how tilling choices influence the relationships between soil health, crop productivity, and ecological sustainability. This research identifies best tilling practices for management of SOC for sustainable crop yield while minimizing environmental impacts. Reduced to no-till practices promote offset of greenhouse gas emissions and encourage agroecosystem resilience. These research findings have implications for extending policy efforts for promoting sustainable soil management practices and provide scientific evidence of reduced to no-till practices’ benefits. These conclusions inform policy decisions, support extension efforts for the farming community, and incentivize the adoption of sustainable tilling practices.

This BBN model offers decision support among choosing alternative tilling systems to achieve sustainable wheat production by reducing surface runoff, and GHG-CO_2_ emissions by delivering probabilistic estimates of pertinent parameters. This BBN offers unique relationships between variables of multiple interests, such as seasonal wheat crop production, alleviation of floods and GHG-CO_2_ emissions. This BBN model provides an opportunity to cater to precipitation interception and soil water absorption to alleviate flooding risk in winter wheat cultivation regions while aiming for sustainable wheat production and soil carbon stocks. This BBN endorses the sustainable development goals defined by the FAO of the United Nations through promoting sustainable production and climate action while practicing sustainable farming.

This comparison illustrates the impacts of No-till (NT) and conventional till (CT) cultivation methods on three real soils from the Pang catchment area, each modeled with varying soil organic carbon (SOC) content in topsoil at four levels (1, 3, 5, 7%). Synthetic datasets were produced using the DSSAT tool over 48 years to evaluate long-term impacts on wheat crop yield, surface runoff, and GHG-CO_2_ emissions. DSSAT output files containing variable responses were accessed, reflecting embedded crop growth models. DSSAT, with multiple interfaces, served as a data simulator for this study and provided variable responses for soil attributes, weather, and crop management practices in the Pang catchment area. Contrarily, Netica software established a single interface for a BBN model, comparing tillage preference to wheat yield, surface runoff, and GHG-CO_2_ emissions. The pertinent variables’ data were extracted from DSSAT simulation compilations and formatted into a specified case file for BBN parameterization. The rationale for combining DSSAT and BBN strengthens from their complementary strengths in agricultural modeling and uncertainty quantification. DSSAT presented a well-established agricultural simulation model that represented crop growth, soil processes, and management practices for comparable model outputs. By contrast, the BBNs are adept at handling uncertainty and integrating various sources of information to make probabilistic predictions.

A BBN model was developed to capture the uncertainty in data distributions and model the variables’ interactions as a single interface tool. This approach helped analyze the impacts on model outputs, e.g., crop yield, runoff, and emissions. Tillage methods indirectly affect model-output variables of crop yield, surface runoff and GHG-CO_2_ emissions through carbon sequestration. These effects are insightful by various mechanisms such as soil structural changes caused by conventional tilling methods where soil aggregates are broken down, increase soil compaction, and reduce soil porosity, and water infiltration rates. These changes affect water retention, root penetration, and nutrient availability, which influence soil carbon dynamics, microbial activity and crop growth. Microbial activity is another crucial mechanism in organic matter decomposition, nutrient cycling, and soil health. Tilling methods affect soil physical and chemical properties by disturbing microbial activities and community composition, such as moisture content level, pH, oxygen availability, microbial habitat disturbance, and substrate availability.

The study revealed that NT and CT do not directly impact runoff and product weight gains but rather influence through intermediate variables, as shown in Figure 1. However, these methods can indirectly influence output variables through the implications of carbon sequestration organic matter (C-SOM) by altering the rate of C-SOM reduction in the soil profile over time. NT soils exhibited lower rates of C-SOM reduction than CT soils, indicating that the NT method enhances resilience toward C-SOM contents through multiple pathways. Higher C-SOM levels in soils provided greater available water space, reducing runoff impact and contributing to higher biomass and product weight. The individual model parameter is categorized in ascending order, ranging from lower to higher bands. This study discovered from variable responses from their simulated dataset response over 48 years that probabilistic estimates for the NT option are better than CT because this reduced GHG-CO_2_ emissions in the highest band from “7.34 to 7.31%.” It exhibited a rising trend in higher bands of wheat yield, with the highest band rising from “7.46 to 7.56%.” The cumulative runoff also tends to reduce in higher bands, with the high band declining from “8.52 to 8.50%.” Contrarily, the CT option increased GHG-CO_2_ emissions in the highest band from “7.34 to 7.36%.” This option also reduced the wheat yield in the highest band from “7.46 to 7.35%,” with cumulative runoff trending to rise in higher bands with the “high band” declining from “8.52 to 8.55%”.

The research concludes that soils with higher OC contents have relatively higher GHG-CO_2_ emissions, but this trend decreases in soils under NT applications. Hence, promoting NT cultivation for soils with higher OC contents can help reduce GHG-CO_2_ emissions, while the same applies to soils with lower OC contents to sustain emissions at reduced levels. Compared with CT, NT cultivation supports precipitation interception through biomass land cover, reducing the direct impact of rainfall on bare soils and minimizing flood risks. Additionally, higher crop biomass supports sustainable wheat crop yield.

The BBN model effectively captured uncertainties, offering posterior probability distributions reflecting conditional relationships across variables and offered decision choice for NT favoring soil carbon stocks in winter wheat (highest among soils “NT.OC-7%PDPG8,” e.g., 286,634 kg/ha) over CT (lowest in “CT.OC-3.9%PDPG8,” e.g., 5,894 kg/ha). On average, NT reduced minimum GHG- CO_2_ emissions to “3,985 kgCO_2_eqv/ha,” while CT emitted “7,415 kgCO_2_eqv/ha.” Conversely, NT emitted “8,747 kg CO_2_eqv/ha” for maximum emissions, while CT emitted “15,356 kg CO_2_eqv/ha.” This model represents probabilistic inferences based on specified datasets from the Pang catchment area. The model is recommended for testing by farmers, growers, landowners, or other stakeholders if they have datasets for all the variables used in this BBN. They may need to tune up the parametric adjustment for respective variables (nodes) in the model to incorporate the full range of data specified into appropriate categories or bin sizes to avoid loss of information. Moreover, the model can be customized based on farm-specific data and variables of interest.

However, improvements to the BBN model could involve considering early or late seasonal planting, diverse plant seedlings and nutrient supply rates, different farming systems, and various cultivars. There is also potential for future exploration to address multiple cropping, land fallowing, and crop rotation aspects. The BBN model could face potential challenges linked to long-term real-world datasets and computational resource availability. The research points out that the implications depicted from the model findings are confined solely to this study and were derived through specified data-driven exploration. Factors such as spatiotemporal variability, the availability of high-quality datasets, computational resources, and the diversity of soil conditions and farming systems may limit the accomplishment of comparable results across different situations. Moreover, this model could be investigated further for the next step to develop into a user-experience software application package and the model could also be tested for diverse spatiotemporal conditions for comparable variable responses.

Conclusively, reduced to no-tillage proved better than conventional tillage because the former encourages retention, maintenance, and storing of carbon stocks in soil and lessens GHG emissions. The model is a decision support tool that can be customized. For further improvement of the BBN model, exploration of early and late seasonal planting, nutrient supply rates and use of different cultivars under diverse farming systems are recommended. The research points out that the implications depicted from the model findings are confined solely to this study and were derived through specified data-driven exploration. Factors such as spatiotemporal variability, the availability of high-quality datasets, computational resources, and the diversity of soil conditions and farming systems may limit the accomplishment of comparable results across different situations.

However, research findings align with relevant sustainable development goals (SDGs), e.g., SDG12 and SDG13 for responsible production and climate actions defined by the Agriculture and Food Organization of the United Nations. SDG12 defines responsible consumption & production, and this research study promotes reduced or no-tillage practices as responsible production techniques that promote minimized soil disturbance, soil health, and limiting farm mechanization involving fuel and resource depletions. These research findings also encourage responsible use of resources, maintaining soil carbon stocks through tillage promoting carbon sequestration. SDG13 defines climate action, and this research study emphasizes adopting reduced or no-till practices as recommendations for contributing to climate action.

## Data availability statement

The original contributions presented in the study are included in the article/Supplementary material, further inquiries can be directed to the corresponding author.

## Author contributions

QA: Conceptualization, Data curation, Formal analysis, Investigation, Methodology, Validation, Visualization, Writing – original draft, Writing – review & editing.
