# Supplementary material for: Bayesian model of tilling wheat confronting climatic and sustainability challenges
Source: Front Artif Intell. 2024 Aug 27;7:1402098. doi: 10.3389/frai.2024.1402098 (PMC11385300; doi:10.3389/frai.2024.1402098)
Supplement: Supplementary file 1 [file Presentation_1.zip › supplementary material/Supplementary_Material.docx]

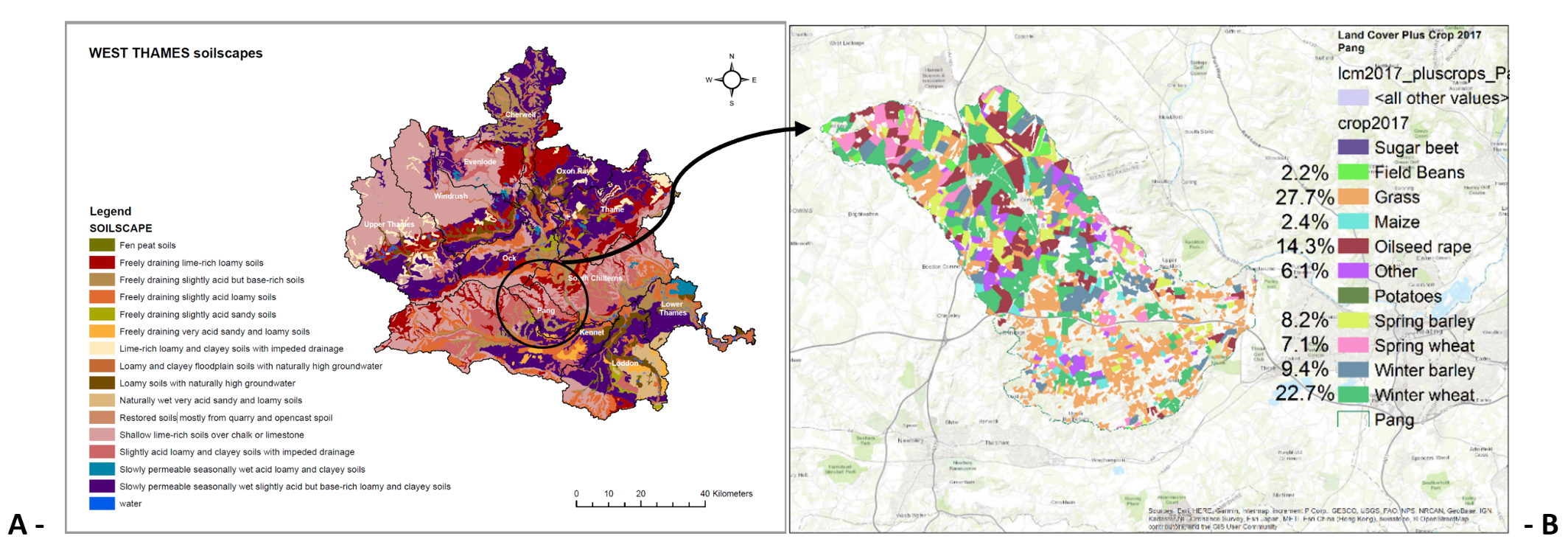
Supplementary Material (Maps, Figures and Tables)

#

**Fig S1 | Maps showing soilscape (A), land cover plus crop (B) in the Pang catchment [48, 49, 50]. Source: Data acquisition achieved through the project partner, *e.g.*, the NFM Landwise from NATMAP** [**LandIS - Land Information System - National Soil Map - NATMAP**](file:///C:\Users\qaisa\Desktop\P3%20-%20Simulation%20Modelling%20Practice%20and%20Theory\P3%20-%20Simulation%20Modelling%20Practice%20and%20Theory%20(1st%20submission)%20-%2022May24\supplementary%20material\LandIS%20-%20Land%20Information%20System%20-%20National%20Soil%20Map%20-%20NATMAP) **(https://www.landis.org.uk/data/natmap.cfm)**

**Fig S2 | Seasonal change in C-SOM (Kg/ha) in 30 soils under No-till versus Conventional till using simulated data from DSSAT v4.8 since 1975-2022.**

**Statistical analysis of C-SOM (Kg/ha) distribution in 30 soils under No-till and Conventional tillage - See Fig. S2.**

| **Statistical description** | **NT.OC-2.6%.PDPG6** | **NT.OC-1%.PDPG6** | **NT.OC-3%.PDPG6** | **NT.OC-5%PDPG6** | **NT.OC-7%PDPG6** | **CT.OC-2.6%PDPG6** | **CT.OC-1%PDPG6** | **CT.OC-3%PDPG6** | **CT.OC-5%PDPG6** | **CT.OC-7%PDPG6** |
| --- | --- | --- | --- | --- | --- | --- | --- | --- | --- | --- |
| **minimum** | -10 | -43 | 12 | 177 | 336 | -14 | -45 | 8 | 170 | 335 |
| **maximum** | 74 | 106 | 103 | 342 | 622 | 66 | 93 | 96 | 340 | 624 |
| **average** | 25.77 | 39.19 | 48.85 | 229.92 | 422.81 | 19.65 | 31.35 | 42.90 | 224.31 | 420.21 |
| **standard deviation** | 20.31 | 29.74 | 21.20 | 40.42 | 69.70 | 18.92 | 27.41 | 19.92 | 40.39 | 69.88 |
|  |  |  |  |  |  |  |  |  |  |  |
| **Statistical description** | **NT.OC-4.4%.PDPG7** | **NT.OC-1%.PDPG7** | **NT.OC-3%PDPG7** | **NT.OC-5%PDPG7** | **NT.OC-7%PDPG7** | **CT.OC-4.4%PDPG7** | **CT.OC-1%PDPG7** | **CT.OC-3%PDPG7** | **CT.OC-5%PDPG7** | **CT.OC-7%PDPG7** |
| **minimum** | 71 | -200 | -23 | 110 | 224 | 70 | 70 | -25 | 112 | 229 |
| **maximum** | 219 | -101 | 65 | 285 | 514 | 216 | 216 | 61 | 286 | 517 |
| **average** | 113.56 | -150.67 | 9.90 | 159.29 | 319.33 | 112.15 | 112.15 | 8.46 | 159.42 | 320.69 |
| **standard deviation** | 35.59 | 23.53 | 19.39 | 42.93 | 68.77 | 34.35 | 34.35 | 18.46 | 42.03 | 66.71 |
|  |  |  |  |  |  |  |  |  |  |  |
| **Statistical description** | **NT.OC-3.9%.PDPG8** | **NT.OC-1%.PDPG8** | **NT.OC-3%.PDPG8** | **NT.OC-5%PDPG8** | **NT.OC-7%PDPG8** | **CT.OC-3.9%PDPG8** | **CT.OC-1%PDPG8** | **CT.OC-3%PDPG8** | **CT.OC-5%PDPG8** | **CT.OC-7%PDPG8** |
| **minimum** | 195 | -174 | 91 | 295 | 513 | 194 | -175 | 89 | 299 | 518 |
| **maximum** | 351 | -88 | 200 | 500 | 860 | 347 | -89 | 196 | 499 | 862 |
| **average** | 243.63 | -129.40 | 129.17 | 356.79 | 635.52 | 241.40 | -130.63 | 127.54 | 357.56 | 637.42 |
| **standard deviation** | 38.35 | 20.35 | 25.33 | 52.11 | 84.44 | 37.33 | 20.51 | 24.36 | 50.71 | 82.35 |


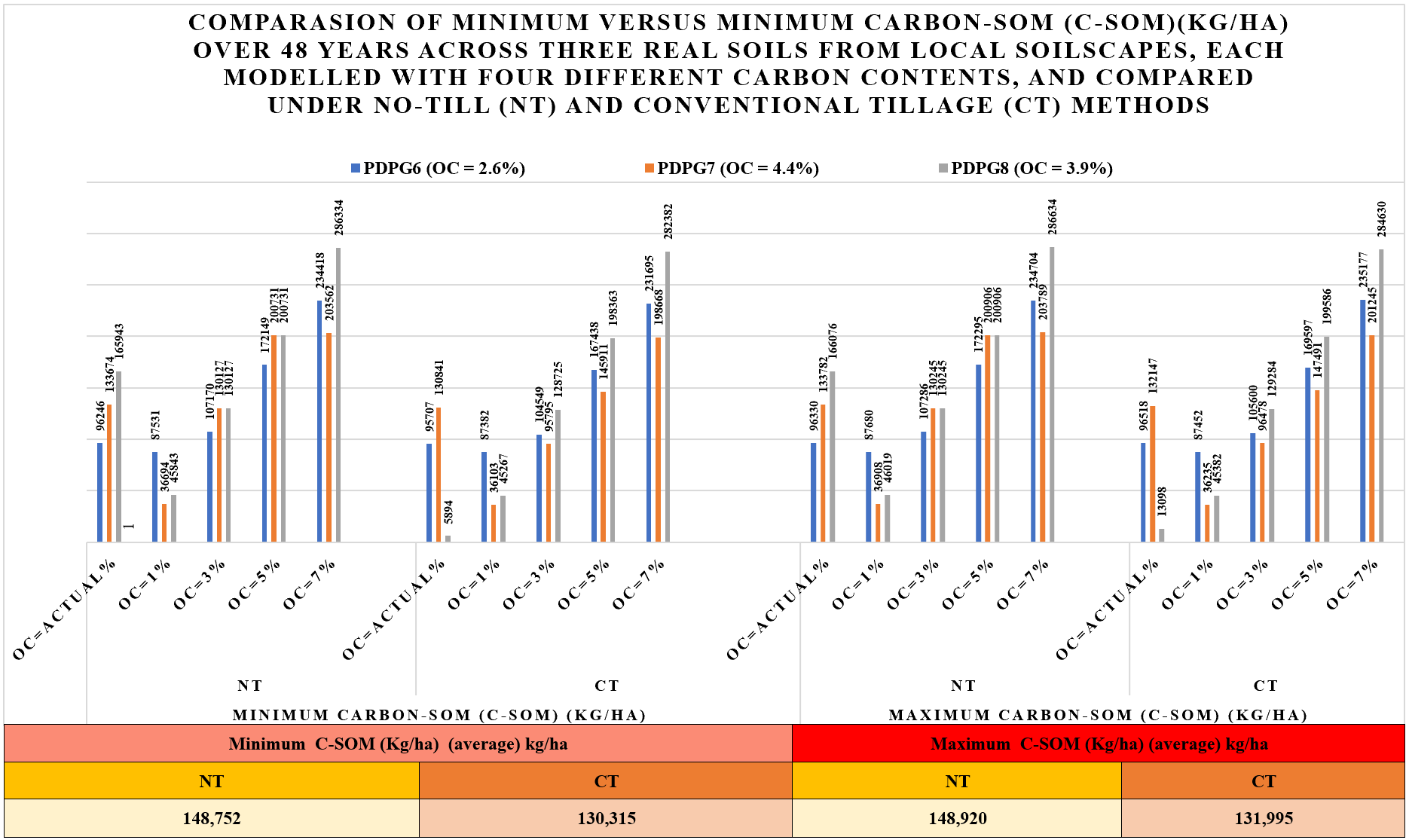


**Fig S3 | Comparison between minimum and maximum C-SOM (Kg/ha) levels in 30 soils under No-till versus Conventional till using simulated data from DSSAT v4.8 since 1975-2022.**

**Statistical analysis of the distribution of minimum and maximum C-SOM (Kg/ha) levels in 30 soils under No-till and Conventional tillage - See Fig. S3.**

| **Statistical description** | **NT.OC-2.6%.PDPG6** | **NT.OC-1%.PDPG6** | **NT.OC-3%.PDPG6** | **NT.OC-5%PDPG6** | **NT.OC-7%PDPG6** | **CT.OC-2.6%PDPG6** | **CT.OC-1%PDPG6** | **CT.OC-3%PDPG6** | **CT.OC-5%PDPG6** | **CT.OC-7%PDPG6** |
| --- | --- | --- | --- | --- | --- | --- | --- | --- | --- | --- |
| **minimum** | 96246 | 87531 | 107170 | 172149 | 234418 | 95707 | 87382 | 104549 | 167438 | 231695 |
| **maximum** | 96330 | 87680 | 107286 | 172295 | 234704 | 96518 | 87452 | 105600 | 169597 | 235177 |
| **average** | 96294 | 87598 | 107226 | 172235 | 234617 | 96167 | 87417 | 105149 | 168691 | 233724 |
| **standard deviation** | 20 | 30 | 26 | 34 | 70 | 188 | 16 | 244 | 532 | 885 |
|  |  |  |  |  |  |  |  |  |  |  |
| **Statistical description** | **NT.OC-4.4%.PDPG7** | **NT.OC-1%.PDPG7** | **NT.OC-3%PDPG7** | **NT.OC-5%PDPG7** | **NT.OC-7%PDPG7** | **CT.OC-4.4%PDPG7** | **CT.OC-1%PDPG7** | **CT.OC-3%PDPG7** | **CT.OC-5%PDPG7** | **CT.OC-7%PDPG7** |
| **minimum** | 133674 | 36694 | 130127 | 200731 | 203562 | 130841 | 36103 | 95795 | 145911 | 198668 |
| **maximum** | 133782 | 36908 | 130245 | 200906 | 203789 | 132147 | 36235 | 96478 | 147491 | 201245 |
| **average** | 133730 | 36822 | 130177 | 200841 | 203719 | 131596 | 36181 | 96196 | 146834 | 200110 |
| **standard deviation** | 27 | 44 | 26 | 40 | 51 | 330 | 37 | 164 | 401 | 652 |
|  |  |  |  |  |  |  |  |  |  |  |
| **Statistical description** | **NT.OC-3.9%.PDPG8** | **NT.OC-1%.PDPG8** | **NT.OC-3%.PDPG8** | **NT.OC-5%PDPG8** | **NT.OC-7%PDPG8** | **CT.OC-3.9%PDPG8** | **CT.OC-1%PDPG8** | **CT.OC-3%PDPG8** | **CT.OC-5%PDPG8** | **CT.OC-7%PDPG8** |
| **minimum** | 165943 | 45843 | 130127 | 200731 | 286334 | 5894 | 45267 | 128725 | 198363 | 282382 |
| **maximum** | 166076 | 46019 | 130245 | 200906 | 286634 | 13098 | 45382 | 129284 | 199586 | 284630 |
| **average** | 166013 | 45942 | 130177 | 200841 | 286540 | 8388 | 45331 | 129025 | 199021 | 283548 |
| **standard deviation** | 30 | 38 | 26 | 40 | 73 | 1602 | 34 | 141 | 309 | 558 |

**Fig S4 | Seasonal variation in available water space (cm^3^ cm^3^) in 30 soils under No-till versus Conventional till using simulated data from DSSAT v4.8 since 1975-2022.**

**Statistical analysis of the distribution of available water space (cm^3^ cm^3^) in 30 soils under No-till and Conventional tillage - See Fig. S4.**

| **Statistical description** | **NT.OC-2.6%.PDPG6** | **NT.OC-1%.PDPG6** | **NT.OC-3%.PDPG6** | **NT.OC-5%PDPG6** | **NT.OC-7%PDPG6** | **CT.OC-2.6%PDPG6** | **CT.OC-1%PDPG6** | **CT.OC-3%PDPG6** | **CT.OC-5%PDPG6** | **CT.OC-7%PDPG6** |
| --- | --- | --- | --- | --- | --- | --- | --- | --- | --- | --- |
| **minimum** | 226.38 | 208.93 | 228.18 | 247.23 | 267.22 | 226.96 | 211.04 | 229.82 | 250.40 | 271.51 |
| **maximum** | 256.38 | 234.10 | 258.02 | 281.43 | 304.44 | 256.26 | 235.62 | 259.79 | 285.14 | 309.68 |
| **average** | 238.10 | 220.86 | 241.05 | 261.16 | 282.17 | 239.54 | 222.30 | 242.50 | 264.63 | 287.48 |
| **standard deviation** | 6.50 | 4.50 | 5.69 | 6.94 | 8.18 | 5.56 | 4.44 | 5.77 | 7.01 | 8.31 |
|  |  |  |  |  |  |  |  |  |  |  |
| **Statistical description** | **NT.OC-4.4%.PDPG7** | **NT.OC-1%.PDPG7** | **NT.OC-3%PDPG7** | **NT.OC-5%PDPG7** | **NT.OC-7%PDPG7** | **CT.OC-4.4%PDPG7** | **CT.OC-1%PDPG7** | **CT.OC-3%PDPG7** | **CT.OC-5%PDPG7** | **CT.OC-7%PDPG7** |
| **minimum** | 158.62 | 126.60 | 145.75 | 164.08 | 176.86 | 160.99 | 132.89 | 150.64 | 176.07 | 188.39 |
| **maximum** | 195.08 | 155.83 | 179.09 | 201.66 | 215.17 | 196.65 | 155.99 | 179.33 | 206.82 | 220.37 |
| **average** | 172.07 | 137.66 | 157.86 | 178.01 | 191.22 | 174.96 | 142.72 | 163.77 | 190.91 | 204.00 |
| **standard deviation** | 7.94 | 5.89 | 7.07 | 8.28 | 8.58 | 7.80 | 4.91 | 6.33 | 7.29 | 7.69 |
|  |  |  |  |  |  |  |  |  |  |  |
| **Statistical description** | **NT.OC-3.9%.PDPG8** | **NT.OC-1%.PDPG8** | **NT.OC-3%.PDPG8** | **NT.OC-5%PDPG8** | **NT.OC-7%PDPG8** | **CT.OC-3.9%PDPG8** | **CT.OC-1%PDPG8** | **CT.OC-3%PDPG8** | **CT.OC-5%PDPG8** | **CT.OC-7%PDPG8** |
| **minimum** | 438.78 | 400.15 | 427.09 | 451.41 | 469.91 | 448.00 | 408.28 | 434.01 | 465.87 | 486.61 |
| **maximum** | 485.25 | 440.67 | 471.87 | 498.51 | 520.06 | 486.00 | 440.79 | 472.21 | 505.36 | 527.81 |
| **average** | 457.45 | 417.14 | 445.20 | 471.23 | 491.94 | 466.04 | 423.87 | 452.60 | 486.18 | 507.51 |
| **standard deviation** | 10.90 | 8.64 | 10.24 | 11.46 | 12.14 | 9.74 | 7.57 | 9.32 | 9.90 | 10.62 |

**Fig S5 | Seasonal variation in precipitation interception (%) in 30 soils under No-till versus Conventional till using simulated data from DSSAT v4.8 since 1975-2022.**

**Statistical analysis of the distribution of precipitation interception (%) in 30 soils under No-till and Conventional tillage - See Fig. S5.**

| **Statistical description** | **NT.OC-2.6%.PDPG6** | **NT.OC-1%.PDPG6** | **NT.OC-3%.PDPG6** | **NT.OC-5%PDPG6** | **NT.OC-7%PDPG6** | **CT.OC-2.6%PDPG6** | **CT.OC-1%PDPG6** | **CT.OC-3%PDPG6** | **CT.OC-5%PDPG6** | **CT.OC-7%PDPG6** |
| --- | --- | --- | --- | --- | --- | --- | --- | --- | --- | --- |
| **minimum** | 0.15 | 0.11 | 0.16 | 0.21 | 0.23 | 0.15 | 0.11 | 0.16 | 0.22 | 0.23 |
| **maximum** | 0.50 | 0.47 | 0.51 | 0.54 | 0.57 | 0.51 | 0.47 | 0.51 | 0.55 | 0.57 |
| **average** | 0.31 | 0.27 | 0.32 | 0.36 | 0.40 | 0.31 | 0.27 | 0.32 | 0.37 | 0.40 |
| **standard deviation** | 0.08 | 0.09 | 0.08 | 0.08 | 0.07 | 0.08 | 0.09 | 0.08 | 0.08 | 0.07 |
|  |  |  |  |  |  |  |  |  |  |  |
| **Statistical description** | **NT.OC-4.4%.PDPG7** | **NT.OC-1%.PDPG7** | **NT.OC-3%PDPG7** | **NT.OC-5%PDPG7** | **NT.OC-7%PDPG7** | **CT.OC-4.4%PDPG7** | **CT.OC-1%PDPG7** | **CT.OC-3%PDPG7** | **CT.OC-5%PDPG7** | **CT.OC-7%PDPG7** |
| **minimum** | 0.23 | 0.18 | 0.22 | 0.23 | 0.24 | 0.23 | 0.23 | 0.22 | 0.23 | 0.24 |
| **maximum** | 0.55 | 0.49 | 0.53 | 0.56 | 0.57 | 0.55 | 0.55 | 0.53 | 0.56 | 0.56 |
| **average** | 0.39 | 0.31 | 0.36 | 0.40 | 0.41 | 0.39 | 0.39 | 0.36 | 0.40 | 0.41 |
| **standard deviation** | 0.07 | 0.08 | 0.07 | 0.07 | 0.07 | 0.07 | 0.07 | 0.07 | 0.06 | 0.06 |
|  |  |  |  |  |  |  |  |  |  |  |
| **Statistical description** | **NT.OC-3.9%.PDPG8** | **NT.OC-1%.PDPG8** | **NT.OC-3%.PDPG8** | **NT.OC-5%PDPG8** | **NT.OC-7%PDPG8** | **CT.OC-3.9%PDPG8** | **CT.OC-1%PDPG8** | **CT.OC-3%PDPG8** | **CT.OC-5%PDPG8** | **CT.OC-7%PDPG8** |
| **minimum** | 0.22 | 0.20 | 0.22 | 0.23 | 0.24 | 0.22 | 0.20 | 0.22 | 0.23 | 0.24 |
| **maximum** | 0.58 | 0.53 | 0.57 | 0.58 | 0.59 | 0.58 | 0.53 | 0.57 | 0.58 | 0.59 |
| **average** | 0.42 | 0.37 | 0.41 | 0.43 | 0.44 | 0.42 | 0.37 | 0.41 | 0.43 | 0.44 |
| **standard deviation** | 0.07 | 0.07 | 0.07 | 0.06 | 0.06 | 0.07 | 0.07 | 0.07 | 0.06 | 0.06 |

**Table S1 | Data description of three soilscapes (soil features, characteristics, classifications, textural composition) from the Pang catchment.**

| ****Soil-scape*** | *****SSEW higher categories*** | ***Landwise Aggregated Soilscapes*** | ******RB209*** | ***Think Soils*** | ***Generalized Geology*** | ***Top layer***  ***up to 10-15 cm*** | |
| --- | --- | --- | --- | --- | --- | --- | --- |
|  |  |  |  |  |  | ***OC (%)*** | ***Texture (%)*** |
| ***PDPG6*** | *Brown* | *Loam:*  *Free drainage loamy* | *Medium soils* | *Medium soils/ Chalk, limestone soils* | *Carbonate* | ***2.6*** | ***Clay= 13***  ***Silt= 29***  ***Rock= 12.7*** |
| ***PDPG7*** | *Brown* | *Loam:*  *Free drainage loamy* | *Medium soils* | *Medium soils/ Chalk, limestone soils* | *Carbonate* | ***4.4*** | ***Clay= 23***  ***Silt= 42***  ***Rock= 13.8*** |
| ***PDPG8*** | *Pelosol/ Argillic brown earth* | *Clay over chalk:*  *Impeded drainage loamy/ clayey* | *Medium soils*  *Or Deep clays/ deep silty soils* | *Medium soils* | *Carbonate* | ***3.9*** | ***Clay= 21***  ***Silt= 47***  ***Rock= 14*** |

*Soilscape ID = PDPG6, PDPG7, PDPG8 **SSEW = Soil survey of England and Wales. ***RB = Nutrients management guide reference book.

Three soils (PDPG6, PDPG7, and PDPG8) and their attributes from the NETMAP soilscapes data were acquired from the NFM Landwise Project partner. These soils were labelled to correspond with their original identification within the soilscape. It's important to distinguish between the soil attributes listed in the tables and soilscape terminology. This study uses soilscape terminology interchangeably with soils to prevent technical confusion. The variable labelled 'soilscape' in the BBN model represents soils in this research.

| ***PDPG6** | **Depth (cm)** | **Soil Organic Carbon (% Soil Weight)** | **Clay % (<0.002mm)** | **Silt % (b/w 0.05 to 0.002mm)** | **Rock % (> 2mm)** | **Soil Bulk Density (Mg/m**^3^**)** | **Sat. Hydraulic Conductivity (cm/hr) DSSAT** | **Sat. Hydraulic Conductivity (cm/hr)** ****Rosetta** | **Lower limit (SLLL)** | **Drained upper limit (SDUL)** | **Saturated water content (SSAT)** |
| --- | --- | --- | --- | --- | --- | --- | --- | --- | --- | --- | --- |
| **PDPG**  **260006**  **OC=2.6%** | 10 | **2.6** | 13 | 29 | 12.7 | 1.15 | **2.59** | 3.1 | 0.141 | 0.281 | 0.436 |
|  | 50 | 0.7 | 14 | 16 | 12.8 | 1.39 | **2.59** | 3.0 | 0.1 | 0.178 | 0.354 |
|  | 70 | 0.3 | 23 | 13 | 12.4 | 1.44 | **0.43** | 2.1 | 0.131 | 0.2 | 0.337 |
|  | 150 | 0.1 | 16 | 5 | 15.2 | 1.49 | **2.59** | 4.8 | 0.092 | 0.138 | 0.294 |
| ***PDPG 700006** | **Depth (cm)** | **Soil Organic Carbon (% Soil Weight)** | **Clay % (<0.002mm)** | **Silt % (b/w 0.05 to 0.002mm)** | **Rock % (> 2mm)** | **Soil Bulk Density (Mg/m**^3^**)** | **Sat. Hydraulic Conductivity (cm/hr) DSSAT** | **Sat. Hydraulic Conductivity (cm/hr)** ****Rosetta** | **Lower limit (SLLL)** | **Drained upper limit (SDUL)** | **Saturated water content (SSAT)** |
| **PDPG**  **700006**  **OC=7%** | 10 | **7** | 13 | 29 | 12.73 | 1.00 | 2.59 | 3.1 | 0.246 | 0.480 | 0.521 |
|  | 50 | 1.9 | 14 | 16 | 12.83 | 1.34 | 2.59 | 3.3 | 0.129 | 0.232 | 0.397 |
|  | 70 | 0.8 | 23 | 13 | 12.39 | 1.49 | 0.43 | 2.2 | 0.143 | 0.222 | 0.359 |
|  | 150 | 0.3 | 16 | 5 | 15.22 | 1.64 | 2.59 | 4.9 | 0.097 | 0.146 | 0.305 |
| ***PDPG 500006** | **Depth (cm)** | **Soil Organic Carbon (% Soil Weight)** | **Clay % (<0.002mm)** | **Silt % (b/w 0.05 to 0.002mm)** | **Rock % (> 2mm)** | **Soil Bulk Density (Mg/m**^3^**)** | **Sat. Hydraulic Conductivity (cm/hr) DSSAT** | **Sat. Hydraulic Conductivity (cm/hr)** ****Rosetta** | **Lower limit (SLLL)** | **Drained upper limit (SDUL)** | **Saturated water content (SSAT)** |
| **PDPG**  **500006**  **OC=5%** | 10 | **5** | 13 | 29 | 12.73 | 1.01 | 2.59 | 4.1 | 0.198 | 0.389 | 0.489 |
|  | 50 | 1.3 | 14 | 16 | 12.83 | 1.41 | 2.59 | 3.2 | 0.115 | 0.205 | 0.379 |
|  | 70 | 0.6 | 23 | 13 | 12.39 | 1.52 | 0.43 | 2.1 | 0.138 | 0.213 | 0.350 |
|  | 150 | 0.2 | 16 | 5 | 15.22 | 1.66 | 2.59 | 4.9 | 0.095 | 0.142 | 0.299 |
| ***PDPG 300006** | **Depth (cm)** | **Soil Organic Carbon (% Soil Weight)** | **Clay % (<0.002mm)** | **Silt % (b/w 0.05 to 0.002mm)** | **Rock % (> 2mm)** | **Soil Bulk Density (Mg/m**^3^**)** | **Sat. Hydraulic Conductivity (cm/hr) DSSAT** | **Sat. Hydraulic Conductivity (cm/hr)** ****Rosetta** | **Lower limit (SLLL)** | **Drained upper limit (SDUL)** | **Saturated water content (SSAT)** |
| **PDPG**  **300006**  **OC=3%** | 10 | **3** | 13 | 29 | 12.73 | 1.17 | 2.59 | 3.3 | 0.151 | 0.299 | 0.446 |
|  | 50 | 0.8 | 14 | 16 | 12.83 | 1.48 | 2.59 | 3.1 | 0.103 | 0.182 | 0.360 |
|  | 70 | 0.3 | 23 | 13 | 12.39 | 1.57 | 0.43 | 2.1 | 0.131 | 0.200 | 0.337 |
|  | 150 | 0.1 | 16 | 5 | 15.22 | 1.68 | 2.59 | -99** | 0.092 | 0.138 | 0.294 |
| ***PDPG 100006** | **Depth (cm)** | **Soil Organic Carbon (% Soil Weight)** | **Clay % (<0.002mm)** | **Silt % (b/w 0.05 to 0.002mm)** | **Rock % (> 2mm)** | **Soil Bulk Density (Mg/m**^3^**)** | **Sat. Hydraulic Conductivity (cm/hr) DSSAT** | **Sat. Hydraulic Conductivity (cm/hr)** ****Rosetta** | **Lower limit (SLLL)** | **Drained upper limit (SDUL)** | **Saturated water content (SSAT)** |
| **PDPG**  **100006**  **OC=1%** | 10 | **1** | 13 | 29 | 12.73 | 1.38 | 2.59 | 2.4 | 0.103 | 0.209 | 0.391 |
|  | 50 | 0.3 | 14 | 16 | 12.83 | 1.56 | 2.59 | 3.0 | 0.091 | 0.160 | 0.338 |
|  | 70 | 0.1 | 23 | 13 | 12.39 | 1.60 | 0.43 | 2.1 | 0.126 | 0.191 | 0.329 |
|  | 150 | 0.0 | 16 | 5 | 15.22 | 1.69 | 2.59 | 5.2 | 0.090 | 0.133 | 0.292 |

**Table S2 | Soilscape PDPG6 shows real soil in orange and modelled soils in pink.**

*PDPG6 represents soilscape; its real soil name is coded as PDPG260006 (the first two digits represent OC level of 2.6%, and the last digit 6 belongs to PDPG6). Four modelled soils with varying OC content at four levels: 1%, 3%, 5%, & 7% were developed. They were then coded with unique IDs starting with the first digit showing OC level percentage and ending digit indicating real soilscape ID such as PDPG100006 (first digit 1 means OC level 1% and last digit 6 fitting to PDPG6). Similarly, PDPG700006, PDPG500006, and PDPG300006 represent modelled soils adhering to the same principle, with organic carbon levels of 7%, 5%, and 3% respectively, aligning with the specifications of PDPG6.

**The Rosetta online tool (<https://soil-modeling.org/resources-links/model-portal/rosetta>) was also used to calculate saturated hydraulic conductivity values based on Soilscape data, such as texture concentrations. However, these values were not used in the modelling work because they were higher in the top soil layers and gave errors in model runs compared to the values derived from the DSSAT tool. Hence, the DSSAT tool was used for its reliability in delivering simulation results, as it incorporates pedotransfer functions in hierarchical crop growth modelling.

| ***PDPG7** | **Depth (cm)** | **Soil Organic Carbon (% Soil Weight)** | **Clay % (<0.002mm)** | **Silt % (b/w 0.05 to 0.002mm)** | **Rock % (> 2mm)** | **Soil Bulk Density (Mg/m**^3^**)** | **Sat. Hydraulic Conductivity (cm/hr)**  **DSSAT** | **Sat. Hydraulic Conductivity (cm/hr)**  ****Rosetta** | **Lower limit (SLLL)** | **Drained upper limit (SDUL)** | **Saturated water content (SSAT)** |
| --- | --- | --- | --- | --- | --- | --- | --- | --- | --- | --- | --- |
| **PDPG**  **440007**  **OC=4.4%** | 15 | **4.4** | 23 | 42 | 13.8 | 1.02 | **1.32** | 3.2 | 0.225 | 0.429 | 0.489 |
|  | 40 | 1.7 | 20 | 44 | 13.9 | 1.23 | **1.32** | 1.9 | 0.148 | 0.297 | 0.438 |
|  | 60 | 0.5 | 31 | 32 | 13.2 | 1.41 | **0.23** | 0.9 | 0.169 | 0.279 | 0.417 |
| ***PDPG 700007** | **Depth (cm)** | **Soil Organic Carbon (% Soil Weight)** | **Clay % (<0.002mm)** | **Silt % (b/w 0.05 to 0.002mm)** | **Rock % (> 2mm)** | **Soil Bulk Density (Mg/m**^3^**)** | **Sat. Hydraulic Conductivity (cm/hr)**  **DSSAT** | **Sat. Hydraulic Conductivity (cm/hr)**  ****Rosetta** | **Lower limit (SLLL)** | **Drained upper limit (SDUL)** | **Saturated water content (SSAT)** |
| **PDPG**  **700007**  **OC=7%** | 15 | **7** | 23 | 42 | 13.82 | 1.00 | 1.32 | 3.7 | 0.286 | 0.477 | 0.544 |
|  | 40 | 2.7 | 20 | 44 | 13.94 | 1.14 | 1.32 | 2.4 | 0.172 | 0.342 | 0.451 |
|  | 60 | 0.8 | 31 | 32 | 13.21 | 1.37 | 0.23 | 1.0 | 0.176 | 0.292 | 0.393 |
| ***PDPG 500007** | **Depth (cm)** | **Soil Organic Carbon (% Soil Weight)** | **Clay % (<0.002mm)** | **Silt % (b/w 0.05 to 0.002mm)** | **Rock % (> 2mm)** | **Soil Bulk Density (Mg/m**^3^**)** | **Sat. Hydraulic Conductivity (cm/hr)**  **DSSAT** | **Sat. Hydraulic Conductivity (cm/hr)**  ****Rosetta** | **Lower limit (SLLL)** | **Drained upper limit (SDUL)** | **Saturated water content (SSAT)** |
| **PDPG**  **500007**  **OC=5%** | 15 | **5** | 23 | 42 | 13.82 | 1.00 | 1.32 | 3.2 | 0.239 | 0.455 | 0.493 |
|  | 40 | 1.9 | 20 | 44 | 13.94 | 1.21 | 1.32 | 2.0 | 0.153 | 0.306 | 0.434 |
|  | 60 | 0.6 | 31 | 32 | 13.21 | 1.40 | 0.23 | 1.0 | 0.171 | 0.283 | 0.385 |
| ***PDPG 300007** | **Depth (cm)** | **Soil Organic Carbon (% Soil Weight)** | **Clay % (<0.002mm)** | **Silt % (b/w 0.05 to 0.002mm)** | **Rock % (> 2mm)** | **Soil Bulk Density (Mg/m**^3^**)** | **Sat. Hydraulic Conductivity (cm/hr)**  **DSSAT** | **Sat. Hydraulic Conductivity (cm/hr)**  ****Rosetta** | **Lower limit (SLLL)** | **Drained upper limit (SDUL)** | **Saturated water content (SSAT)** |
| **PDPG**  **300007**  **OC=3%** | 15 | **3** | 23 | 42 | 13.82 | 1.12 | 1.32 | 2.4 | 0.192 | 0.366 | 0.457 |
|  | 40 | 1.2 | 20 | 44 | 13.94 | 1.28 | 1.32 | 1.4 | 0.137 | 0.275 | 0.416 |
|  | 60 | 0.3 | 31 | 32 | 13.21 | 1.44 | 0.23 | 0.3 | 0.164 | 0.270 | 0.374 |
| ***PDPG 100007** | **Depth (cm)** | **Soil Organic Carbon (% Soil Weight)** | **Clay % (<0.002mm)** | **Silt % (b/w 0.05 to 0.002mm)** | **Rock % (> 2mm)** | **Soil Bulk Density (Mg/m**^3^**)** | **Sat. Hydraulic Conductivity (cm/hr)**  **DSSAT** | **Sat. Hydraulic Conductivity (cm/hr)**  ****Rosetta** | **Lower limit (SLLL)** | **Drained upper limit (SDUL)** | **Saturated water content (SSAT)** |
| **PDPG**  **100007**  **OC=1%** | 15 | **1** | 23 | 42 | 13.82 | 1.32 | 1.32 | 6.3 | 0.145 | 0.278 | 0.405 |
|  | 40 | 0.4 | 20 | 44 | 13.94 | 1.38 | 1.32 | 0.6 | 0.118 | 0.240 | 0.389 |
|  | 60 | 0.1 | 31 | 32 | 13.21 | 1.46 | 0.23 | 0.3 | 0.159 | 0.261 | 0.370 |

**Table S3 | Soilscape PDPG7 shows real soil in orange and modelled soils in pink.**

*PDPG7 represents soilscape; its real soil name is coded as PDPG440007 (the first two digits represent the OC level of 4.4%, and the last digit 7 belongs to PDPG7). Four modelled soils with varying OC content at four levels: 1%, 3%, 5%, & 7% were developed. They were then coded with unique IDs starting with the first digit showing OC level percentage and ending digit indicating real soilscape ID such as PDPG300007 (first digit 3 means OC level 3% and last digit 7 fitting to PDPG7). Similarly, PDPG700007, PDPG500007, and PDPG100007 represent modelled soils adhering to the same principle, with organic carbon levels of 7%, 5%, and 1% respectively, aligning with the specifications of PDPG6.

**The Rosetta online tool (<https://soil-modeling.org/resources-links/model-portal/rosetta>) was also used to calculate saturated hydraulic conductivity values based on soil attributes, such as soil textural composition and OC level. However, these values were not used in the modelling work because they were higher in the top soil layers and gave errors in model execution compared to the values derived by the S-module of the DSSAT tool itself. Hence, the soil files were generated using the S-module of the DSSAT tool and achieved its reliability in delivering simulation results, as it incorporates pedotransfer functions in hierarchical crop growth modelling for all the DSSAT outputs.

**Table S4 | Soilscape PDPG7 shows real soil in orange and modelled soils in pink.**

| ***PDPG8** | **Depth (cm)** | **SOC (% Soil Weight)** | **Clay % (<0.002mm)** | **Silt % (b/w 0.05 to 0.002mm)** | **Rock % (> 2mm)** | **Soil Bulk Density (Mg/m**^3^**)** | **Sat. Hydraulic Conductivity (cm/hr) DSSAT** | **Sat. Hydraulic Conductivity (cm/hr)** ****Rosetta** | **Lower limit (SLLL)** | **Drained upper limit (SDUL)** | **Saturated water content (SSAT)** |
| --- | --- | --- | --- | --- | --- | --- | --- | --- | --- | --- | --- |
| **PDPG**  **390008**  **OC=3.9%** | 15 | **3.9** | 21 | 47 | 14.22 | 1.03 | **1.32** | 3.3 | 0.204 | 0.403 | 0.479 |
|  | 35 | 1.2 | 20 | 49 | 14.02 | 1.25 | **1.32** | 1.8 | 0.136 | 0.282 | 0.425 |
|  | 55 | 0.7 | 29 | 40 | 16.46 | 1.34 | **0.23** | 1.1 | 0.159 | 0.281 | 0.388 |
|  | 105 | 0.5 | 48 | 28 | 18.40 | 1.34 | **0.06** | 1.1 | 0.228 | 0.333 | 0.38 |
|  | 150 | 0.4 | 42 | 41 | 19.64 | 1.31 | **0.09** | 1.0 | 0.199 | 0.318 | 0.384 |
| ***PDPG 700008** | **Depth (cm)** | **SOC (% Soil Weight)** | **Clay % (<0.002mm)** | **Silt % (b/w 0.05 to 0.002mm)** | **Rock % (> 2mm)** | **Soil Bulk Density (Mg/m**^3^**)** | **Sat. Hydraulic Conductivity (cm/hr) DSSAT** | **Sat. Hydraulic Conductivity (cm/hr)** ****Rosetta** | **Lower limit (SLLL)** | **Drained upper limit (SDUL)** | **Saturated water content (SSAT)** |
| **PDPG**  **700008**  **OC=7%** | 15 | **7** | 21 | 47 | 14.22 | 1 | 1.32 | 3.2 | 0.276 | 0.474 | 0.541 |
|  | 35 | 2.2 | 20 | 49 | 14.02 | 1.15 | 1.32 | 1.5 | 0.16 | 0.326 | 0.45 |
|  | 55 | 1.3 | 29 | 40 | 16.46 | 1.27 | 0.23 | 1.0 | 0.172 | 0.306 | 0.405 |
|  | 105 | 0.9 | 48 | 28 | 18.40 | 1.29 | 0.06 | 0.9 | 0.237 | 0.35 | 0.393 |
|  | 150 | 0.7 | 42 | 41 | 19.64 | 1.28 | 0.09 | 0.9 | 0.205 | 0.33 | 0.391 |
| ***PDPG 500008** | **Depth (cm)** | **SOC (% Soil Weight)** | **Clay % (<0.002mm)** | **Silt % (b/w 0.05 to 0.002mm)** | **Rock % (> 2mm)** | **Soil Bulk Density (Mg/m**^3^**)** | **Sat. Hydraulic Conductivity (cm/hr) DSSAT** | **Sat. Hydraulic Conductivity (cm/hr)** ****Rosetta** | **Lower limit (SLLL)** | **Drained upper limit (SDUL)** | **Saturated water content (SSAT)** |
| **PDPG**  **500008**  **OC=5%** | 15 | **5** | 21 | 47 | 14.22 | 1 | 1.32 | 2.8 | 0.229 | 0.452 | 0.497 |
|  | 35 | 1.5 | 20 | 49 | 14.02 | 1.21 | 1.32 | 1.7 | 0.144 | 0.297 | 0.435 |
|  | 55 | 0.9 | 29 | 40 | 16.46 | 1.31 | 0.23 | 1.1 | 0.163 | 0.289 | 0.396 |
|  | 105 | 0.6 | 48 | 28 | 18.40 | 1.33 | 0.06 | 1.0 | 0.230 | 0.337 | 0.383 |
|  | 150 | 0.5 | 42 | 41 | 19.64 | 1.3 | 0.09 | 1.2 | 0.201 | 0.322 | 0.386 |
| ***PDPG 300008** | **Depth (cm)** | **SOC (% Soil Weight)** | **Clay % (<0.002mm)** | **Silt % (b/w 0.05 to 0.002mm)** | **Rock % (> 2mm)** | **Soil Bulk Density (Mg/m**^3^**)** | **Sat. Hydraulic Conductivity (cm/hr) DSSAT** | **Sat. Hydraulic Conductivity (cm/hr)** ****Rosetta** | **Lower limit (SLLL)** | **Drained upper limit (SDUL)** | **Saturated water content (SSAT)** |
| **PDPG**  **300008**  **OC=3%** | 15 | **3** | 21 | 47 | 14.22 | 1.09 | 1.32 | 3.8 | 0.183 | 0.364 | 0.464 |
|  | 35 | 0.9 | 20 | 49 | 14.02 | 1.26 | 1.32 | 1.9 | 0.129 | 0.27 | 0.423 |
|  | 55 | 0.5 | 29 | 40 | 16.46 | 1.36 | 0.23 | 1.2 | 0.154 | 0.272 | 0.383 |
|  | 105 | 0.4 | 48 | 28 | 18.40 | 1.35 | 0.06 | 1.1 | 0.226 | 0.329 | 0.378 |
|  | 150 | 0.3 | 42 | 41 | 19.64 | 1.32 | 0.09 | 1.0 | 0.196 | 0.314 | 0.382 |
| ***PDPG 100008** | **Depth (cm)** | **SOC (% Soil Weight)** | **Clay % (<0.002mm)** | **Silt % (b/w 0.05 to 0.002mm)** | **Rock % (> 2mm)** | **Soil Bulk Density (Mg/m**^3^**)** | **Sat. Hydraulic Conductivity (cm/hr) DSSAT** | **Sat. Hydraulic Conductivity (cm/hr)** ****Rosetta** | **Lower limit (SLLL)** | **Drained upper limit (SDUL)** | **Saturated water content (SSAT)** |
| **DPG**  **100008**  **OC=1%** | 15 | **1** | 21 | 47 | 14.22 | 1.27 | 1.32 | 5.3 | 0.136 | 0.275 | 0.418 |
|  | 35 | 0.3 | 20 | 49 | 14.02 | 1.33 | 1.32 | 2.2 | 0.115 | 0.243 | 0.405 |
|  | 55 | 0.2 | 29 | 40 | 16.46 | 1.4 | 0.23 | 1.4 | 0.147 | 0.259 | 0.373 |
|  | 105 | 0.1 | 48 | 28 | 18.40 | 1.39 | 0.06 | 1.3 | 0.219 | 0.316 | 0.368 |
|  | 150 | 0.1 | 42 | 41 | 19.64 | 1.35 | 0.09 | 1.1 | 0.192 | 0.305 | 0.374 |

*PDPG8 represents soilscape; its real soil name is coded as PDPG390008 (the first two digits represent OC level of 3.9%, and the last digit 8 belongs to PDPG8). Four modelled soils with varying OC content at four levels: 1%, 3%, 5%, & 7% were developed. They were then coded with unique IDs starting with the first digit showing OC level percentage and ending digit indicating real soilscape ID such as PDPG300008 (first digit 3 means OC level 3% and last digit 8 fitting to PDPG8). Similarly, PDPG700008, PDPG500008, and PDPG100008 represent modelled soils adhering to the same principle, with organic carbon levels of 7%, 5%, and 1% respectively, aligning with the specifications of PDPG6.

**The Rosetta online tool (<https://soil-modeling.org/resources-links/model-portal/rosetta>) was also used to calculate saturated hydraulic conductivity values based on Soilscape data, such as texture concentrations. However, these values were not used in the modelling work because they were higher in the top soil layers and gave errors in model runs compared to the values derived from the DSSAT tool. Hence, the DSSAT tool was used for its reliability in delivering simulation results, as it incorporates pedotransfer functions in hierarchical crop growth modelling.

**Keynotes:**

- **Soil data description of soilscapes of the Pang catchment**

Three real soils from netmap data of the local soilscape were acquired and given names as PDPG6, PDPG7, and PDPG8. These are then coded for the real soils to their original soilscape identification. This study explored real soil data attributes such as soil zone (depth in cm), soil organic carbon (content in %), textural composition(clay, silt, rock in %age), soil bulk density (Mg/m^3^), saturated hydraulic conductivity (cm/hr), lower drainage limit (SLLL - cm^3^ cm^-3^), upper drainage limit (SDUL - cm^3^ cm^-3^) and saturated water content (SSAT - cm^3^ cm^-3^) and are summarized in Table S2, S3, and S4, respectively.

However, the following paragraph contains further details on that.

For instance, the study employed real soil (PDPG6) data attributes of soil zones/layers up to the depth of 10cm, 50cm, 70cm and 150cm with real soil organic content of 2.6%, 0.7%, 0.3% and 0.1% and real soil bulk density of 1.15(Mg/m^3^), 1.39(Mg/m^3^), 1.44(Mg/m^3^), 1.49(Mg/m^3^), respectively. Soil data also included real textural composition for clay(%), silt(%), and rocks(%) and pedotransfer functional values for each soil zone/ depth and, given in the top section (in orange colour) of Table S2. Similar details of soil data for PDPG7 & PDPG8 can be found in respective sections of the supplementary material in Table S3 & S4 respectively.

- **The mechanism for synthesis of soil OC level variations in the modelled soil**

Soil organic carbon variation was introduced in all three real soils with soil organic carbon contents (OC) variability of 1%, 3%, 5%, and 7%, respectively. These variations were implemented to compare performance outcomes between modelled soils and real soil, starting from the lowest OC level of 1% with an incremental increase of 2% to the highest OC level of 7%. These OC levels variability were considered for their potential deviations across catchment soils for diverse spatiotemporal factors, nature, landuse type and management. This aspect attracts the soil's comparative performance to cater to the desired potential for containing, maintaining, or increasing OC level whilst adapting a specific tillage strategy. The mechanism is expressed through the example below.

To model soil with varying OC levels, the real OC level was replaced with a new OC level and calculated its adjusted value throughout the soil zones/ depths. For instance, PDPG6 contained an original OC level of 2.6% up to 10 cm, 0.7% up to 50 cm, 0.3% up to 70 cm, and 0.1% up to 150 cm. In modelling PDPG6 with OC level of 7%. the OC level of 7% was introduced in the top layer up to 10 cm and 1.9% up to 50 cm (this was calculated based on the proportional % difference between the first-upper to the subsequent second-lower layer of the real soil where the first layer up to 10 cm of real soil contains 2.6% of OC levels and second layer up to 50 cm contains 0.7% which means OC level in the second layer is reduced by 0.7/2.6*100=26.92%. Hence, this proportional reduction of 26.92% was applied in the modelled soil of PDPG6 with 7% OC level for the first two layers, which means the top-**first layer up to 10 cm contained 7% OC level** and the subsequent **second layer up to 50 cm contained 1.9%** which is decreased by 26.92%.

Similarly, where the third layer up to 70 cm depth contained o.3% OC level in real soil of PDPG6 with real 2.6% OC level, 0.3% is a decreased level from its upper layer from 0.7% with a proportion of 42.85%. Hence, the third layer of the modelled soil of PDPG6 with 7% was applied with 42.85% of proportional reduction in 1.9% of OC level at up to the depth of 50 cm to reduce to have **0.8% up to the depth of 70 cm** in the subsequent lower layer. In the last layer of modelled soil of PDPG6 with OC level of 7%, **the OC level is 0.3% up to the depth of 150 cm**, which is a 33.33% reduction from its upper third layer OC level of 0.8%, which is following the proportional decrease in the real soil of PDPG6 third layer up to 70 cm containing 0.3 OC and reduces to 0.1 % in fourth layer with depth up to 150% *e.g.*, 0.1/0.3*100= 33.33%.

| **PDPG260006**  **(real soil - PDPG6 with 2.6%OC)** | | **PDPG700006**  **(modelled soil – PDPG6 with 7%OC)** | |
| --- | --- | --- | --- |
| **Depth (bottom), cm** | **Organic carbon, %**  **(% drop from upper layer)** | **Depth (bottom), cm** | **Organic carbon, %**  **(% drop from upper layer)** |
| **10** | ***2.6%*** | **10** | ***7%*** |
| **50** | **0.7 (-26.92%)** | **50** | **1.9 (-26.92%)** |
| **70** | **0.3 (-42.85%)** | **70** | **0.8 (-42.85%)** |
| **150** | **0.1 (-33.33%)** | **150** | **0.3 (-33.33%)** |


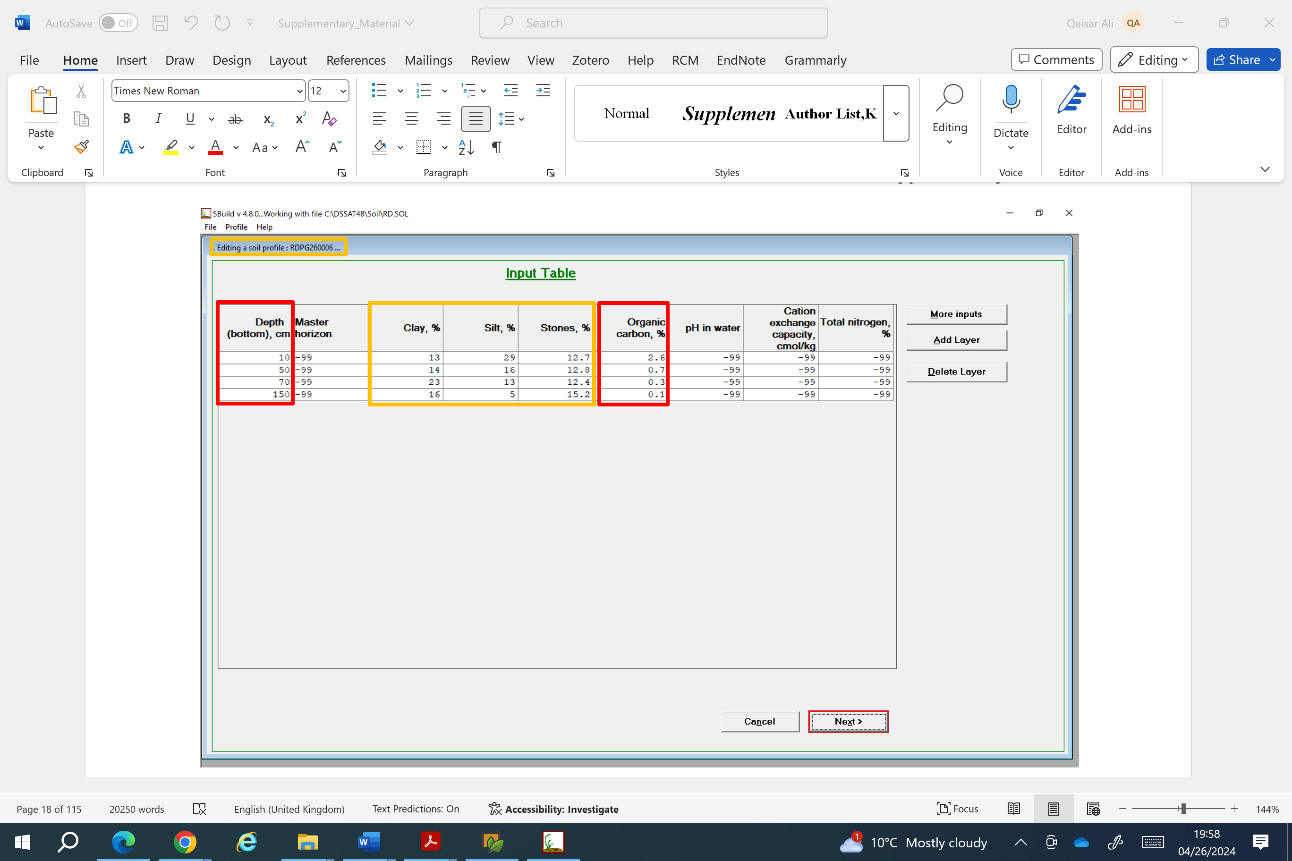

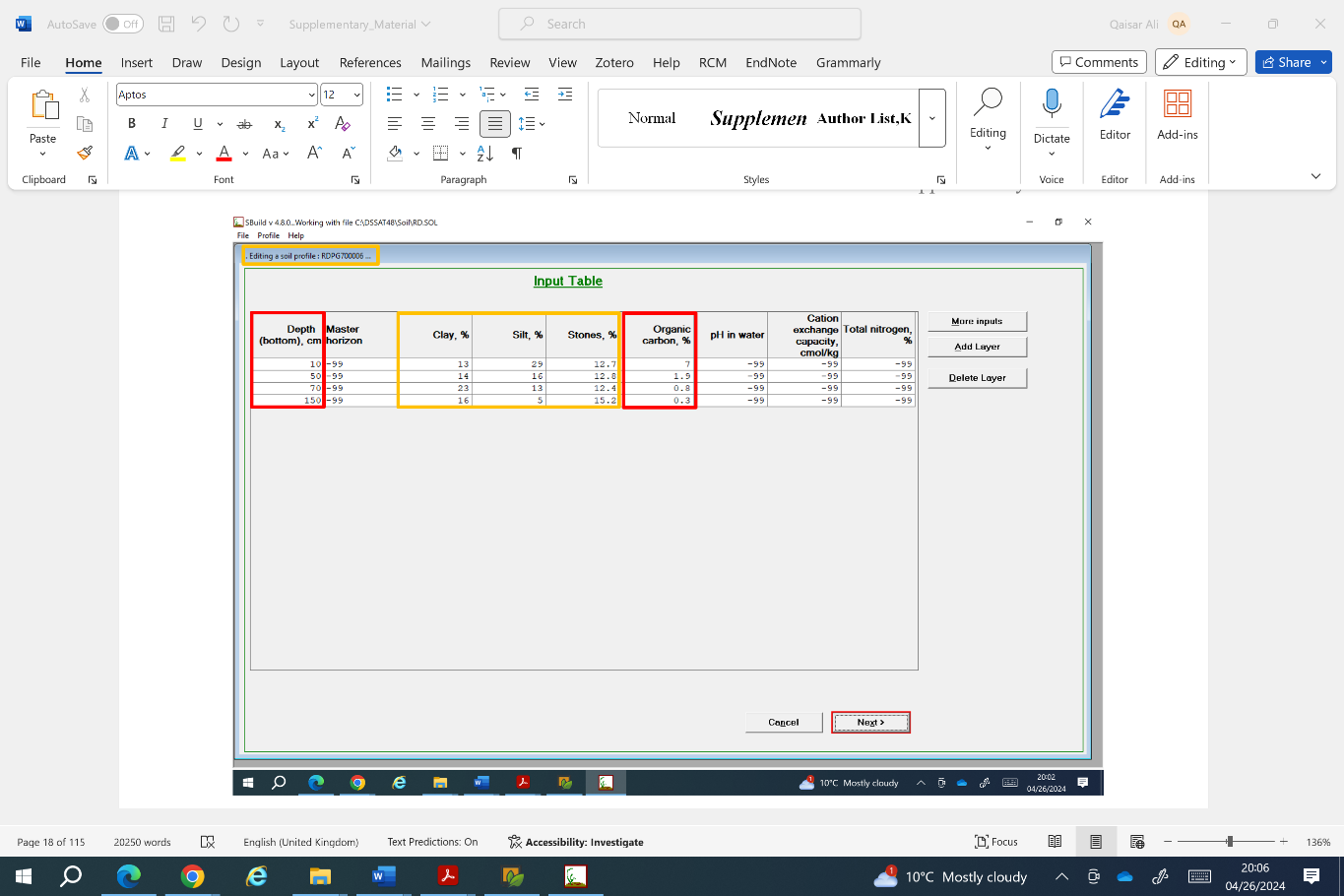


The above images and table show a crucial step for synthesis of the modelled soil PDPG700006 (first digit 7 represents OC level of 7%, and last digit 6 represents soilscape ID6 means PDPG6). The input data table was furnished with minimum data information on depth (cm), composition of clay (%), silt (%), stones (%) & organic carbon (%). There was no change in the textural composition to that of real soil PDGP6. Only OC levels for each depth were calculated as described in the upper paragraph. All remaining values of -99 represent missing values. The S-module of the DSSAT actioned to the next step which leads to the print screen image (calculate/ edit soil parameters) as under.


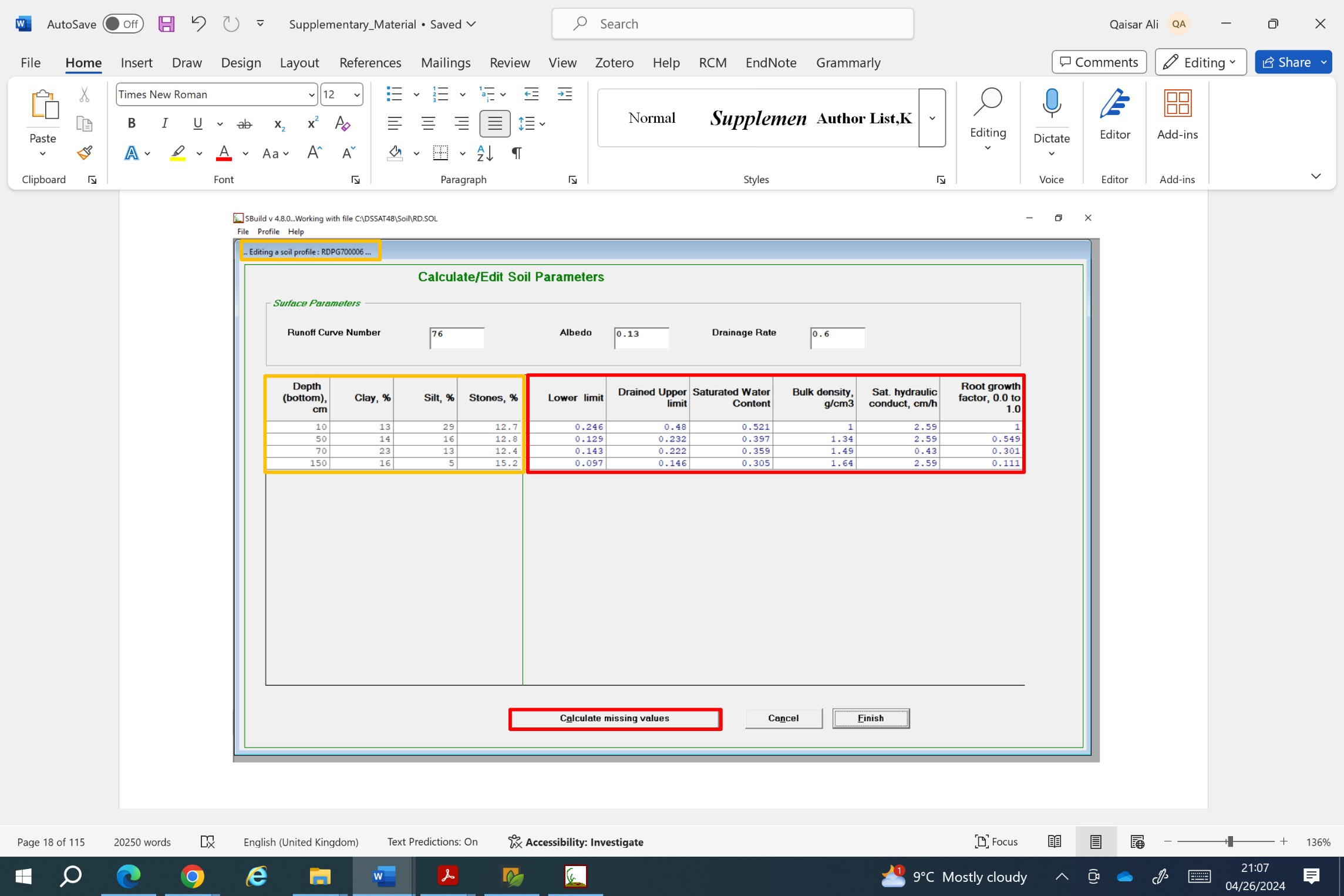


This image exhibits the interface for calculating soil parameters based on data incorporated at the earlier step in the S-module of the DSSAT. This screen shows two distinctive panels, *e.g.*, one with data in black and the other in blue-coloured digits. All blue-coloured digits were calculated by the auto-generation function performed by the S-module of the DSSAT by pressing the tab “Calculate missing values”, which gave the pedotransfer functional values for each layer. All these data values are summarized in Table S2. The process was completed to create an individual soil file for each soil in the RD.SOL soil profile section of the S-module of the DSSAT.

Likewise, all modelled soils were synthesized with varying OC levels for all layers. These were calculated based on their proportional OC levels between its upper and subsequent lower layers sequentially in the real soil. No change in soil depth/ zones and textural composition was made in the modelled soils relative to their real soil. All the soil water properties, *e.g.*, pedotransfer functional values, were calculated by the S-module of the DSSAT tool through its built-in auto-generating function of calculating missing values whilst introducing individual soil files. Please refer to the method of introducing soil files using the S-module in the DSSAT, the DSSAT Manual version 2 for details and the summary tables in supplementary material Tables S2, S3, and S4.

**Table S5 | *EXP.DETAILS: RORO7401WH N RESPONSE,ROTHAMSTED 8FE(N) (DSSAT3).**

***GENERAL**

| @PEOPLE  GODWIN,D |
| --- |
| @PEOPLE  GODWIN,D |
| @ADDRESS  ROTHAMSTED,ENGLAND,UK |
| @SITE  ROTHAMSTED,ENGLAND,UK 52.50;-0.50;100;ENG |
| *TREATMENTS -------------FACTOR LEVELS------------  @N R O C TNAME.................... CU FL SA IC MP MI MF MR MC MT ME MH SM  8 1 0 0 210 KG N/HA 1 1 0 1 1 0 8 0 0 0 0 0 1 |
| *CULTIVARS  @C CR INGENO CNAME  1 WH IB1015 MARIS FUNDEN |
| *FIELDS  @L ID_FIELD WSTA.... FLSA FLOB FLDT FLDD FLDS FLST SLTX SLDP ID_SOIL FLNAME  1 RORO0001 ROR1 -99 0 DR000 0 0 00000 -99 155 IBWH980020 -99 |
| @L ...........XCRD ...........YCRD .....ELEV .............AREA .SLEN .FLWR .SLAS FLHST FHDUR  1 0 0 0 0 0 0 0 -99 -99 |
| *INITIAL CONDITIONS  @C PCR ICDAT ICRT ICND ICRN ICRE ICWD ICRES ICREN ICREP ICRIP ICRID ICNAME  1 WH 74305 2000 0 1 1 -99 3000 .57 0 100 10 -99 |
| @C ICBL SH2O SNH4 SNO3  1 10 .33 3 6  1 25 .33 3 5  1 45 .33 3 4  1 65 .33 2 3  1 95 .33 2 2  1 125 .33 1 .5  1 155 .33 .5 .5 |
| *PLANTING DETAILS  @P PDATE EDATE PPOP PPOE PLME PLDS PLRS PLRD PLDP PLWT PAGE PENV PLPH SPRL PLNAME  1 74310 -99 277 277 S R 17 0 4 -99 -99 -99 -99 0 -99 |
| *IRRIGATION AND WATER MANAGEMENT  @I EFIR IDEP ITHR IEPT IOFF IAME IAMT IRNAME  1 1 30 50 100 GS000 IR001 10 -99  @I IDATE IROP IRVAL  1 74305 -99 -99 |
| *FERTILIZERS (INORGANIC)  @F FDATE FMCD FACD FDEP FAMN FAMP FAMK FAMC FAMO FOCD FERNAME  1 75108 FE001 AP001 10 0 0 0 0 0 -99 -99  2 75108 FE001 AP001 10 30 0 0 0 0 -99 -99  3 75108 FE001 AP001 10 60 0 0 0 0 -99 -99  4 75108 FE001 AP001 10 90 0 0 0 0 -99 -99  5 75108 FE001 AP001 10 120 0 0 0 0 -99 -99  6 75108 FE001 AP001 10 150 0 0 0 0 -99 -99  7 75108 FE001 AP001 10 180 0 0 0 0 -99 -99  8 75108 FE001 AP001 10 210 0 0 0 0 -99 -99 |
| *RESIDUES AND ORGANIC FERTILIZER  @R RDATE RCOD RAMT RESN RESP RESK RINP RDEP RMET RENAME  1 74305 -99 -99 -99 -99 -99 -99 -99 -99 -99 |
| *CHEMICAL APPLICATIONS  @C CDATE CHCOD CHAMT CHME CHDEP CHT..CHNAME  1 74305 -99 -99 -99 -99 -99 -99 |
| **TILLAGE AND ROTATIONS  @T TDATE TIMPL TDEP TNAME  1 74305 -99 -99 -99 |
| *HARVEST DETAILS  @H HDATE HSTG HCOM HSIZE HPC HBPC HNAME  1 74305 GS000 -99 -99 -99 -99 Wheat |
| *SIMULATION CONTROLS  @N GENERAL NYERS NREPS START SDATE RSEED SNAME.................... SMODEL  1 GE 1 1 S 74305 2150 N RESPONSE,ROTHAMSTED |
| @N OPTIONS WATER NITRO SYMBI PHOSP POTAS DISES CHEM TILL CO2  1 OP Y Y N N N N N N M |
| @N METHODS WTHER INCON LIGHT EVAPO INFIL PHOTO HYDRO NSWIT MESOM MESEV MESOL  1 ME M M E R S C R 1 G S 2 |
| @N MANAGEMENT PLANT IRRIG FERTI RESID HARVS  1 MA R R R N M |
| @N OUTPUTS FNAME OVVEW SUMRY FROPT GROUT CAOUT WAOUT NIOUT MIOUT DIOUT VBOSE CHOUT OPOUT FMOPT  1 OU N Y Y 1 Y N Y Y N N Y N N A |
| @ AUTOMATIC MANAGEMENT |
| @N PLANTING PFRST PLAST PH2OL PH2OU PH2OD PSTMX PSTMN  1 PL 74303 74317 40 100 30 40 10 |
| @N IRRIGATION IMDEP ITHRL ITHRU IROFF IMETH IRAMT IREFF  1 IR 30 50 100 GS000 IR001 10 1 |
| @N NITROGEN NMDEP NMTHR NAMNT NCODE NAOFF  1 NI 30 50 25 FE001 GS000 |
| @N RESIDUES RIPCN RTIME RIDEP  1 RE 100 1 20 |
| @N HARVEST HFRST HLAST HPCNP HPCNR  1 HA 0 75310 100 0 |
| **Comparative Tillage practices implementation for DSSAT simulations (Nutrient Management Guide (RB209) was updated in March 2022 by AHDB (Page no. 8) |

| No tillage - (NT) | Seed drill  Harvester | Sowing  Harvesting |
| --- | --- | --- |
| Conventional tillage - (CT) | Chisel plow, straight point (15)  Cultivator, row (7)  Plank (4)  Drill, double disc (5)  Rod weeder (3)  Fertilizer applicatior (5)  Fertilizer applicatior (5)  Fertilizer applicatior (5)  Fertilizer applicatior (5)  Harrow, Tine (3)  Harvestor (0) | Sowing  Herbicide & insecticide @ Growth stage (GS13 to GS21)  Fertilizer application @ Growth stage (GS21 to GS30)  PGR application @ Growth stage (GS30 to GS31)  Fertilizer application @ Growth stage (GS31)  Fungicide/ Hoeing @ Growth stage (GS32)  Fertilizer application @ Growth stage (GS37)  PGR application @ Growth stage (GS37)  Fungicide/ Hoeing @ Growth stage (GS39)  Fungicide/ Hoeing @ Growth stage (GS59)  Harvesting @ Growth stage (GS92) |


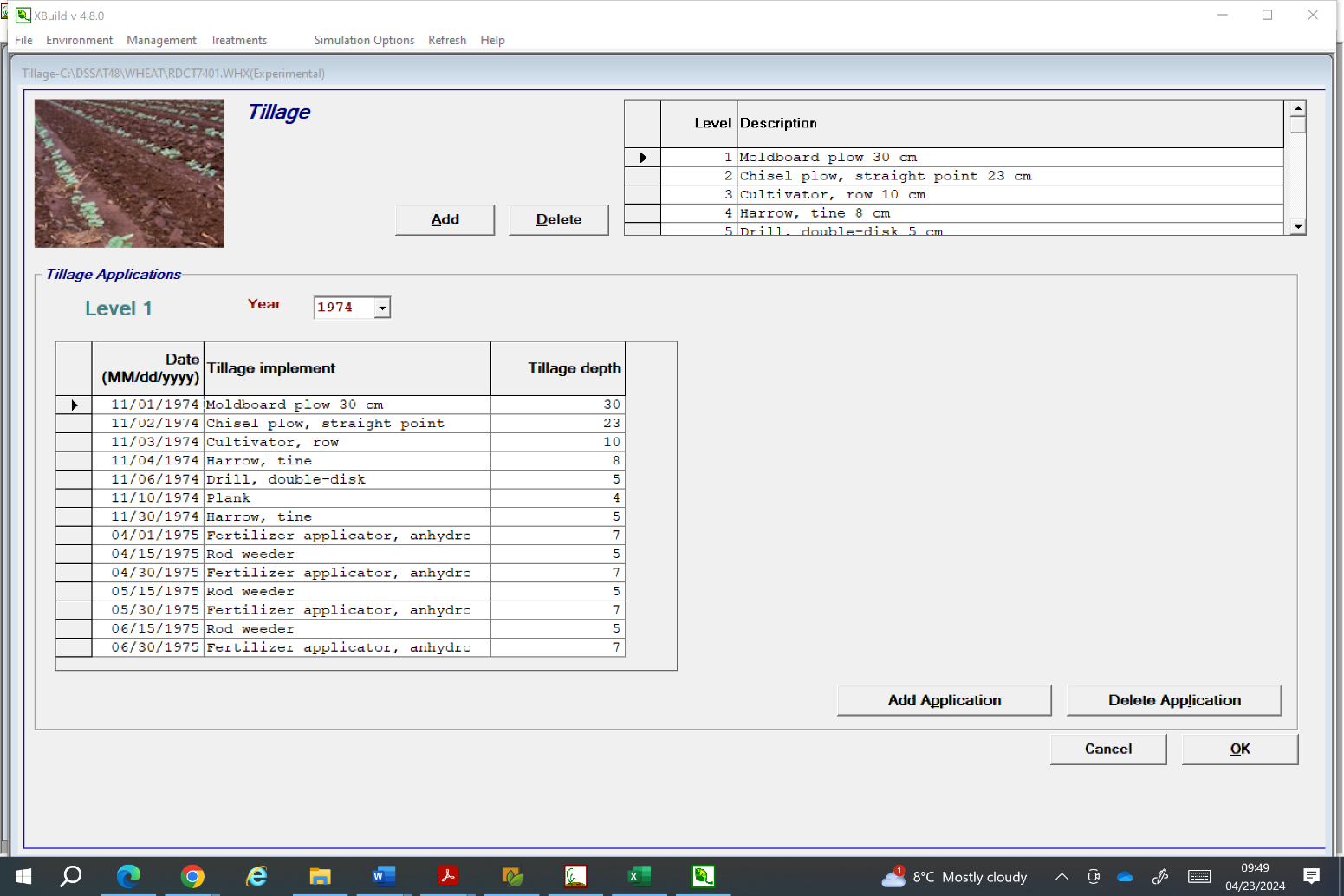


The above print screen exhibits the list of tillage applications introduced in the management tab of the X-module of the DSSAT for executing the simulations for conventional tillage (CT) experiments.

Contrarily, only no tillage except for sowing and harvesting were introduced in the management tab of the X-module of the DSSAT for executing the simulation for no-till (NT) experiments.

**Table S6 | Summary of modules for variables interactions applied in the DSSAT.**

| **Primary module** | **Sub module** | **Behavior** |
| --- | --- | --- |
| Main program (CSM) | - | This controls time steps and loops, which modules call built-on user input switches, and controls print timing for all modules. |
| Weather simulator | - | This reads out or generates daily weather parameters used by the model. Adjusts daily values if required and computes hourly values where applied. |
| Soil | Soil dynamic module | This computes soil structure characteristics by layer. Reads soil parameters and initial conditions and modifies to tillage and addition of organic matter. |
|  | Soil water module | This computes soil water processes, including snow accumulation and melt, runoff, infiltration, saturated flow and water table depth. Volumetric soil water content is updated daily for all soil layers. The tipping bucket approach is used. |
|  | Soil organic matter module | This computes organic matter transformations, including mineralization and immobilization of inorganic nutrients. Two organic matter modules are available in CSM: Century (Parton) and Ceres (Godwin). |
| Individual plant growth modules | CERES-Wheat | Modules that simulate growth and yield for individual species. Each module simulates phenology, daily growth and partitioning, plant nitrogen and carbon demands, senescence of plant material, *etc*. |

**Annexure to the table S6 is highlighting DSSAT modules, DSSAT output file, variable outputs serving input data for parametrization of the BBN.**


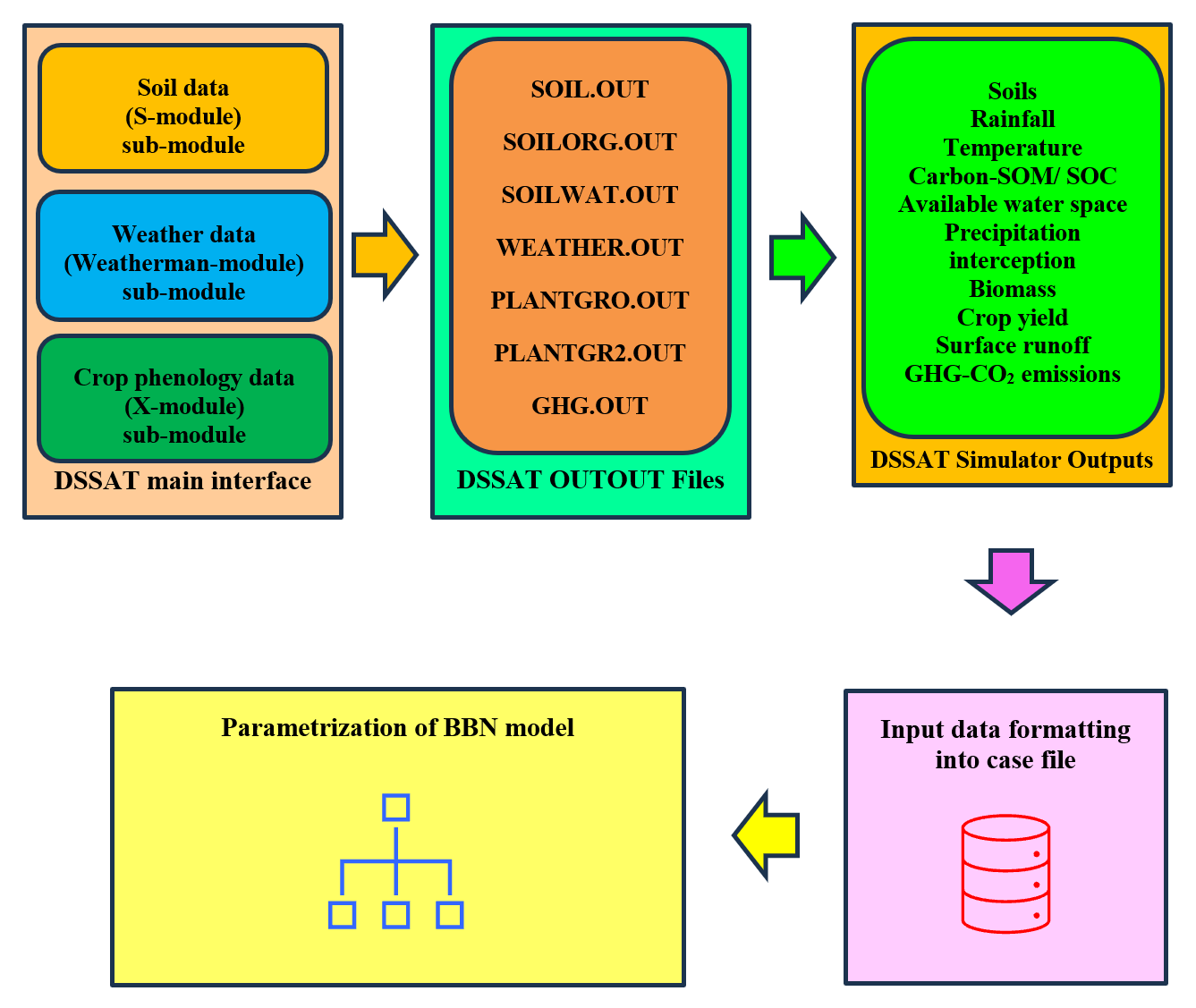


**Table S7 | List of variables with their relationships and evidence from the literature for BBN model structure.**

| **Sr. #** | **Variables relationship** | **References** |
| --- | --- | --- |
| 1 | Tillage  to  Carbon-Soil organic matter | i- Nicoloso RS, Amado TJ, Rice CW. Assessing strategies to enhance soil carbon sequestration with the DSSAT‐CENTURY model. European Journal of Soil Science. 2020 Nov;71(6):1034-49. <https://doi.org/10.1111/ejss.12938>. ii- Valboa G, Lagomarsino A, Brandi G, Agnelli AE, Simoncini S, Papini R, Vignozzi N, Pellegrini S. Long-term variations in soil organic matter under different tillage intensities. Soil and Tillage Research. 2015 Dec 1;154:126-35. <https://doi.org/10.1016/j.still.2015.06.017>. iii- Pezzuolo A, Dumont B, Sartori L, Marinello F, Migliorati MD, Basso B. Evaluating the impact of soil conservation measures on soil organic carbon at the farm scale. Computers and Electronics in Agriculture. 2017 Apr 1;135:175-82. <https://doi.org/10.1016/j.compag.2017.02.004>. iv- Okorie BO, Niraj Y. Effects Of Different Tillage Practices On Soil Fertility Properties: A Review. International Journal of Agriculture and Environmental Research. 2022 Feb 25;8(1):176-93. [10.22004/ag.econ.333826](http://dx.doi.org/10.22004/ag.econ.333826). v- Haddaway NR, Hedlund K, Jackson LE, Kätterer T, Lugato E, Thomsen IK, Jørgensen HB, Isberg PE. How does tillage intensity affect soil organic carbon? A systematic review. Environmental Evidence. 2017 Dec;6(1):1-48. DOI 10.1186/s13750-017-0108-9. vi- Iocola I, Bassu S, Farina R, Antichi D, Basso B, Bindi M, Dalla Marta A, Danuso F, Doro L, Ferrise R, Giglio L. Can conservation tillage mitigate climate change impacts in Mediterranean cereal systems? A soil organic carbon assessment using long-term experiments. European Journal of Agronomy. 2017 Oct 1;90:96-107. <https://doi.org/10.1016/j.eja.2017.07.011> |
| 2 | Tillage  to  Available water space | i- Wang S, Wang H, Hafeez MB, Zhang Q, Yu Q, Wang R, Wang X, Li J. No-tillage and subsoiling increased maise yields and soil water storage under varied rainfall distribution: A 9-year site-specific study in a semi-arid environment. Field Crops Research. 2020 Sep 15;255:107867. <https://doi.org/10.1016/j.fcr.2020.107867>. ii- Eeswaran R, Nejadhashemi AP, Kpodo J, Curtis ZK, Adhikari U, Liao H, Li SG, Hernandez-Suarez JS, Alves FC, Raschke A, Jha PK. Quantification of resilience metrics as affected by conservation agriculture at a watershed scale. Agriculture, Ecosystems & Environment. 2021 Oct 15;320:107612. <https://doi.org/10.1016/j.agee.2021.107612>. iii- Strudley MW, Green TR, Ascough II JC. Tillage effects on soil hydraulic properties in space and time: State of the science. Soil and Tillage Research. 2008 Apr 1;99(1):4-8. <https://doi.org/10.1016/j.still.2008.01.007>. iv- Salem HM, Valero C, Muñoz MÁ, Rodríguez MG, Silva LL. Short-term effects of four tillage practices on soil physical properties, soil water potential, and maize yield. Geoderma. 2015 Jan 1;237:60-70. <https://doi.org/10.1016/j.geoderma.2014.08.014>. |
| 3 | Soilscape (structure, texture, BD, *etc*.)  to  Available water space | i-Batjes NH. Developing a world data set of soil water retention properties using pedotransfer rules. Geoderma. 1996 May 1;71(1-2):31-52. <https://doi.org/10.1016/0016-7061(95)00089-5>. ii-Acutis M, Donatelli M. SOILPAR 2.00: software to estimate soil hydrological parameters and functions. European Journal of Agronomy. 2003 Jan 1;18(3-4):373-7. <https://doi.org/10.1016/S1161-0301(02)00128-4>. iii-Arya LM, Paris JF. A physicoempirical model to predict the soil moisture characteristic from particle‐size distribution and bulk density data. Soil Science Society of America Journal. 1981 Nov;45(6):1023-30. <https://doi.org/10.2136/sssaj1981.03615995004500060004x>. iv-Briggs LJ, McLane JW. The moisture equivalents of soils. US Government Printing Office; 1907. iv-Brasseur B, Ertlen D, Robin V. Soil Archives: Where Soilscape History Meets Present‐day Ecosystems. Historical Ecology: Learning from the Past to Understand the Present and Forecast the Future of Ecosystems. 2022 Sep 10:85-97. |
| 4 | Carbon-soil organic matter  to  Available water space | i-McDermid SS, Weng E, Puma M, Cook B, Hengl T, Sanderman J, De Lannoy GJ, Aleinov I. Soil carbon losses reduce soil moisture in global climate model simulations. Earth Interactions. 2022 Jan;26(1):195-208. <https://doi.org/10.1175/EI-D-22-0003.1>. ii-Intergovernmental Technical Panel on Soils, 2015: Status of the world's soil resources: Main report. FAO, 650 pp., <http://www.fao.org/3/a-i5199e.pdf>. iii-Manns, H. R., G. W. Parkin, and R. C. Martin, 2016: Evidence of a union between organic carbon and water content in the soil. Can. J. Soil Sci., **96**, 305–316, <https://doi.org/10.1139/cjss-2015-0084>. iv-Kane, D. A., M. A. Bradford, E. Fuller, E. E. Oldfield, and S. A. Wood, 2021: Soil organic matter protects US maize yields and lowers crop insurance payouts under drought. Environ. Res. Lett., **16**, 044018, <https://doi.org/10.1088/1748-9326/abe492>. |
| 5 | Carbon-soil organic matter  to  Biomass | i- Ryan J, Masri S, Singh M. Seasonal changes in soil organic matter and biomass and labile forms of carbon as influenced by crop rotations. Communications in soil science and plant analysis. 2009 Mar 1;40(1-6):188-99. <https://doi.org/10.1080/00103620802625617>. ii- Reicosky DC, Kemper WD, Langdale G, Douglas CL, Rasmussen PE. Soil organic matter changes resulting from tillage and biomass production. Journal of soil and water conservation. 1995 May 1;50(3):253-61. iii- Imran, Amanullah, Al Tawaha AR. Management of nano-black carbon, phosphorous and bio fertiliser improve soil organic carbon and ensilage biomass of soybean and maise. Communications in Soil Science and Plant Analysis. 2021 Dec 16;52(22):2837-51. <https://doi.org/10.1080/00103624.2021.1966439>. iv- Imran, Amanullah, Altawaha AR. Carbon assimilation and dry matter partitioning in soybean ease with the integration of nano-black carbon, along with beneficial microbes and phosphorus fertilisation. Journal of Plant Nutrition. 2022 Jul 21;45(12):1799-812. <https://doi.org/10.1080/01904167.2022.2035753>. |
| 6 | Carbon-soil organic matter  to  Total GHG-CO_2_ emission | i- Batlle-Bayer L, Batjes NH, Bindraban PS. Changes in organic carbon stocks upon land use conversion in the Brazilian Cerrado: a review. Agriculture, ecosystems & environment. 2010 Apr 15;137(1-2):47-58. <https://doi.org/10.1016/j.agee.2010.02.003>. ii-Bhattarai D, Abagandura GO, Nleya T, Kumar S. Responses of soil surface greenhouse gas emissions to nitrogen and sulfur fertilizer rates to Brassica carinata grown as a bio‐jet fuel. GCB Bioenergy. 2021 Apr;13(4):627-39. <https://doi.org/10.1111/gcbb.12784>. iii- Jebari A, Álvaro-Fuentes J, Pardo G, del Prado A. POTENTIAL IMPACTS OF CLIMATE CHANGE ON SOIL ORGANIC CARBON AND NET GHG EMISSIONS OF GRASSLANDS ASSOCIATED TO DAIRY PRODUCTION IN NORTHERN SPAIN. TESI DOCTORAL Integrating upscaling simulation methods for predicting soil organic Carbon changes in Spain. 2021:109. <https://idf-fundazioa.eus/wp-content/uploads/2022/06/Thesis_AsmaJebari_VF1-1.pdf#page=109>. iv- Suddick EC, Scow KM, Six JW. Do Agricultural Soils of California have the Potential to Sequester Carbon and Mitigate Greenhouse Gases? InAGU Fall Meeting Abstracts 2008 Dec (Vol. 2008, pp. B31G-0380). https://ui.adsabs.harvard.edu/abs/2008AGUFM.B31G0380S/abstract. |
| 7 | Rainfall  to  Available water space | i- Lin H, Duan X, Dong Y, Zhong R, Rong L, Huang J. Responses of soil water-holding capacity to environmental changes in alpine ecosystems across the southern Tibetan Plateau in the past 35–40 years. Catena. 2023 Mar 1;222:106840. <https://doi.org/10.1016/j.catena.2022.106840>. ii- Yoo C, Valdés JB, North GR. Evaluation of the impact of rainfall on soil moisture variability. Advances in Water Resources. 1998 Apr 15;21(5):375-84. <https://doi.org/10.1016/S0309-1708(97)00002-X>. iii- Yu X, Huang Y, Li E, Li X, Guo W. Effects of rainfall and vegetation to soil water input and output processes in the Mu Us Sandy Land, northwest China. Catena. 2018 Feb 1;161:96-103. <https://doi.org/10.1016/j.catena.2017.10.023>. iii- Cea L, Fraga I. Incorporating antecedent moisture conditions and intraevent variability of rainfall on flood frequency analysis in poorly gauged basins. Water Resources Research. 2018 Nov;54(11):8774-91. <https://doi.org/10.1029/2018WR023194>. iv- Haga H, Matsumoto Y, Matsutani J, Fujita M, Nishida K, Sakamoto Y. Flow paths, rainfall properties, and antecedent soil moisture controlling lags to peak discharge in a granitic unchanneled catchment. Water Resources Research. 2005 Dec;41(12). <https://doi.org/10.1029/2005WR004236>. |
| 8 | Rainfall  to  Precipitation interception | i- Jackson IJ. Relationships between rainfall parameters and interception by tropical forest. Journal of Hydrology. 1975 Feb 1;24(3-4):215-38. <https://doi.org/10.1016/0022-1694(75)90082-7>. ii-Clark OR. Interception of rainfall by prairie grasses, weeds, and certain crop plants. Ecological monographs. 1940 Apr 1;10(2):243-77. <https://doi.org/10.2307/1948607>. iii- Leuning R, Condon AG, Dunin FX, Zegelin S, Denmead OT. Rainfall interception and evaporation from the soil below a wheat canopy. Agricultural and Forest Meteorology. 1994 Jan 1;67(3-4):221-38. <https://doi.org/10.1016/0168-1923(94)90004-3>. iv- Xiao Q, McPherson EG, Ustin SL, Grismer ME, Simpson JR. Winter rainfall interception by two mature open‐grown trees in Davis, California. Hydrological processes. 2000 Mar;14(4):763-84. [https://doi.org/10.1002/(SICI)1099-1085(200003)14:4<763::AID-HYP971>3.0.CO;2-7](https://doi.org/10.1002/(SICI)1099-1085(200003)14:4%3C763::AID-HYP971%3E3.0.CO;2-7). |
| 9 | Rainfall  to  runoff | i- Sivakumar B, Berndtsson R, Olsson J, Jinno K. Evidence of chaos in the rainfall-runoff process. Hydrological Sciences Journal. 2001 Feb 1;46(1):131-45. Sivakumar B, Berndtsson R, Olsson J, Jinno K. Evidence of chaos in the rainfall-runoff process. Hydrological Sciences Journal. 2001 Feb 1;46(1):131-45. ii- Wilkinson ME, Quinn PF, Welton P. Runoff management during the September 2008 floods in the Belford catchment, Northumberland. Journal of Flood Risk Management. 2010 Dec;3(4):285-95. <https://doi.org/10.1111/j.1753-318X.2010.01078.x>. iii- Miller CR, Viessman Jr W. Runoff volumes from small urban watersheds. Water Resources Research. 1972 Apr;8(2):429-34. <https://doi.org/10.1029/WR008i002p00429>. iv- Agassi M, Shainberg I, Morin J. Infiltration and runoff in wheat fields in the semi-arid region of Israel. Geoderma. 1985 Dec 1;36(3-4):263-76. <https://doi.org/10.1016/0016-7061(85)90007-2>. iv- Tao W, Wang Q, Guo L, Lin H, Chen X, Sun Y, Ning S. An enhanced rainfall–runoff model with coupled canopy interception. Hydrological Processes. 2020 Apr 15;34(8):1837-53. <https://doi.org/10.1002/hyp.13696>. |
| 10 | Rainfall  to  biomass | i- Kong DL, Lü XT, Jiang LL, Wu HF, Miao Y, Kardol P. Extreme rainfall events can alter inter-annual biomass responses to water and N enrichment. Biogeosciences. 2013 Dec 11;10(12):8129-38. <https://doi.org/10.5194/bg-10-8129-2013>. ii- Walter J, Grant K, Beierkuhnlein C, Kreyling J, Weber M, Jentsch A. Increased rainfall variability reduces biomass and forage quality of temperate grassland largely independent of mowing frequency. Agriculture, Ecosystems & Environment. 2012 Feb 15;148:1-0. <https://doi.org/10.1016/j.agee.2011.11.015>. iii- Hovenden MJ, Newton PC, Wills KE. Seasonal, not annual, rainfall determines grassland biomass response to carbon dioxide. Nature. 2014 Jul 31;511(7511):583-6. DOIhttps://doi.org/10.1038/nature13281. iv- Bai L, Shan L, Li Y, Zhang Z, Chong P. Effects of changing rainfall patterns on the root and biomass of Reaumuria soongorica seedlings. Acta Botanica Boreali-Occidentalia Sinica. 2017;37(1):163-70. v- Gibson-Forty EV, Barnett KL, Tissue DT, Power SA. Reducing rainfall amount has a greater negative effect on the productivity of grassland plant species than reducing rainfall frequency. Functional Plant Biology. 2016 Feb 16;43(4):380-91. |
| 11 | Available water space  to  runoff | i- Crow WT, Dong J, Reichle RH. Leveraging Pre‐Storm Soil Moisture Estimates for Enhanced Land Surface Model Calibration in Ungauged Hydrologic Basins. Water Resources Research. 2022 Aug;58(8):e2021WR031565. <https://doi.org/10.1029/2021WR031565> ii- Clark DB, Gedney N. Representing the effects of subgrid variability of soil moisture on runoff generation in a land surface model. Journal of Geophysical Research: Atmospheres. 2008 May 27;113(D10). <https://doi.org/10.1029/2007JD008940>. iii- Crow WT, Chen F, Reichle RH, Xia Y, Liu Q. Exploiting soil moisture, precipitation, and streamflow observations to evaluate soil moisture/runoff coupling in land surface models. Geophysical research letters. 2018 May 28;45(10):4869-78. <https://doi.org/10.1029/2018GL077193>. iv- Crow WT, Dong J, Reichle RH. Leveraging Pre‐Storm Soil Moisture Estimates for Enhanced Land Surface Model Calibration in Ungauged Hydrologic Basins. Water Resources Research. 2022 Aug;58(8):e2021WR031565. <https://doi.org/10.1029/2002JD003090>. |
| 12 | Temperature  to  Biomass | i- Xiao L, Asseng S, Wang X, Xia J, Zhang P, Liu L, Tang L, Cao W, Zhu Y, Liu B. Simulating the effects of low-temperature stress on wheat biomass growth and yield. Agricultural and Forest Meteorology. 2022 Nov 15;326:109191. <https://doi.org/10.1016/j.agrformet.2022.109191>. ii- FERRIS R, Ellis RH, Wheeler TR, Hadley P. Effect of high-temperature stress at anthesis on grain yield and biomass of field-grown crops of wheat. Annals of Botany. 1998 Nov 1;82(5):631-9. <https://doi.org/10.1006/anbo.1998.0740>. iii-Wang L, Hu W, Zahoor R, Yang X, Wang Y, Zhou Z, Meng Y. Cool temperature caused by late planting affects seed vigor via altering kernel biomass and antioxidant metabolism in cotton (Gossypium hirsutum L.). Field Crops Research. 2019 Apr 15;236:145-54. <https://doi.org/10.1016/j.fcr.2019.04.002>. iv- Kiniry JR, Jones CA, O'toole JC, Blanchet R, Cabelguenne M, Spanel DA. Radiation-use efficiency in biomass accumulation prior to grain-filling for five grain-crop species. Field Crops Research. 1989 Feb 1;20(1):51-64. <https://doi.org/10.1016/0378-4290(89)90023-3>. |
| 13 | Temperature  to  Total GHG-CO_2_ emission | i- Wheeler TR, Batts GR, Ellis RH, Hadley P, Morison JI. Growth and yield of winter wheat (Triticum aestivum) crops in response to CO2 and temperature. The Journal of Agricultural Science. 1996 Aug;127(1):37-48. DOI: <https://doi.org/10.1017/S0021859600077352>. ii- Greenhouse Gas Working Group. Agriculture's role in greenhouse gas emissions and capture. Madison, WI: Greenhouse Gas Working Group Rep. ASA, CSSA, and SSSA; 2010. <http://hdl.handle.net/10919/68928>. iii- Kalvakaalva R, Prior SA, Smith M, Runion GB, Ayipio E, Blanchard C, Wall N, Wells D, Hanson TR, Higgins BT. Direct Greenhouse Gas Emissions From a Pilot-Scale Aquaponics System. Journal of the ASABE. 2022;65(6):1211-23. doi: 10.13031/ja.15215. iv- Cai C, Yin X, He S, Jiang W, Si C, Struik PC, Luo W, Li G, Xie Y, Xiong Y, Pan G. Responses of wheat and rice to factorial combinations of ambient and elevated CO2 and temperature in FACE experiments. Global change biology. 2016 Feb;22(2):856-74. <https://doi.org/10.1111/gcb.13065>. |
| 14 | Biomass  to  Total GHG-CO_2_ emission | i- Grönkvist S, Möllersten K, Pingoud K. Equal opportunity for biomass in greenhouse gas accounting of CO 2 capture and storage: A step towards more cost-effective climate change mitigation regimes. Mitigation and Adaptation Strategies for Global Change. 2006 Sep;11:1083-96. DOI: 10.1007/s11027-006-9034-9. ii- Grönkvist S, Möllersten K, Pingoud K. Equal opportunity for biomass in greenhouse gas accounting of CO 2 capture and storage: A step towards more cost-effective climate change mitigation regimes. Mitigation and Adaptation Strategies for Global Change. 2006 Sep;11:1083-96. Grönkvist S, Möllersten K, Pingoud K. Equal opportunity for biomass in greenhouse gas accounting of CO 2 capture and storage: A step towards more cost-effective climate change mitigation regimes. Mitigation and Adaptation Strategies for Global Change. 2006 Sep;11:1083-96. iii- Borgwardt RH. Transportation fuel from cellulosic biomass: a comparative assessment of ethanol and methanol options. Proceedings of the Institution of Mechanical Engineers, Part A: Journal of Power and Energy. 1999 Aug 1;213(5):399-407. <https://doi.org/10.1243/0957650991537770>. iv- Gan J, Smith CT. Biomass utilization allocation in biofuel production: model and application. International journal of forest engineering. 2012 Dec 1;23(1):38-47. <https://doi.org/10.1080/14942119.2012.10739959>. |
| 15 | Biomass  to  precipitation interception | i- Gilliam FS, Seastedt TR, Knapp AK. Canopy rainfall interception and throughfall in burned and unburned tallgrass prairie. The Southwestern Naturalist. 1987 Jun 15:267-71. <https://doi.org/10.2307/3671570>. ii- Wilson SD, Schlaepfer DR, Bradford JB, Lauenroth WK, Duniway MC, Hall SA, Jamiyansharav K, Jia G, Lkhagva A, Munson SM, Pyke DA. Functional group, biomass, and climate change effects on ecological drought in semi-arid grasslands. Journal of Geophysical Research: Biogeosciences. 2018 Mar;123(3):1072-85. <https://doi.org/10.1002/2017JG004173>. iii- Baroni G, Oswald SE. A scaling approach for the assessment of biomass changes and rainfall interception using cosmic-ray neutron sensing. Journal of Hydrology. 2015 Jun 1;525:264-76. <https://doi.org/10.1016/j.jhydrol.2015.03.053>. iv- Van Dijk AI, Bruijnzeel LA. Modelling rainfall interception by vegetation of variable density using an adapted analytical model. Part 2. Model validation for a tropical upland mixed cropping system. Journal of Hydrology. 2001 Jul 2;247(3-4):239-62. <https://doi.org/10.1016/S0022-1694(01)00393-6>. v- Yan T, Wang Z, Liao C, Xu W, Wan L. Effects of the morphological characteristics of plants on rainfall interception and kinetic energy. Journal of Hydrology. 2021 Jan 1;592:125807. <https://doi.org/10.1016/j.jhydrol.2020.125807>. |
| 16 | Biomass  to  Crop yield | i- Wu A, Hammer GL, Doherty A, von Caemmerer S, Farquhar GD. Quantifying impacts of enhancing photosynthesis on crop yield. Nature plants. 2019 Apr;5(4):380-8. V <https://doi.org/10.1038/s41477-019-0398-8>. ii- Cierjacks A, Pommeranz M, Schulz K, Almeida-Cortez J. Is crop yield related to weed species diversity and biomass in coconut and banana fields of northeastern Brazil? Agriculture, Ecosystems & Environment. 2016 Mar 15;220:175-83. <https://doi.org/10.1016/j.agee.2016.01.006>. iii- Boe A, Beck DL. Yield components of biomass in switchgrass. Crop Science. 2008 Jul;48(4):1306-11. <https://doi.org/10.2135/cropsci2007.08.0482>. iv- Mullet JE. High-biomass C4 grasses—Filling the yield gap. Plant Science. 2017 Aug 1;261:10-7. <https://doi.org/10.1016/j.plantsci.2017.05.003>. |
| 17 | Precipitation interception  to  runoff | i- Effects of driving factors at multi-spatial scales on seasonal runoff and sediment changes. Catena. 2023 Mar 1;222:106867. <https://doi.org/10.1016/j.catena.2022.106867>. ii- Kozak JA, Ahuja LR, Green TR, Ma L. Modelling crop canopy and residue rainfall interception effects on soil hydrological components for semi‐arid agriculture. Hydrological Processes: An International Journal. 2007 Jan 15;21(2):229-41. <https://doi.org/10.1002/hyp.6235>. iii- Agassi M, Shainberg I, Morin J. Infiltration and runoff in wheat fields in the semi-arid region of Israel. Geoderma. 1985 Dec 1;36(3-4):263-76. <https://doi.org/10.1016/0016-7061(85)90007-2>. iv- Tao W, Wang Q, Guo L, Lin H, Chen X, Sun Y, Ning S. An enhanced rainfall–runoff model with coupled canopy interception. Hydrological Processes. 2020 Apr 15;34(8):1837-53. <https://doi.org/10.1002/hyp.13696>. |

**Table S8 | The following are the parametric and *discretization details of the BBN.**

| **Sr. No.** | **Nodes (Variable) Name** | **Nodes State titles** | **Node *Discretization** | **Description** |
| --- | --- | --- | --- | --- |
| 1 | Tillage | No-till (NT) Conventional till (CT) | 0-1.99 1.99-2.99 | The main study variable widely used is tillage as NT or CT. |
| 2 | Soilscape | PDPG6 PDPG7 PDPG8 | 0-6.99 6.99-7.99 7.99-8.99 | Local soil representation from the Pang catchment area. |
| 3 | Rainfall (seasonal mean) (mm) | Very low Low Medium High Very high | 195-294 294-393 393-492 492-591 591-690 | Local climatic and weather data influence Wheat crop production & runoff with potential flooding risks. |
| 4 | Temperature (seasonal mean) (C°) | Very low Low Medium High Very high | 7.7-8.19 8.19-8.69 8.69-9.19 9.19-9.69 9.69-10.2 | Local climatic and weather data influence Wheat crop production & runoff with potential flooding risks. |
| 5 | Carbon-Soil organic matter (kg/ha) | Very low Low Medium High Very high | 5890-62052 62052-118214 118214-174376 174376-230538 230538-286700 | Local soilscapes representation with real soils & modelled soils with varying organic carbon (contents). |
| 6 | Available water space (cm^3^ cm^3^) | Very low Low Medium High Very high | 125-206 206-287 287-368 368-449 449-530 | Synthetic datasets obtained through simulation executed in the DSSAT represent AWS in representative soils. |
| 7 | Precipitation interception (mm) | Very low Low Medium High Very high | 195-294 294-393 393-492 492-591 591-690 | Synthetic datasets obtained through simulation executed in the DSSAT represent ppt interception by representative soils. |
| 8 | Biomass (kg/ha) | Very low Low Medium High Very high | 465-4984 4984-9503 9503-14022 14022-18541 18541-23065 | Synthetic datasets obtained via simulations executed in the DSSAT and represent total wt. gained by representative soils. |
| 9 | GHG-CO_2_ emission (eqv. kg/ha) | Very low Low Medium High Very high | 2250.54-8766 8766-15277 15277-21788 21788-28299 28299-34810 | Synthetic datasets obtained via simulations executed in the DSSAT represent total GHG-CO_2_ by representative soils. |
| 10 | Crop yield (kg/ha) | Very low Low Medium High Very high | 170-1851 1851-3532 3532-5213 5213-6894 6894-8597.45 | Synthetic datasets obtained via simulations executed in the DSSAT represent harvested wt. by representative soils. |
| 11 | Runoff (mm) | Very low Low Medium High Very high | 0-9 9-18 18-27 27-36 36-45 | Synthetic datasets are obtained via simulations executed in the DSSAT and represent surface runoff by representative soils. |

|  | **Rainfall** | **Temp** | **Biomass** | **Yield** | **Available Water** | **Surface Runoff** | **C-SOM** | **GHG-CO_2_ Emission** | **PPT interception** |
| --- | --- | --- | --- | --- | --- | --- | --- | --- | --- |
| **minimum** | **196.30** | **7.73** | **470.00** | **174.00** | **126.60** | **0.00** | **5894.00** | **2258.00** | **0.11** |
| **maximum** | **690.00** | **10.18** | **23064.00** | **8575.00** | **527.81** | **44.00** | **286634.00** | **34806.00** | **0.59** |
| **average** | **447.44** | **8.99** | **10233.52** | **3994.03** | **294.40** | **8.44** | **140009.47** | **8289.27** | **0.38** |
| **standard deviation** | ***97.86*** | ***0.58*** | ***4595.24*** | ***1879.89*** | ***125.23*** | ***6.92*** | ***71084.34*** | ***4854.09*** | ***0.09*** |
|  | **Rainfall** | **Temp** | **Biomass** | **Yield** | **Available Water** | **Surface Runoff** | **C-SOM** | **GHG-CO_2_ Emission** | **PPT interception** |
| ***Discretization (Bin size)** | ***99*** | ***0.5*** | ***4519*** | ***1681*** | ***81*** | ***9*** | ***56162*** | ***6515*** | ***99*** |

**Table S8a** ****Discretization (defining bin size/ nominal categories)***

****Discretization (defining bin size/ nominal categories) is primarily based on standard deviation in most of variables. For remaining variables, difference between maximum value and minimum values of the given variables was calculated and divided by five equal categories to define discretized bin sizes or nominal categories.***

| **Clique (Joined to)** | **Size** | **Member nodes (*mean home)** |
| --- | --- | --- |
| 0 [1] | 750 | *Soilscape, *Tillage, *C-SOM, Precipitation, *Available water space. |
| 1 [0 2 4] | 625 | Biomass, C-SOM, *Rainfall, Available water space. |
| 2 [1 3] | 625 | *Temperature, *Biomass, C-SOM, Rainfall. |
| 3 [2] | 625 | *GHG-CO_2_, Temperature, Biomass, C-SOM. |
| 4 [1 5 6] | 625 | *Precipitation interception, Biomass, Rainfall, Available water space. |
| 5 [4] | 625 | *Runoff, Precipitation interception, Rainfall, Available water space. |
| 6 [4] | 25 | *Crop yield, biomass |

**Table S9 | Junction tree showing the structural morphology of the BBN model.**

Sum of clique sizes = 3900 (with sepsets = 4530)

**Table S10a | Sensitivity of “GHG-CO_2_ Emissions– (kg/ha)” to a finding at another node.**

| **Node** | **Variance Reduction** | **Percent** | **Mutual Info** | **Percent** | **Variance of Beliefs** |
| --- | --- | --- | --- | --- | --- |
| Total GHG-CO2 emission | 129.7 | 100 | 1.97235 | 100 | 0.507211 |
| Biomass | 7.749 | 5.97 | 0.07297 | 3.7 | 0.0115946 |
| Carbon-Soil organic matter | 6.684 | 5.15 | 0.04411 | 2.24 | 0.0025744 |
| Crop yield | 3.76 | 2.9 | 0.04861 | 2.46 | 0.004495 |
| Temperature | 1.317 | 1.02 | 0.00999 | 0.507 | 0.000577 |
| Precipitation interception | 0.8228 | 0.634 | 0.01043 | 0.529 | 0.0018494 |
| Rainfall | 0.6154 | 0.474 | 0.00506 | 0.257 | 0.0002651 |
| Available water space | 0.1676 | 0.129 | 0.00258 | 0.131 | 0.0003944 |
| Runoff | 0.09602 | 0.074 | 0.0006 | 0.0302 | 0.0000582 |
| Tillage | 0.04547 | 0.0351 | 0.00038 | 0.019 | 0.000033 |
| Soilscape | 0.0003372 | 0.00026 | 0 | 0.00019 | 0.0000005 |

**Table S10b | Sensitivity of “Runoff – (ROFC) mm” to a finding at another node.**

| **Node** | **Variance Reduction** | **Percent** | **Mutual Info** | **Percent** | **Variance of Beliefs** |
| --- | --- | --- | --- | --- | --- |
| Runoff | 129.7 | 100 | 1.97235 | 100 | 0.507211 |
| Available water space | 7.749 | 5.97 | 0.07297 | 3.7 | 0.0115946 |
| Precipitation interception | 6.684 | 5.15 | 0.04411 | 2.24 | 0.0025744 |
| Rainfall | 3.76 | 2.9 | 0.04861 | 2.46 | 0.004495 |
| Biomass | 1.317 | 1.02 | 0.00999 | 0.507 | 0.000577 |
| Soilscape | 0.8228 | 0.634 | 0.01043 | 0.529 | 0.0018494 |
| Crop yield | 0.6154 | 0.474 | 0.00506 | 0.257 | 0.0002651 |
| Carbon-Soil organic carbon | 0.1676 | 0.129 | 0.00258 | 0.131 | 0.0003944 |
| Total GHG-CO_2_ Emissions | 0.09602 | 0.074 | 0.0006 | 0.0302 | 0.0000582 |
| Temperature | 0.04547 | 0.0351 | 0.00038 | 0.019 | 0.000033 |
| Tillage | 0.0003372 | 0.00026 | 0 | 0.00019 | 0.0000005 |

**Table S10c | Sensitivity of “Crop yield – (Kg/ha)” to a finding at another node.**

| **Node** | **Variance Reduction** | **Percent** | **Mutual Info** | **Percent** | **Variance of Beliefs** |
| --- | --- | --- | --- | --- | --- |
| Crop yield | 4.50E+06 | 100 | 2.22687 | 100 | 0.607791 |
| Biomass | 3.00E+06 | 66.7 | 0.92292 | 41.4 | 0.1272802 |
| Precipitation interception | 1.16E+06 | 25.8 | 0.2734 | 12.3 | 0.0231499 |
| Carbon-Soil organic matter | 1.91E+05 | 4.24 | 0.03634 | 1.63 | 0.0025176 |
| Total GHG-CO_2_ Emissions | 4.36E+04 | 0.969 | 0.01589 | 0.714 | 0.000763 |
| Temperature | 3.49E+04 | 0.775 | 0.00918 | 0.412 | 0.0005237 |
| Rainfall | 1.81E+04 | 0.403 | 0.00469 | 0.211 | 0.0002222 |
| Runoff | 1.22E+04 | 0.272 | 0.00506 | 0.227 | 0.0003673 |
| Available water space | 1.02E+04 | 0.226 | 0.00192 | 0.0862 | 0.0001175 |
| Tillage | 1509 | 0.0335 | 0.0003 | 0.0135 | 0.0000196 |
| Soilscape | 0 | 0 | 0 | 0 | 0 |

**Table S10d | Sensitivity of “Available Water Space (AWS) – (cm^3^ cm^3^)” to a finding at another node.**

| **Node** | **Variance Reduction** | **Percent** | **Mutual Info** | **Percent** | **Variance of Beliefs** |
| --- | --- | --- | --- | --- | --- |
| Available water space | 1.576e+04 | 100 | 2.24986 | 100 | 0.6133276 |
| Soilscape | 4415 | 28 | 0.37863 | 16.8 | 0.0541467 |
| Carbon-Soil organic matter | 473.1 | 3 | 0.09115 | 4.05 | 0.0059691 |
| Runoff | 251.2 | 1.59 | 0.07297 | 3.24 | 0.0049217 |
| Biomass | 29.63 | 0.188 | 0.00300 | 0.133 | 0.0002186 |
| Total GHG-CO_2_ Emissions | 21.38 | 0.136 | 0.00207 | 0.0918 | 0.0001251 |
| Crop yield | 20.09 | 0.127 | 0.00192 | 0.0854 | 0.0001419 |
| Precipitation interception | 11.49 | 0.0729 | 0.00132 | 0.587 | 0.0000915 |
| Tillage | 8.029 | 0.0509 | 0.00083 | 0.0368 | 0.0000408 |
| Rainfall | 5.498 | 0.0349 | 0.00483 | 0.215 | 0.0001977 |
| Temperature | 0 | 0 | 0.00000 | 0 | 0.0000000 |

**Table S11 | Specimen case file data with *training cases of 75% dataset (1975-2011)* simulated by the DSSAT tool for analyzing CT vs NT for the response variable, considering soilscape PDPG 6,7,8 with varying OC levels of 1,3,5,7%. (Full case file with test cases of 75% datasets from 1975-2011 is available separately in the folder of supplementary documentation.)**

| **IDnum** | **PRED** | **TMEAN** | **TWAD** | **HWAD** | **AWS** | **ROFC** | **SOMC** | **GHG** | **PARID** | **OCLEVEL** | **SOILSCAPE** | **TILLAGE** |
| --- | --- | --- | --- | --- | --- | --- | --- | --- | --- | --- | --- | --- |
| 1975 | 502.20 | 9.24 | 4966 | 2065 | 239 | 11.00 | 96281 | 8188 | 0.31 | 2.6 | 6 | 2 |
| 1976 | 196.30 | 8.90 | 4092 | 1794 | 230 | 2.00 | 96294 | 6437 | 0.25 | 2.6 | 6 | 2 |
| 1977 | 549.20 | 8.03 | 6953 | 2395 | 243 | 7.00 | 96300 | 8089 | 0.22 | 2.6 | 6 | 2 |
| 1978 | 507.00 | 8.26 | 7315 | 3283 | 240 | 6.00 | 96323 | 8662 | 0.31 | 2.6 | 6 | 2 |
| 1979 | 584.70 | 7.73 | 8513 | 4301 | 244 | 4.00 | 96319 | 8847 | 0.31 | 2.6 | 6 | 2 |
| 1980 | 464.80 | 8.36 | 4207 | 1136 | 238 | 3.00 | 96317 | 8520 | 0.23 | 2.6 | 6 | 2 |
| 1981 | 425.00 | 8.65 | 11517 | 5311 | 242 | 0.00 | 96319 | 9337 | 0.42 | 2.6 | 6 | 2 |
| 1982 | 444.20 | 8.16 | 5843 | 1828 | 239 | 4.00 | 96304 | 8152 | 0.24 | 2.6 | 6 | 2 |
| 1983 | 487.30 | 8.74 | 4308 | 899 | 251 | 3.00 | 96312 | 11036 | 0.37 | 2.6 | 6 | 2 |
| 1984 | 419.20 | 8.49 | 5294 | 2653 | 238 | 5.00 | 96334 | 8496 | 0.27 | 2.6 | 6 | 2 |
| 1985 | 537.90 | 8.08 | 10261 | 4114 | 241 | 5.00 | 96299 | 8732 | 0.29 | 2.6 | 6 | 2 |
| 1986 | 451.40 | 7.94 | 5798 | 2889 | 241 | 5.00 | 96328 | 8777 | 0.29 | 2.6 | 6 | 2 |
| 1987 | 472.20 | 8.41 | 5311 | 2281 | 238 | 0.00 | 96307 | 8279 | 0.24 | 2.6 | 6 | 2 |
| 1988 | 467.10 | 8.92 | 7504 | 2690 | 232 | 1.00 | 96326 | 8217 | 0.32 | 2.6 | 6 | 2 |
| 1989 | 273.20 | 9.52 | 8779 | 4348 | 233 | 0.00 | 96315 | 7794 | 0.41 | 2.6 | 6 | 2 |
| 1990 | 448.50 | 9.60 | 1967 | 753 | 233 | 14.00 | 96273 | 6861 | 0.21 | 2.6 | 6 | 2 |
| 1991 | 422.90 | 8.48 | 8097 | 2698 | 237 | 3.00 | 96317 | 8575 | 0.32 | 2.6 | 6 | 2 |
| 1992 | 304.00 | 9.03 | 11778 | 5255 | 238 | 1.00 | 96320 | 8450 | 0.43 | 2.6 | 6 | 2 |
| 1993 | 446.50 | 9.08 | 5964 | 2124 | 238 | 4.00 | 96298 | 7978 | 0.29 | 2.6 | 6 | 2 |
| 1994 | 435.40 | 8.87 | 7831 | 3827 | 244 | 0.00 | 96313 | 9088 | 0.35 | 2.6 | 6 | 2 |
| 1995 | 454.20 | 9.76 | 3516 | 1621 | 232 | 4.00 | 96289 | 7213 | 0.28 | 2.6 | 6 | 2 |
| 1996 | 421.90 | 8.37 | 545 | 205 | 256 | 12.00 | 96332 | 11544 | 0.20 | 2.6 | 6 | 2 |
| 1997 | 331.70 | 8.68 | 4323 | 1410 | 237 | 2.00 | 96309 | 7594 | 0.24 | 2.6 | 6 | 2 |
| 1998 | 511.50 | 9.71 | 11046 | 3016 | 248 | 8.00 | 96277 | 10967 | 0.46 | 2.6 | 6 | 2 |
| 1999 | 462.20 | 9.48 | 7321 | 1894 | 246 | 11.00 | 96293 | 9928 | 0.41 | 2.6 | 6 | 2 |
| 2000 | 489.30 | 9.13 | 11372 | 5008 | 240 | 5.00 | 96315 | 9547 | 0.41 | 2.6 | 6 | 2 |
| 2001 | 616.60 | 8.87 | 6370 | 2183 | 245 | 6.00 | 96290 | 9037 | 0.29 | 2.6 | 6 | 2 |
| 2002 | 413.10 | 9.01 | 9262 | 2934 | 241 | 1.00 | 96307 | 8911 | 0.34 | 2.6 | 6 | 2 |
| 2003 | 495.20 | 9.58 | 4666 | 1779 | 238 | 8.00 | 96273 | 7288 | 0.24 | 2.6 | 6 | 2 |
| 2004 | 515.50 | 9.43 | 8093 | 3170 | 242 | 9.00 | 96305 | 9167 | 0.36 | 2.6 | 6 | 2 |
| 2005 | 256.20 | 9.40 | 11727 | 5645 | 238 | 1.00 | 96321 | 8584 | 0.48 | 2.6 | 6 | 2 |
| 2006 | 319.70 | 8.77 | 8525 | 4290 | 239 | 2.00 | 96325 | 8388 | 0.34 | 2.6 | 6 | 2 |
| 2007 | 602.70 | 10.18 | 4325 | 1001 | 245 | 8.00 | 96254 | 9368 | 0.26 | 2.6 | 6 | 2 |
| 2008 | 533.00 | 9.35 | 13187 | 4192 | 244 | 5.00 | 96291 | 9562 | 0.42 | 2.6 | 6 | 2 |
| 2009 | 342.00 | 8.85 | 3521 | 1469 | 234 | 6.00 | 96295 | 6959 | 0.25 | 2.6 | 6 | 2 |
| 2010 | 498.20 | 8.53 | 1668 | 718 | 241 | 6.00 | 96280 | 7035 | 0.15 | 2.6 | 6 | 2 |
| 2011 | 337.70 | 8.53 | 4180 | 1181 | 235 | 3.00 | 96297 | 7732 | 0.20 | 2.6 | 6 | 2 |

**Table S12 | Specimen case file data with *test cases of 25% dataset (2012-2022)* simulated by the DSSAT tool for analyzing CT vs NT for the response variable, considering soilscape PDPG 6,7,8 with varying OC levels of 1,3,5,7%. (Full case file with test cases of 25% datasets from 2012-2022 is available separately in the folder of supplementary documentation.)**

| **IDnum** | **PRED** | **TMEAN** | **TWAD** | **HWAD** | **AWS** | **ROFC** | **SOMC** | **GHG** | **PARID** | **SOILSCAPE** | **TILLAGE** |
| --- | --- | --- | --- | --- | --- | --- | --- | --- | --- | --- | --- |
| 2012 | 523.80 | 9.35 | 16987 | 4946 | 246 | 9.00 | 96275 | 10774 | 0.51 | 6 | 2 |
| 2013 | 508.20 | 8.40 | 2753 | 1286 | 239 | 6.00 | 96288 | 7188 | 0.21 | 6 | 2 |
| 2014 | 690.00 | 9.57 | 9636 | 2844 | 247 | 14.00 | 96283 | 9804 | 0.32 | 6 | 2 |
| 2015 | 368.00 | 9.21 | 3465 | 1530 | 232 | 1.00 | 96299 | 7176 | 0.26 | 6 | 2 |
| 2016 | 506.50 | 9.94 | 13034 | 4776 | 240 | 1.00 | 96293 | 9535 | 0.48 | 6 | 2 |
| 2017 | 348.50 | 9.52 | 5145 | 2186 | 233 | 5.00 | 96293 | 7618 | 0.29 | 6 | 2 |
| 2018 | 404.50 | 9.29 | 4683 | 2110 | 240 | 1.00 | 96293 | 7804 | 0.29 | 6 | 2 |
| 2019 | 387.40 | 9.62 | 6352 | 1922 | 233 | 5.00 | 96284 | 8121 | 0.34 | 6 | 2 |
| 2020 | 519.40 | 9.70 | 4253 | 1284 | 239 | 5.00 | 96256 | 7813 | 0.22 | 6 | 2 |
| 2021 | 501.10 | 9.07 | 11967 | 3693 | 240 | 5.00 | 96290 | 9021 | 0.37 | 6 | 2 |
| 2022 | 279.80 | 9.82 | 5988 | 2429 | 227 | 1.00 | 96311 | 7434 | 0.37 | 6 | 2 |

**Enclosure S1. K-Fold Cross Validation**

The K-fold cross-validation test was conducted using the k-folds technique for test splitting. The data spanning from 1975 to 2014 were divided into four equal parts, each representing a 10-year interval. These segments were labeled as training datasets, designated as follows: **T1**=K1(1975-1984)+K2(1985-1994)+K3(1995-2004), **T2**=K1(1975-1984)+K2(1985-1994)+K4(2005-2014), **T3**=K1(1975-1984)+K3(1995-2004)+ **K4=** (2005-2014), and T4=K2(1985-1994)+K3(1995-2004)+K4(2005-2014). The remaining data, spanning from 2015 to 2022, was reserved for testing the model. Each ten-year bracket of K-fold served as a validation dataset, represented as under: **V1=**K4(2005-2014), **V2=**K3(1995-2004), **V3=**K2(1985-1994), and **V4=**K1(1975-1984).

The K-fold cross validation process was executed using Netica software. Following results were retrieved.

1. **K-fold cross validation using “Training dataset (T1) & Validation dataset (V1)”**

Read 300 cases, and used 300 of them to test net.

For HWAD: Crop Yield - (HWAD) kg/ha

--------

Confusion:

...............Predicted..............

very l low medium high very h Actual

------ ------ ------ ------ ------ ------

40 11 1 0 0 very low

2 40 19 1 0 low

0 22 19 25 3 medium

0 0 47 20 24 high

0 0 2 12 12 very high

Testing Real Value:

Absolute error: mean = 863.4 max = 3866 rms = 1072

Relative error: mean = 31.98 % max = 835 %

Error / std dev: mean = 73.1 % max = 331.6 %

Distribution within: 0-1 std dev 1-2 std dev 2-3 std dev >3 std dev

74 % 24.3 % 1.33 % 0.333 %

Error rate = 56.33%

Scoring Rule Results:

Logarithmic loss = 1.119

Quadratic loss = 0.6555

Spherical payoff = 0.5842

Calibration:

very low 0-1: 0 | 1-2: 1.14 | 2-5: 0 | 5-15: 15.1 | 15-85: 95.2 |

low 0-1: 1.72 | 1-5: 0 | 5-15: 16.2 | 15-60: 54.8 |

medium 0-1: 0 | 1-5: 7.69 | 5-20: 43.1 | 20-30: 30.1 | 30-60: 21.6 |

high 0-0.5: 0 | 0.5-1: 0 | 1-25: 61.5 | 25-30: 53.4 | 30-60: 34.5 |

very high 0-0.5: 0 | 0.5-1: 1.54 | 1-30: 20.7 | 30-70: 30.8 |

Total 0-0.5: 0 | 0.5-1: 0.909| 1-2: 1.14 | 2-5: 2.56 | 5-15: 15.8 | 15-20: 43.1 | 20-25: 61.5 | 25-30: 37 | 30-60: 36.1 | 60-70: 30.8 |

70-85: 95.2 |

Times Surprised (percentage):

.................Predicted Probability....................

State < 1% < 10% > 90% > 99%

----- ---- ----- ----- -----

very low 0.00 (0/58) 0.54 (1/185) 0.00 (0/0) 0.00 (0/0)

low 1.72 (1/58) 1.03 (1/97) 0.00 (0/0) 0.00 (0/0)

medium 0.00 (0/42) 3.70 (3/81) 0.00 (0/0) 0.00 (0/0)

high 0.00 (0/115) 0.00 (0/115) 0.00 (0/0) 0.00 (0/0)

very high 0.99 (2/203) 0.99 (2/203) 0.00 (0/0) 0.00 (0/0)

Total 0.63 (3/476) 1.03 (7/681) 0.00 (0/0) 0.00 (0/0)

Sensitivity of Test:

very low 0 100 | 15 76.9 | 85 0 | 100 0 |

low 0 100 | 15 64.5 | 60 0 | 100 0 |

medium 0 100 | 20 59.4 | 30 27.5 | 60 0 | 100 0 |

high 0 100 | 25 73.6 | 30 22 | 60 0 | 100 0 |

very high 0 100 | 30 46.2 | 70 0 | 100 0 |

-------------------------------------------------------------------------------

For ROFC: Runoff - (ROFC) mm

--------

Confusion:

...............Predicted..............

very l low medium high very h Actual

------ ------ ------ ------ ------ ------

135 15 0 0 0 very low

58 48 0 0 0 low

7 24 0 0 0 medium

1 7 0 0 0 high

2 3 0 0 0 very high

Testing Real Value:

Absolute error: mean = 8.146 max = 22.98 rms = 9.895

Error / std dev: mean = 71.23 % max = 223.3 %

Distribution within: 0-1 std dev 1-2 std dev 2-3 std dev >3 std dev

77.3 % 22 % 0.667 % 0 %

Error rate = 39%

Scoring Rule Results:

Logarithmic loss = 1.196

Quadratic loss = 0.6099

Spherical payoff = 0.6202

Calibration:

very low 0-10: 15 | 10-25: 41.3 | 25-50: 51.8 | 50-60: 66.7 | 60-70: 67.5 | 70-100: 76.2 |

low 0-15: 26.3 | 15-25: 18.8 | 25-40: 45.1 | 40-60: 54.5 |

medium 0-10: 2.27 | 10-15: 0 | 15-25: 18.4 | 25-30: 22.8 |

high 0-10: 0.658| 10-15: 2.99 | 15-25: 6.17 |

very high 0-5: 0 | 5-10: 0.885| 10-15: 2.74 | 15-25: 3.51 |

Total 0-5: 3.6 | 5-10: 2.95 | 10-15: 8.93 | 15-20: 19.1 | 20-25: 20 | 25-30: 33.7 | 30-40: 46.8 | 40-50: 44.4 | 50-60: 65.6 | 60-70: 67.5 |

70-100: 76.2 |

Times Surprised (percentage):

.................Predicted Probability....................

State < 1% < 10% > 90% > 99%

----- ---- ----- ----- -----

very low 0.00 (0/0) 15.00 (6/40) 0.00 (0/0) 0.00 (0/0)

low 0.00 (0/0) 14.71 (5/34) 0.00 (0/0) 0.00 (0/0)

medium 0.00 (0/0) 2.27 (2/88) 0.00 (0/0) 0.00 (0/0)

high 0.00 (0/0) 0.66 (1/152) 0.00 (0/0) 0.00 (0/0)

very high 0.00 (0/0) 0.59 (1/170) 0.00 (0/0) 0.00 (0/0)

Total 0.00 (0/0) 3.10 (15/484) 0.00 (0/0) 0.00 (0/0)

Sensitivity of Test:

very low 0 100 | 25 70.7 | 40 60 | 50 51.3 | 60 28.7 | 70 10.7 | 80 3.33 | 100 0 |

low 0 100 | 15 75.5 | 25 64.2 | 30 50.9 | 40 34 | 50 23.6 | 60 0 | 100 0 |

medium 0 100 | 25 41.9 | 30 0 | 100 0 |

-------------------------------------------------------------------------------

For GHG: Total GHG - CO2 Emission (Kg/h

-------

Confusion:

...............Predicted..............

very l low medium high very h Actual

------ ------ ------ ------ ------ ------

205 5 0 0 0 very low

59 2 0 0 0 low

21 1 0 0 0 medium

4 0 0 0 0 high

3 0 0 0 0 very high

Testing Real Value:

Absolute error: mean = 6091 max = 1.566e+04 rms = 6968

Relative error: mean = 101.4 % max = 445.7 %

Error / std dev: mean = 70.91 % max = 176.4 %

Distribution within: 0-1 std dev 1-2 std dev 2-3 std dev >3 std dev

78 % 22 % 0 % 0 %

Error rate = 31%

Scoring Rule Results:

Logarithmic loss = 1.049

Quadratic loss = 0.5256

Spherical payoff = 0.6824

Calibration:

very low 0-25: 54.2 | 25-40: 55.4 | 40-60: 73.1 | 60-70: 86.4 | 70-100: 100 |

low 0-15: 19 | 15-25: 20.8 | 25-30: 15.2 | 30-40: 24.5 | 40-100: 50 |

medium 0-5: 0 | 5-10: 6.17 | 10-15: 4.76 | 15-25: 8.45 | 25-100: 31.8 |

high 0-5: 0 | 5-10: 1.25 | 10-15: 0 | 15-25: 3.33 | 25-100: 14.3 |

very high 0-5: 0 | 5-10: 1 | 10-15: 3.08 | 15-25: 0 |

Total 0-5: 0 | 5-10: 4.51 | 10-15: 5.73 | 15-20: 12 | 20-25: 18.5 | 25-30: 18.8 | 30-40: 43.4 | 40-60: 72.6 | 60-70: 86.4 | 70-100: 100 |

Times Surprised (percentage):

.................Predicted Probability....................

State < 1% < 10% > 90% > 99%

----- ---- ----- ----- -----

very low 0.00 (0/0) 0.00 (0/0) 0.00 (0/0) 0.00 (0/0)

low 0.00 (0/0) 20.69 (6/29) 0.00 (0/0) 0.00 (0/0)

medium 0.00 (0/0) 4.07 (5/123) 0.00 (0/0) 0.00 (0/0)

high 0.00 (0/0) 0.65 (1/155) 0.00 (0/0) 0.00 (0/0)

very high 0.00 (0/0) 0.56 (1/179) 0.00 (0/0) 0.00 (0/0)

Total 0.00 (0/0) 2.67 (13/486) 0.00 (0/0) 0.00 (0/0)

Sensitivity of Test:

very low 0 100 | 25 84.8 | 40 67.6 | 50 57.1 | 60 35.2 | 70 8.1 | 80 0.952 | 100 0 |

low 0 100 | 15 75.4 | 25 34.4 | 40 1.64 | 100 0 |

medium 0 100 | 15 59.1 | 30 13.6 | 100 0 |

-------------------------------------------------------------------------------.

1. **K-fold cross validation using “Training dataset (T2) & Validation dataset (V2)”**

Read 300 cases, and used 300 of them to test net.

For HWAD: Crop Yield - (HWAD) kg/ha

--------

Confusion:

...............Predicted..............

very l low medium high very h Actual

------ ------ ------ ------ ------ ------

49 4 1 0 0 very low

6 55 32 0 0 low

0 14 54 20 0 medium

0 0 18 25 5 high

0 0 0 9 8 very high

Testing Real Value:

Absolute error: mean = 785.1 max = 2882 rms = 934

Relative error: mean = 68.64 % max = 646.7 %

Error / std dev: mean = 65.66 % max = 239 %

Distribution within: 0-1 std dev 1-2 std dev 2-3 std dev >3 std dev

80.3 % 19.3 % 0.333 % 0 %

Error rate = 36.33%

Scoring Rule Results:

Logarithmic loss = 0.9493

Quadratic loss = 0.5356

Spherical payoff = 0.6733

Calibration:

very low 0-1: 0 | 1-2: 0.847| 2-20: 5.48 | 20-90: 89.1 |

low 0-2: 0 | 2-15: 23.8 | 15-60: 75.3 |

medium 0-2: 0 | 2-20: 29.9 | 20-30: 19.2 | 30-50: 51.4 |

high 0-0.5: 0 | 0.5-2: 0 | 2-40: 17.1 | 40-60: 44.8 |

very high 0-0.5: 0 | 0.5-2: 0 | 2-30: 16.7 | 30-100: 61.5 |

Total 0-0.5: 0 | 0.5-1: 0 | 1-2: 0.22 | 2-15: 22 | 15-20: 18.9 | 20-30: 18.1 | 30-40: 17.1 | 40-50: 51.1 | 50-60: 63 | 60-90: 89.1 |

Times Surprised (percentage):

.................Predicted Probability....................

State < 1% < 10% > 90% > 99%

----- ---- ----- ----- -----

very low 0.00 (0/54) 0.58 (1/172) 0.00 (0/0) 0.00 (0/0)

low 0.00 (0/0) 0.00 (0/67) 0.00 (0/0) 0.00 (0/0)

medium 0.00 (0/0) 0.00 (0/68) 0.00 (0/0) 0.00 (0/0)

high 0.00 (0/73) 0.00 (0/128) 0.00 (0/0) 0.00 (0/0)

very high 0.00 (0/73) 0.00 (0/233) 0.00 (0/0) 0.00 (0/0)

Total 0.00 (0/200) 0.15 (1/668) 0.00 (0/0) 0.00 (0/0)

Sensitivity of Test:

very low 0 100 | 90 0 | 100 0 |

low 0 100 | 15 59.1 | 60 0 | 100 0 |

medium 0 100 | 20 77.3 | 30 61.4 | 50 0 | 100 0 |

high 0 100 | 40 62.5 | 60 0 | 100 0 |

very high 0 100 | 30 47.1 | 50 0 | 100 0 |

-------------------------------------------------------------------------------

For ROFC: Runoff - (ROFC) mm

--------

Confusion:

...............Predicted..............

very l low medium high very h Actual

------ ------ ------ ------ ------ ------

109 16 0 0 0 very low

73 47 3 0 0 low

22 17 2 0 0 medium

6 3 1 0 0 high

1 0 0 0 0 very high

Testing Real Value:

Absolute error: mean = 5.736 max = 22.18 rms = 6.925

Error / std dev: mean = 57.44 % max = 269.2 %

Distribution within: 0-1 std dev 1-2 std dev 2-3 std dev >3 std dev

90.3 % 9.33 % 0.333 % 0 %

Error rate = 47.33%

Scoring Rule Results:

Logarithmic loss = 1.122

Quadratic loss = 0.6062

Spherical payoff = 0.6277

Calibration:

very low 0-25: 5.88 | 25-40: 39.4 | 40-50: 55.6 | 50-70: 45 | 70-80: 66.7 | 80-100: 85.7 |

low 0-15: 23.3 | 15-25: 43.4 | 25-30: 45 | 30-40: 40.4 | 40-50: 52.6 | 50-100: 58.6 |

medium 0-5: 5.41 | 5-10: 7.69 | 10-15: 8.7 | 15-25: 25.6 | 25-100: 33.3 |

high 0-5: 0.98 | 5-10: 1.65 | 10-15: 2.44 | 15-30: 16.7 |

very high 0-2: 0 | 2-5: 0 | 5-10: 1.39 | 10-15: 0 | 15-100: 0 |

Total 0-2: 0 | 2-5: 1.92 | 5-10: 4.04 | 10-15: 9.39 | 15-20: 13.3 | 20-25: 26 | 25-30: 35.8 | 30-40: 44.9 | 40-50: 54.1 | 50-70: 49.4 |

70-80: 66.7 | 80-100: 85.7 |

Times Surprised (percentage):

.................Predicted Probability....................

State < 1% < 10% > 90% > 99%

----- ---- ----- ----- -----

very low 0.00 (0/0) 0.00 (0/10) 0.00 (0/0) 0.00 (0/0)

low 0.00 (0/0) 18.75 (3/16) 0.00 (0/0) 0.00 (0/0)

medium 0.00 (0/0) 6.58 (10/152) 0.00 (0/0) 0.00 (0/0)

high 0.00 (0/0) 1.35 (3/223) 0.00 (0/0) 0.00 (0/0)

very high 0.00 (0/0) 0.45 (1/223) 0.00 (0/0) 0.00 (0/0)

Total 0.00 (0/0) 2.72 (17/624) 0.00 (0/0) 0.00 (0/0)

Sensitivity of Test:

very low 0 100 | 30 90.4 | 40 74.4 | 50 58.4 | 70 36.8 | 80 9.6 | 85 0 | 100 0 |

low 0 100 | 15 86.2 | 25 67.5 | 30 45.5 | 40 30.1 | 50 13.8 | 60 0 | 100 0 |

medium 0 100 | 10 75.6 | 20 53.7 | 25 9.76 | 100 0 |

high 0 100 | 25 10 | 100 0 |

-------------------------------------------------------------------------------

For GHG: Total GHG - CO2 Emission (Kg/h

-------

Confusion:

...............Predicted..............

very l low medium high very h Actual

------ ------ ------ ------ ------ ------

191 1 0 0 0 very low

73 0 0 0 0 low

20 1 0 0 0 medium

11 0 0 0 0 high

3 0 0 0 0 very high

Testing Real Value:

Absolute error: mean = 5474 max = 1.627e+04 rms = 6384

Relative error: mean = 83.71 % max = 488 %

Error / std dev: mean = 63.76 % max = 173.2 %

Distribution within: 0-1 std dev 1-2 std dev 2-3 std dev >3 std dev

85.7 % 14.3 % 0 % 0 %

Error rate = 36.33%

Scoring Rule Results:

Logarithmic loss = 1.089

Quadratic loss = 0.551

Spherical payoff = 0.6646

Calibration:

very low 0-30: 42.4 | 30-50: 53.3 | 50-60: 76.1 | 60-70: 71.7 | 70-80: 84.8 | 80-100: 57.1 |

low 0-10: 22.9 | 10-15: 21.8 | 15-25: 29.4 | 25-30: 25 | 30-100: 19.4 |

medium 0-10: 3.74 | 10-15: 8.11 | 15-25: 6.78 | 25-100: 17.4 |

high 0-5: 0 | 5-10: 0.98 | 10-15: 4.94 | 15-25: 10.3 | 25-100: 18.2 |

very high 0-5: 0 | 5-10: 0.833| 10-15: 1.27 | 15-100: 2.94 |

Total 0-5: 0 | 5-10: 4.39 | 10-15: 9.68 | 15-20: 12.2 | 20-25: 18.7 | 25-30: 29.5 | 30-40: 29.8 | 40-50: 54.2 | 50-60: 76.1 | 60-70: 71.7 |

70-80: 84.8 | 80-100: 57.1 |

Times Surprised (percentage):

.................Predicted Probability....................

State < 1% < 10% > 90% > 99%

----- ---- ----- ----- -----

very low 0.00 (0/0) 50.00 (1/2) 0.00 (0/0) 0.00 (0/0)

low 0.00 (0/0) 22.86 (8/35) 0.00 (0/0) 0.00 (0/0)

medium 0.00 (0/0) 3.74 (4/107) 0.00 (0/0) 0.00 (0/0)

high 0.00 (0/0) 0.59 (1/169) 0.00 (0/0) 0.00 (0/0)

very high 0.00 (0/0) 0.53 (1/187) 0.00 (0/0) 0.00 (0/0)

Total 0.00 (0/0) 3.00 (15/500) 0.00 (0/0) 0.00 (0/0)

Sensitivity of Test:

very low 0 100 | 25 94.8 | 30 87 | 40 82.8 | 50 66.1 | 60 39.6 | 70 22.4 | 75 10.4 | 80 2.08 | 100 0 |

low 0 100 | 10 89 | 15 58.9 | 25 24.7 | 30 8.22 | 100 0 |

medium 0 100 | 15 38.1 | 30 0 | 100 0 |

high 0 100 | 25 18.2 | 100 0 |

1. **K-fold cross validation test using “Training dataset (T3) & Validation dataset (V3)”**

Read 300 cases, and used 300 of them to test net.

For HWAD: Crop Yield - (HWAD) kg/ha

--------

Confusion:

...............Predicted..............

very l low medium high very h Actual

------ ------ ------ ------ ------ ------

21 0 0 0 0 very low

2 43 3 0 0 low

0 21 73 6 0 medium

0 0 42 43 0 high

0 0 0 46 0 very high

Testing Real Value:

Absolute error: mean = 756.2 max = 2615 rms = 943.1

Relative error: mean = 18.82 % max = 286.5 %

Error / std dev: mean = 59.98 % max = 206.4 %

Distribution within: 0-1 std dev 1-2 std dev 2-3 std dev >3 std dev

80.3 % 19.3 % 0.333 % 0 %

Error rate = 40%

Scoring Rule Results:

Logarithmic loss = 0.9189

Quadratic loss = 0.5382

Spherical payoff = 0.6778

Calibration:

very low 0-1: 0 | 1-2: 0 | 2-20: 0 | 20-100: 91.3 |

low 0-2: 0 | 2-20: 3.55 | 20-60: 67.2 |

medium 0-10: 0 | 10-25: 32.8 | 25-30: 7.79 | 30-50: 61.9 |

high 0-0.5: 0 | 0.5-40: 29.8 | 40-60: 45.3 |

very high 0-0.5: 0 | 0.5-2: 0 | 2-20: 40.3 | 20-100: 83.3 |

Total 0-0.5: 0 | 0.5-1: 0 | 1-2: 0 | 2-15: 4.88 | 15-20: 13.1 | 20-25: 32.8 | 25-30: 7.79 | 30-40: 41.9 | 40-50: 61.9 | 50-60: 54.1 |

60-100: 91.3 |

Times Surprised (percentage):

.................Predicted Probability....................

State < 1% < 10% > 90% > 99%

----- ---- ----- ----- -----

very low 0.00 (0/77) 0.00 (0/213) 0.00 (0/0) 0.00 (0/0)

low 0.00 (0/0) 0.00 (0/95) 0.00 (0/0) 0.00 (0/0)

medium 0.00 (0/23) 0.00 (0/41) 0.00 (0/0) 0.00 (0/0)

high 0.00 (0/87) 0.00 (0/87) 0.00 (0/0) 0.00 (0/0)

very high 0.00 (0/87) 0.00 (0/205) 0.00 (0/0) 0.00 (0/0)

Total 0.00 (0/274) 0.00 (0/641) 0.00 (0/0) 0.00 (0/0)

Sensitivity of Test:

very low 0 100 | 90 0 | 100 0 |

low 0 100 | 60 0 | 100 0 |

medium 0 100 | 25 79 | 50 0 | 100 0 |

high 0 100 | 40 50.6 | 60 0 | 100 0 |

very high 0 100 | 20 32.6 | 40 0 | 100 0 |

-------------------------------------------------------------------------------

For ROFC: Runoff - (ROFC) mm

--------

Confusion:

...............Predicted..............

very l low medium high very h Actual

------ ------ ------ ------ ------ ------

192 31 0 0 0 very low

37 19 0 0 0 low

8 6 0 0 0 medium

4 3 0 0 0 high

0 0 0 0 0 very high

Testing Real Value:

Absolute error: mean = 8.024 max = 24.13 rms = 9.674

Error / std dev: mean = 81.22 % max = 288.1 %

Distribution within: 0-1 std dev 1-2 std dev 2-3 std dev >3 std dev

68.3 % 29.3 % 2.33 % 0 %

Error rate = 29.67%

Scoring Rule Results:

Logarithmic loss = 0.9444

Quadratic loss = 0.4874

Spherical payoff = 0.7167

Calibration:

very low 0-30: 61.9 | 30-50: 67 | 50-60: 75.4 | 60-70: 88.1 | 70-75: 83.9 | 75-100: 100 |

low 0-20: 10.5 | 20-25: 10.5 | 25-40: 21.8 | 40-50: 25.3 | 50-100: 70 |

medium 0-5: 5.94 | 5-10: 3.97 | 10-20: 0 | 20-100: 10 |

high 0-5: 3.54 | 5-10: 0 | 10-15: 0 | 15-100: 0 |

very high 0-5: 0 | 5-15: 0 | 15-100: 0 |

Total 0-5: 2.5 | 5-10: 2.45 | 10-15: 5.74 | 15-20: 9.02 | 20-25: 18.3 | 25-40: 38.2 | 40-50: 43.7 | 50-60: 74.6 | 60-70: 88.1 | 70-75: 83.9 |

75-100: 100 |

Times Surprised (percentage):

.................Predicted Probability....................

State < 1% < 10% > 90% > 99%

----- ---- ----- ----- -----

very low 0.00 (0/0) 0.00 (0/0) 0.00 (0/0) 0.00 (0/0)

low 0.00 (0/0) 0.00 (0/4) 0.00 (0/0) 0.00 (0/0)

medium 0.00 (0/0) 4.85 (11/227) 0.00 (0/0) 0.00 (0/0)

high 0.00 (0/0) 2.88 (7/243) 0.00 (0/0) 0.00 (0/0)

very high 0.00 (0/0) 0.00 (0/251) 0.00 (0/0) 0.00 (0/0)

Total 0.00 (0/0) 2.48 (18/725) 0.00 (0/0) 0.00 (0/0)

Sensitivity of Test:

very low 0 100 | 25 91.9 | 30 88.3 | 40 82.5 | 50 59.2 | 60 38.6 | 70 22 | 75 0.897 | 100 0 |

low 0 100 | 20 78.6 | 40 50 | 50 12.5 | 100 0 |

medium 0 100 | 10 21.4 | 100 0 |

-------------------------------------------------------------------------------

For GHG: Total GHG - CO2 Emission (Kg/h

-------

Confusion:

...............Predicted..............

very l low medium high very h Actual

------ ------ ------ ------ ------ ------

217 3 0 0 0 very low

52 1 0 0 0 low

18 0 0 0 0 medium

9 0 0 0 0 high

0 0 0 0 0 very high

Testing Real Value:

Absolute error: mean = 5309 max = 1.429e+04 rms = 6171

Relative error: mean = 91.81 % max = 569.2 %

Error / std dev: mean = 63.67 % max = 152.1 %

Distribution within: 0-1 std dev 1-2 std dev 2-3 std dev >3 std dev

86.7 % 13.3 % 0 % 0 %

Error rate = 27.33%

Scoring Rule Results:

Logarithmic loss = 0.927

Quadratic loss = 0.4764

Spherical payoff = 0.7229

Calibration:

very low 0-30: 65.8 | 30-50: 63.5 | 50-60: 72 | 60-70: 78 | 70-100: 96.8 |

low 0-15: 5.45 | 15-20: 15.2 | 20-25: 18.1 | 25-30: 24.6 | 30-100: 46.2 |

medium 0-5: 0 | 5-10: 2.38 | 10-15: 3.7 | 15-25: 12.5 | 25-100: 40 |

high 0-5: 0 | 5-10: 0 | 10-20: 2 | 20-25: 18.2 |

very high 0-5: 0 | 5-10: 0 | 10-20: 0 | 20-100: 0 |

Total 0-5: 0 | 5-10: 0.557| 10-15: 4.65 | 15-20: 11.6 | 20-25: 19.4 | 25-30: 31.7 | 30-40: 56.8 | 40-50: 65.1 | 50-60: 72 | 60-70: 78 |

70-100: 96.8 |

Times Surprised (percentage):

.................Predicted Probability....................

State < 1% < 10% > 90% > 99%

----- ---- ----- ----- -----

very low 0.00 (0/0) 0.00 (0/0) 0.00 (0/0) 0.00 (0/0)

low 0.00 (0/0) 0.00 (0/28) 0.00 (0/0) 0.00 (0/0)

medium 0.00 (0/0) 1.59 (2/126) 0.00 (0/0) 0.00 (0/0)

high 0.00 (0/0) 0.00 (0/206) 0.00 (0/0) 0.00 (0/0)

very high 0.00 (0/0) 0.00 (0/231) 0.00 (0/0) 0.00 (0/0)

Total 0.00 (0/0) 0.34 (2/591) 0.00 (0/0) 0.00 (0/0)

Sensitivity of Test:

very low 0 100 | 25 92.7 | 30 88.6 | 40 79.5 | 50 67.3 | 60 42.7 | 70 13.6 | 75 8.64 | 80 0 | 100 0 |

low 0 100 | 20 75.5 | 25 39.6 | 30 11.3 | 100 0 |

medium 0 100 | 20 44.4 | 30 0 | 100 0 |

high 0 100 | 25 0 | 100 0 |

1. **K-fold cross validation test using “Training dataset (T4) & Validation dataset (V4)”**

Read 300 cases, and used 300 of them to test net.

For HWAD: Crop Yield - (HWAD) kg/ha

Confusion:

...............Predicted..............

very l low medium high very h Actual

------ ------ ------ ------ ------ ------

18 32 2 0 0 very low

6 48 10 0 0 low

0 26 70 4 0 medium

0 0 34 38 0 high

0 0 1 9 2 very high

Testing Real Value:

Absolute error: mean = 747.4 max = 3415 rms = 944.1

Relative error: mean = 31.84 % max = 351 %

Error / std dev: mean = 64.65 % max = 268.7 %

Distribution within: 0-1 std dev 1-2 std dev 2-3 std dev >3 std dev

75 % 24 % 1 % 0 %

Error rate = 41.33%

Scoring Rule Results:

Logarithmic loss = 1.086

Quadratic loss = 0.6139

Spherical payoff = 0.6278

Calibration:

very low 0-1: 1.19 | 1-10: 29.6 | 10-100: 75 |

low 0-2: 0 | 2-20: 11.3 | 20-70: 45.3 |

medium 0-30: 16.4 | 30-50: 59.8 |

high 0-0.5: 0 | 0.5-40: 24.1 | 40-50: 71.7 |

very high 0-0.5: 0 | 0.5-1: 0.709| 1-30: 17.6 | 30-100: 100 |

Total 0-0.5: 0 | 0.5-1: 0.84 | 1-2: 0 | 2-10: 28.8 | 10-20: 8.55 | 20-30: 18.8 | 30-40: 29.1 | 40-50: 64 | 50-70: 45.3 | 70-100: 75 |

Times Surprised (percentage):

.................Predicted Probability....................

State < 1% < 10% > 90% > 99%

----- ---- ----- ----- -----

very low 1.19 (2/168) 12.32 (34/276) 0.00 (0/0) 0.00 (0/0)

low 0.00 (0/0) 7.79 (6/77) 0.00 (0/0) 0.00 (0/0)

medium 0.00 (0/24) 0.00 (0/26) 0.00 (0/0) 0.00 (0/0)

high 0.00 (0/130) 0.00 (0/130) 0.00 (0/0) 0.00 (0/0)

very high 0.40 (1/247) 0.40 (1/247) 0.00 (0/0) 0.00 (0/0)

Total 0.53 (3/569) 5.42 (41/756) 0.00 (0/0) 0.00 (0/0)

Sensitivity of Test:

very low 0 100 | 10 34.6 | 90 0 | 100 0 |

low 0 100 | 20 75 | 70 0 | 100 0 |

medium 0 100 | 30 70 | 50 0 | 100 0 |

high 0 100 | 40 52.8 | 50 0 | 100 0 |

very high 0 100 | 30 16.7 | 100 0 |

------------------------------------------------------------------------------

For ROFC: Runoff - (ROFC) mm

--------

Confusion:

...............Predicted..............

very l low medium high very h Actual

------ ------ ------ ------ ------ ------

158 27 0 0 0 very low

54 34 0 0 0 low

6 16 0 0 0 medium

0 5 0 0 0 high

0 0 0 0 0 very high

Testing Real Value:

Absolute error: mean = 7.269 max = 21 rms = 8.938

Error / std dev: mean = 71.81 % max = 201.5 %

Distribution within: 0-1 std dev 1-2 std dev 2-3 std dev >3 std dev

72 % 27.7 % 0.333 % 0 %

Error rate = 36%

Scoring Rule Results:

Logarithmic loss = 1.066

Quadratic loss = 0.568

Spherical payoff = 0.6506

Calibration:

very low 0-20: 27 | 20-25: 61.5 | 25-50: 66.1 | 50-60: 54.4 | 60-70: 82.5 | 70-100: 80 |

low 0-20: 10.9 | 20-25: 20.8 | 25-30: 32.5 | 30-40: 37 | 40-60: 40.9 | 60-100: 50 |

medium 0-10: 3.25 | 10-15: 1.72 | 15-20: 11.1 | 20-25: 10 | 25-100: 24.2 |

high 0-5: 1.06 | 5-10: 2.56 | 10-15: 0 | 15-25: 2.13 |

very high 0-5: 0 | 5-10: 0 | 10-25: 0 |

Total 0-2: 0 | 2-5: 0.725| 5-10: 3.3 | 10-15: 5.44 | 15-20: 11.8 | 20-25: 25.6 | 25-30: 28.8 | 30-40: 41.6 | 40-50: 54.2 | 50-60: 51.3 |

60-70: 79.4 | 70-100: 80 |

Times Surprised (percentage):

.................Predicted Probability....................

State < 1% < 10% > 90% > 99%

----- ---- ----- ----- -----

very low 0.00 (0/0) 15.00 (3/20) 0.00 (0/0) 0.00 (0/0)

low 0.00 (0/0) 0.00 (0/0) 0.00 (0/0) 0.00 (0/0)

medium 0.00 (0/0) 3.25 (4/123) 0.00 (0/0) 0.00 (0/0)

high 0.00 (0/0) 1.90 (4/211) 0.00 (0/0) 0.00 (0/0)

very high 0.00 (0/0) 0.00 (0/248) 0.00 (0/0) 0.00 (0/0)

Total 0.00 (0/0) 1.83 (11/602) 0.00 (0/0) 0.00 (0/0)

Sensitivity of Test:

very low 0 100 | 20 94.6 | 25 68.6 | 40 64.3 | 50 48.6 | 60 31.9 | 70 6.49 | 85 0 | 100 0 |

low 0 100 | 25 77.3 | 30 62.5 | 40 23.9 | 50 12.5 | 60 3.41 | 100 0 |

medium 0 100 | 20 59.1 | 30 0 | 100 0 |

-------------------------------------------------------------------------------

For GHG: Total GHG - CO2 Emission (Kg/h

Confusion:

...............Predicted..............

very l low medium high very h Actual

------ ------ ------ ------ ------ ------

205 4 0 0 0 very low

60 2 0 0 0 low

20 1 0 0 0 medium

6 1 0 0 0 high

1 0 0 0 0 very high

Testing Real Value:

Absolute error: mean = 5244 max = 1.816e+04 rms = 5982

Relative error: mean = 89.87 % max = 412 %

Error / std dev: mean = 61.5 % max = 193.2 %

Distribution within: 0-1 std dev 1-2 std dev 2-3 std dev >3 std dev

91.3 % 8.67 % 0 % 0 %

Error rate = 31%

Scoring Rule Results:

Logarithmic loss = 0.9504

Quadratic loss = 0.4828

Spherical payoff = 0.72

Calibration:

very low 0-40: 50.8 | 40-60: 65.6 | 60-70: 83.1 | 70-80: 87.2 |

low 0-10: 9.62 | 10-15: 16.3 | 15-25: 21.4 | 25-30: 31.1 | 30-50: 30.8 |

medium 0-10: 4.76 | 10-15: 4.17 | 15-25: 16.3 | 25-100: 17.6 |

high 0-5: 0 | 5-10: 0.633| 10-15: 2.78 | 15-100: 18.2 |

very high 0-5: 0 | 5-10: 0 | 10-15: 1.2 | 15-100: 0 |

Total 0-5: 0 | 5-10: 2.69 | 10-15: 6.19 | 15-20: 12.5 | 20-25: 23 | 25-30: 28.6 | 30-40: 43.2 | 40-50: 57.1 | 50-60: 65.4 | 60-70: 83.1 |

70-80: 87.2 |

Times Surprised (percentage):

.................Predicted Probability....................

State < 1% < 10% > 90% > 99%

----- ---- ----- ----- -----

very low 0.00 (0/0) 0.00 (0/0) 0.00 (0/0) 0.00 (0/0)

low 0.00 (0/0) 9.62 (5/52) 0.00 (0/0) 0.00 (0/0)

medium 0.00 (0/0) 4.76 (8/168) 0.00 (0/0) 0.00 (0/0)

high 0.00 (0/0) 0.49 (1/206) 0.00 (0/0) 0.00 (0/0)

very high 0.00 (0/0) 0.00 (0/212) 0.00 (0/0) 0.00 (0/0)

Total 0.00 (0/0) 2.19 (14/638) 0.00 (0/0) 0.00 (0/0)

Sensitivity of Test:

very low 0 100 | 40 85.6 | 50 77 | 60 44.5 | 70 16.3 | 75 12.4 | 80 0 | 100 0 |

low 0 100 | 15 71 | 25 41.9 | 30 19.4 | 40 3.23 | 100 0 |

medium 0 100 | 10 61.9 | 25 14.3 | 100 0 |

-------------------------------------------------------------------------------

**Enclosure S2. Multiple Linear Regression Model (Statistical Analysis)**

Below is a multiple linear regression analysis performed on the same datasets used to parametrize the Bayesian Belief Network model. This technique is very common and known as frequentist approach. The model parameters are characterized by by numerical and continuous data types which are used for parametrizing the model.

Hence, all parameters with quantitative or numerical datasets (*e.g.*, 1440 cases) were used in model parameterization.

Model parameters were discretized and coded as per Table S8. Multiple linear regression was applied using the DATAtab software for each output variable of interest, *e.g.*, GHG- CO_2_ emission (GHG), runoff (ROFC), and yield (HWAD).

In order to compute the variable output responses, each of them was used as dependent variable and applied with one reference category of tillage (TILLAGE) which represented code 0 for No-till (NT) and 2 for Conventional till (CT). And the followings were the independent variables used Rainfall (PRED), Temperature (TMEAN), Carbon-Soil organic carbon (SOMA), Available water space (AWS), Precipitation interception (PARID), Biomass (TWAD).

For performing statistical analyses, model parameters with continuous datasets were discretized into segment-wise appropriate ordinal classes/ categories and the process is called discretization (Table S8).

1.
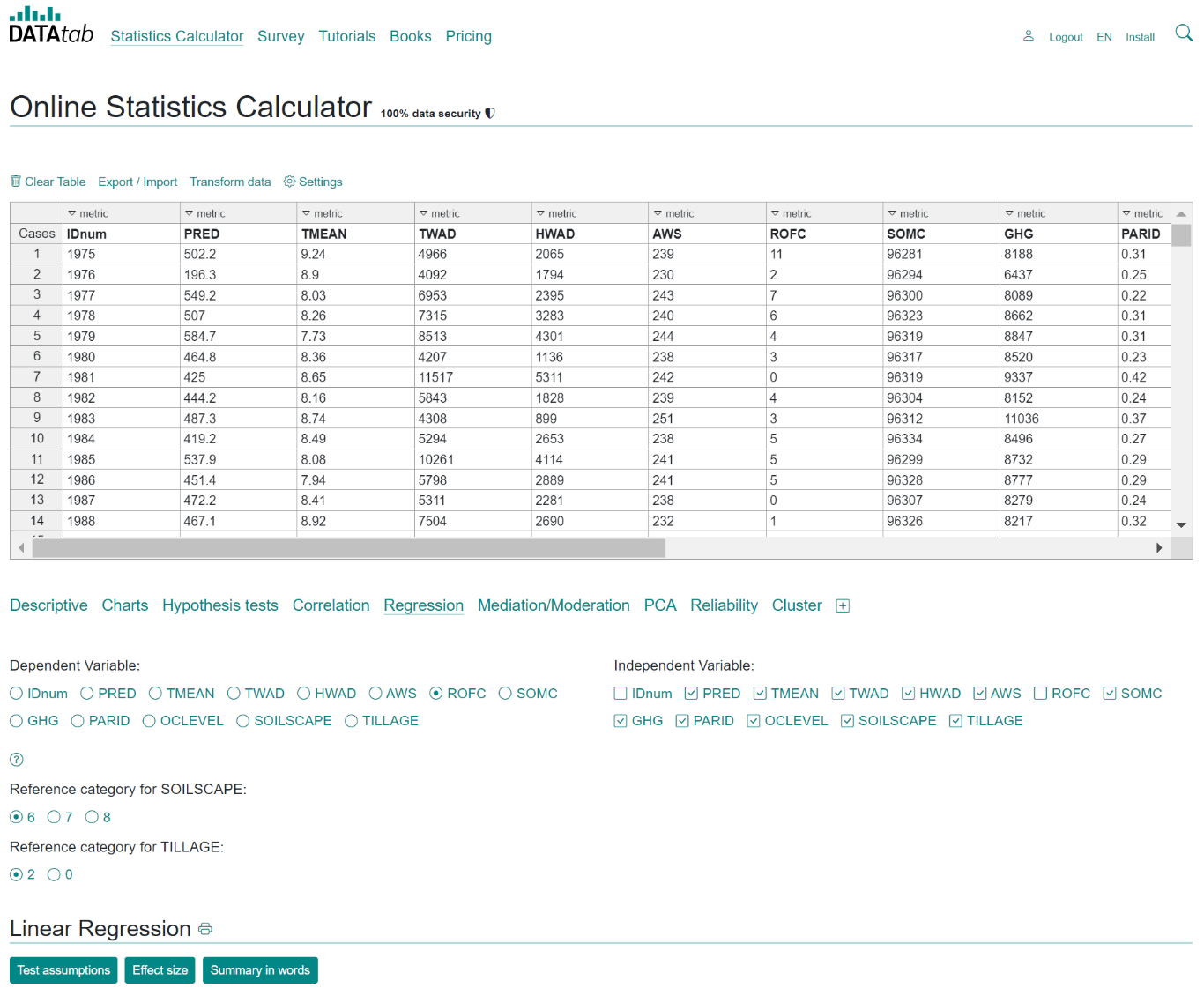
**A multiple linear regression analysis was performed to examine the influence of the variables *PRED, TMEAN, TWAD, HWAD, AWS, SOMC, GHG, PARID, OCLEVEL, SOILSCAPE 7, SOILSCAPE 8 and TILLAGE 0* on the variable *ROFC*.**

### Summary

#### Model Summary

The regression model showed that the variables *PRED, TMEAN, TWAD, HWAD, AWS, SOMC, GHG, PARID, OCLEVEL, SOILSCAPE 7, SOILSCAPE 8 and TILLAGE 0* explained 64.12% of the variance from the variable *ROFC*. An ANOVA was used to test whether this value was significantly different from zero. Using the present sample, it was found that the effect was significantly different from zero, *F*=212.55, *p* = <.001, *R^2^* = 0.64.

#### Regression coefficients

The following regression model is obtained:

ROFC = -48.19 + 0.03 · PRED + 1.83 · TMEAN - 0 · TWAD - 0 · HWAD + 0.11 · AWS - 0 · SOMC + 0 · GHG + 0.91 · PARID + 0.49 · OCLEVEL + 14.49 · SOILSCAPE 7 - 16.1 · SOILSCAPE 8 - 3.22 · TILLAGE 0

- ***Constant*:**When all independent variables are equal to zero, the value of the variable *ROFC* is -48.19.
- ***PRED*:**If the value of the variable *PRED* changes by one unit, the value of the variable *ROFC* changes by 0.03.
- ***TMEAN*:**If the value of the variable *TMEAN* changes by one unit, the value of the variable *ROFC* changes by 1.83.
- ***TWAD*:**If the value of the variable *TWAD* changes by one unit, the value of the variable *ROFC* changes by 0.
- ***HWAD*:**If the value of the variable *HWAD* changes by one unit, the value of the variable *ROFC* changes by 0.
- ***AWS*:**If the value of the variable *AWS* changes by one unit, the value of the variable *ROFC* changes by 0.11.
- ***SOMC*:**If the value of the variable *SOMC* changes by one unit, the value of the variable *ROFC* changes by 0.
- ***GHG*:**If the value of the variable *GHG* changes by one unit, the value of the variable *ROFC* changes by 0.
- ***PARID*:**If the value of the variable *PARID* changes by one unit, the value of the variable *ROFC* changes by 0.91.
- ***OCLEVEL*:**If the value of the variable *OCLEVEL* changes by one unit, the value of the variable *ROFC* changes by 0.49.
- ***SOILSCAPE 7*:**If the value of the variable *SOILSCAPE 7* changes by one unit, the value of the variable *ROFC* changes by 14.49.
- ***SOILSCAPE 8*:**If the value of the variable *SOILSCAPE 8* changes by one unit, the value of the variable *ROFC* changes by -16.1.
- ***TILLAGE 0*:**If the value of the variable *TILLAGE 0* changes by one unit, the value of the variable *ROFC* changes by -3.22.

#### Standardized regression coefficients

The standardized coefficients beta are independent of the measured variable and are always between -1 and 1. The larger the amount of beta, the greater the contribution of the respective independent variable to explain the dependent variable *ROFC*. In this model, the variable *"AWS"* has the greatest influence on the variable *ROFC*.

#### p-value

The calculated regression coefficients refer to the sample used for the calculation of the regression analysis, therefore it is of interest whether the individual coefficients only deviate from zero by chance or whether they also deviate from zero in the population. To test this, the null hypothesis was made for each coefficient that was equal to zero in the population.

The standard error now indicates how much the respective coefficient will scatter on average when the regression analysis is calculated for a further sample. The test statistic t is then calculated from the standard error and the coefficient.

***PRED*:** The p-value for the coefficient of *PRED* was <.001. Thus, the p-value is smaller than the significance level of 0.05 and the null hypothesis that the coefficient of *PRED* is zero in the population is rejected. Thus, it is assumed that the coefficient for the variable *PRED* in the population is different from zero.

***TMEAN*:** The p-value for the coefficient of *TMEAN* was <.001. Thus, the p-value is smaller than the significance level of 0.05 and the null hypothesis that the coefficient of *TMEAN* is zero in the population is rejected. Thus, it is assumed that the coefficient for the variable *TMEAN* in the population is different from zero.

***TWAD*:** The p-value for the coefficient of *TWAD* was <.001. Thus, the p-value is smaller than the significance level of 0.05 and the null hypothesis that the coefficient of *TWAD* is zero in the population is rejected. Thus, it is assumed that the coefficient for the variable *TWAD* in the population is different from zero.

***HWAD*:** The p-value for the coefficient of *HWAD* was .322. Thus, the p-value is greater than the significance level of 0.05 and the null hypothesis that the coefficient of *HWAD* is zero in the population is not rejected. Thus, it is assumed that the coefficient for the variable *HWAD* in the population is not different from zero.

***AWS*:** The p-value for the coefficient of *AWS* was <.001. Thus, the p-value is smaller than the significance level of 0.05 and the null hypothesis that the coefficient of *AWS* is zero in the population is rejected. Thus, it is assumed that the coefficient for the variable *AWS* in the population is different from zero.

***SOMC*:** The p-value for the coefficient of *SOMC* was .032. Thus, the p-value is smaller than the significance level of 0.05 and the null hypothesis that the coefficient of *SOMC* is zero in the population is rejected. Thus, it is assumed that the coefficient for the variable *SOMC* in the population is different from zero.

***GHG*:** The p-value for the coefficient of *GHG* was .005. Thus, the p-value is smaller than the significance level of 0.05 and the null hypothesis that the coefficient of *GHG* is zero in the population is rejected. Thus, it is assumed that the coefficient for the variable *GHG* in the population is different from zero.

***PARID*:** The p-value for the coefficient of *PARID* was .781. Thus, the p-value is greater than the significance level of 0.05 and the null hypothesis that the coefficient of *PARID* is zero in the population is not rejected. Thus, it is assumed that the coefficient for the variable *PARID* in the population is not different from zero.

***OCLEVEL*:** The p-value for the coefficient of *OCLEVEL* was .011. Thus, the p-value is smaller than the significance level of 0.05 and the null hypothesis that the coefficient of *OCLEVEL* is zero in the population is rejected. Thus, it is assumed that the coefficient for the variable *OCLEVEL* in the population is different from zero.

***SOILSCAPE 7*:** The p-value for the coefficient of *SOILSCAPE 7* was <.001. Thus, the p-value is smaller than the significance level of 0.05 and the null hypothesis that the coefficient of *SOILSCAPE 7* is zero in the population is rejected. Thus, it is assumed that the coefficient for the variable *SOILSCAPE 7* in the population is different from zero.

***SOILSCAPE 8*:** The p-value for the coefficient of *SOILSCAPE 8* was <.001. Thus, the p-value is smaller than the significance level of 0.05 and the null hypothesis that the coefficient of *SOILSCAPE 8* is zero in the population is rejected. Thus, it is assumed that the coefficient for the variable *SOILSCAPE 8* in the population is different from zero.

***TILLAGE 0*:** The p-value for the coefficient of *TILLAGE 0* was <.001. Thus, the p-value is smaller than the significance level of 0.05 and the null hypothesis that the coefficient of *TILLAGE 0* is zero in the population is rejected. Thus, it is assumed that the coefficient for the variable *TILLAGE 0* in the population is different from zero.

## Number of Cases

| n (Valid number of cases) | 1440 |
| --- | --- |

## Model Summary

| R | R^2^ | Adjusted R^2^ | Standard error of the estimate |
| --- | --- | --- | --- |
| 0.8 | 0.64 | 0.64 | 4.16 |

## ANOVA

| Model | df | F | p |
| --- | --- | --- | --- |
| Regression | 12 | 212.55 | <.001 |

## Coefficients

|  | Unstandardized Coefficients | Standardized Coefficients |  |  |  | 95% confidence interval for B | |
| --- | --- | --- | --- | --- | --- | --- | --- |
| Model | B | Beta | Standard error | t | p | lower bound | upper bound |
| (Constant) | -48.19 |  | 4.01 | -12.03 | <.001 | -56.05 | -40.33 |
| PRED | 0.03 | 0.44 | 0 | 23.28 | <.001 | 0.03 | 0.03 |
| TMEAN | 1.83 | 0.15 | 0.26 | 7.09 | <.001 | 1.32 | 2.34 |
| TWAD | 0 | -0.36 | 0 | -7.28 | <.001 | 0 | 0 |
| HWAD | 0 | -0.04 | 0 | -0.99 | .322 | 0 | 0 |
| AWS | 0.11 | 2 | 0.02 | 7.2 | <.001 | 0.08 | 0.14 |
| SOMC | 0 | -0.08 | 0 | -2.14 | .032 | 0 | 0 |
| GHG | 0 | 0.08 | 0 | 2.81 | .005 | 0 | 0 |
| PARID | 0.91 | 0.01 | 3.27 | 0.28 | .781 | -5.5 | 7.32 |
| OCLEVEL | 0.49 | 0.15 | 0.2 | 2.53 | .011 | 0.11 | 0.88 |
| SOILSCAPE 7 | 14.49 | 0.99 | 1.29 | 11.23 | <.001 | 11.96 | 17.02 |
| SOILSCAPE 8 | -16.1 | -1.1 | 3.23 | -4.99 | <.001 | -22.44 | -9.77 |
| TILLAGE 0 | -3.22 | -0.23 | 0.29 | -11.11 | <.001 | -3.78 | -2.65 |

## Residuals Statistics

|  | Min | Q1 | Median | Q3 | Max | Mean | SD |
| --- | --- | --- | --- | --- | --- | --- | --- |
| Residual | -17.84 | -2.53 | -0.1 | 2.25 | 21.32 | 0 | 4.15 |
| Std. Residual | -4.33 | -0.61 | -0.02 | 0.54 | 5.15 | 0 | 1 |

This table is a summary of the residuals from a regression analysis. Residuals are the differences between the observed values and the values predicted by the regression model. Let's interpret each component:

###### **Min**

The min value of -17.84 is the smallest residual in your data. It means the largest underestimation made by your model was by -17.84 units.

###### **Q1 (First Quartile)**

25% of the residuals are less than -2.53. These points are underestimations by the model.

###### **Median**

This is the median residual. Half of your residuals are lower than -0.1, and half are higher. A median close to zero suggests that, on the whole, the model is not systematically overestimating or underestimating.

###### **Q3 (Third Quartile)**

75% of the residuals are less than 2.25. These are overestimations by the model.

###### **Max**

The Max value of 21.32 is the largest residual. It indicates the largest overestimation by the model was by 21.32 units.

###### **Mean**

The average of the residuals is 0. If the mean is close to zero in linear regression models it indicates that the model is unbiased on average.

###### **SD (Standard Deviation)**

This value indicates the typical size of the residuals. A smaller standard deviation would indicate that the predictions are generally closer to the actual values.

###### **Standardized Residuals**

Standardized residuals are used to identify outliers in your data. Generally, a standardized residual greater than about 3 or less than about -3 might be considered an outlier. In your data, the range of standardized residuals suggests that there are some outliers (the min is -4.33 and the max is 5.15).


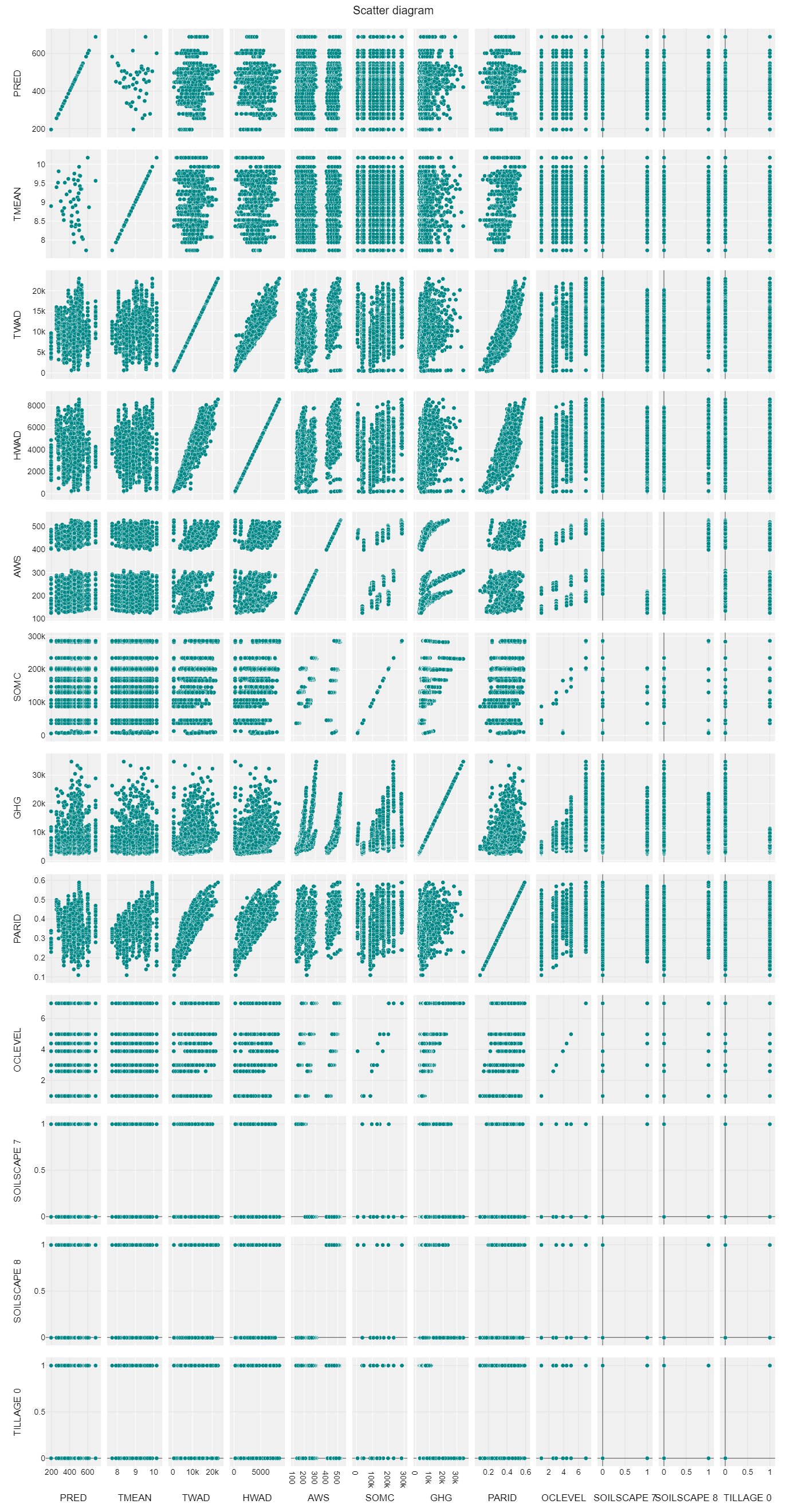


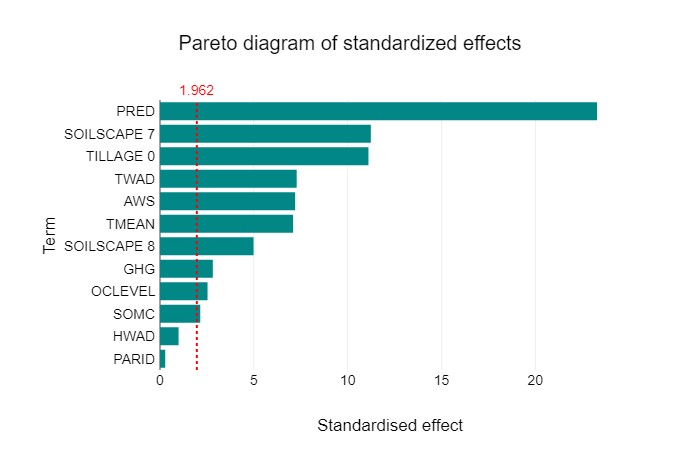


#### Linearity

To calculate a linear regression, there must be a linear relationship between the dependent and independent variables. In linear regression, a straight line is laid through the data; this only makes sense if there is linearity.


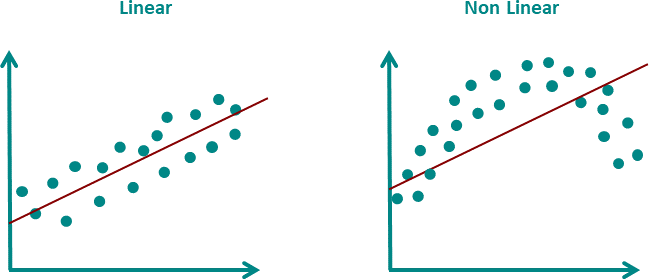


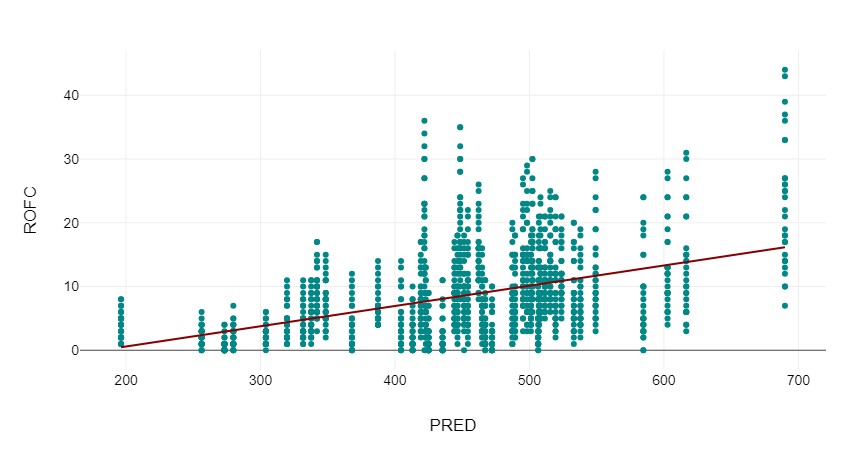
The following graphs result from this model:


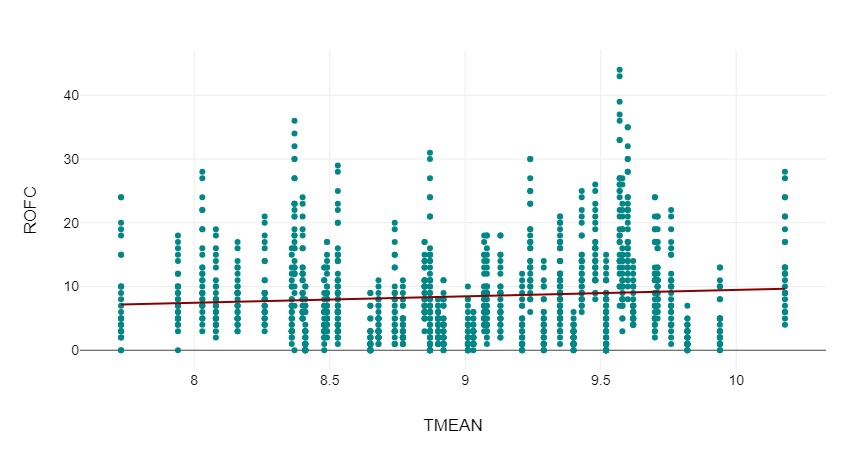


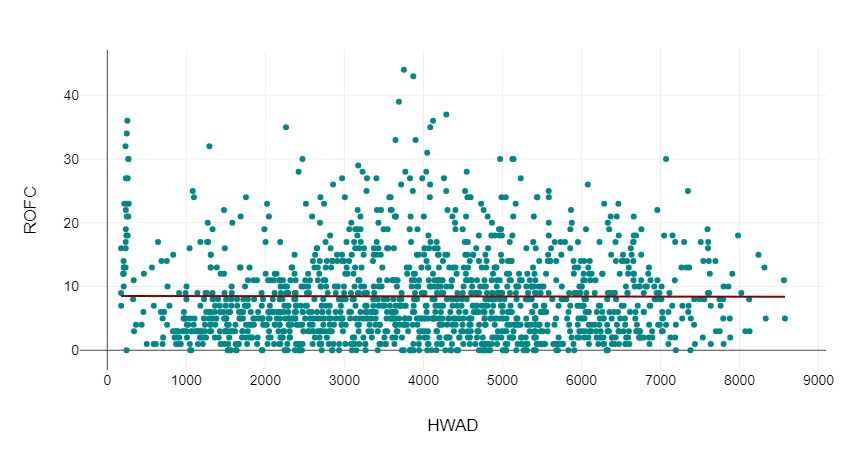


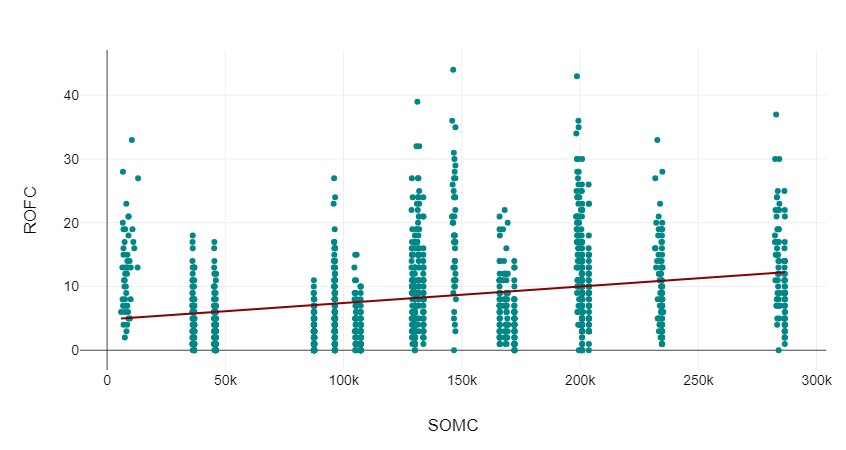


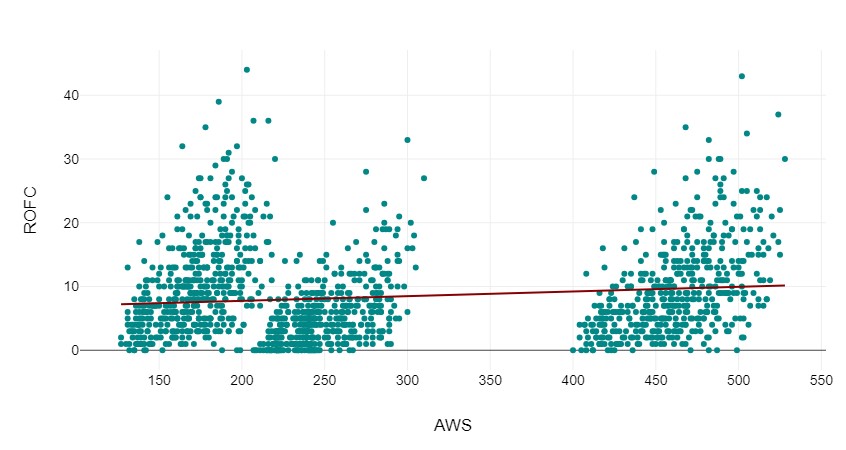


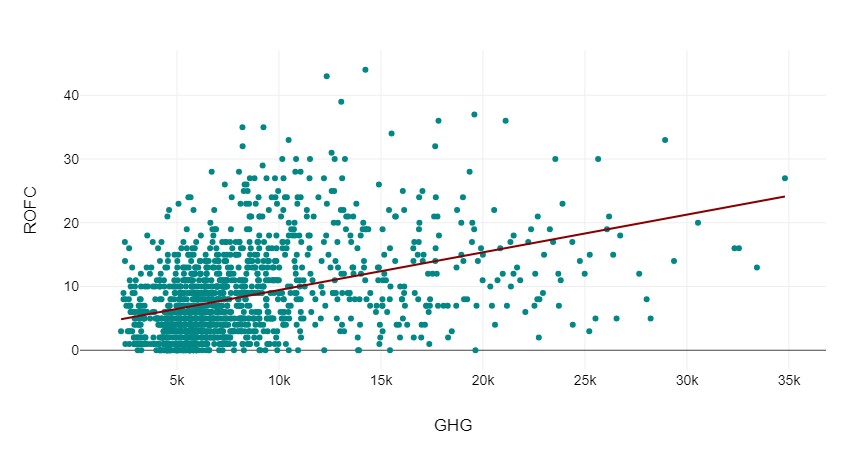


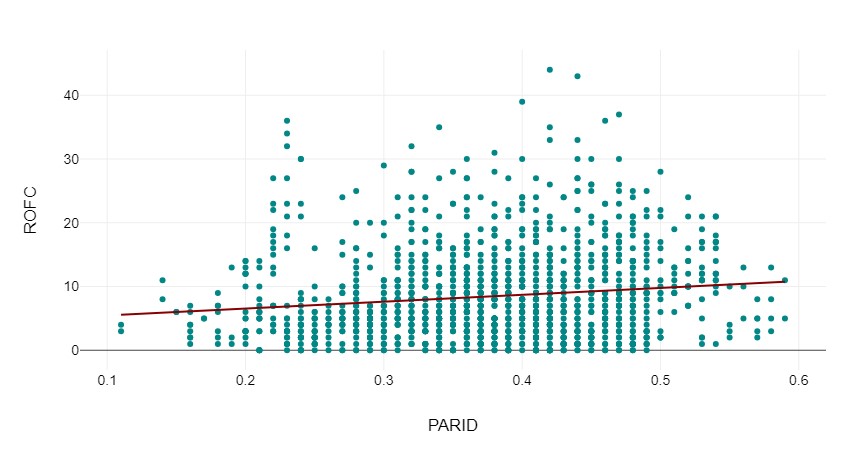


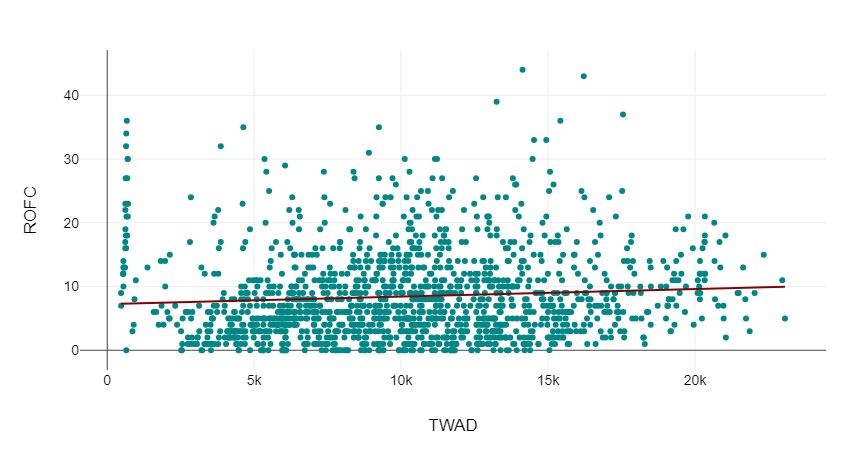


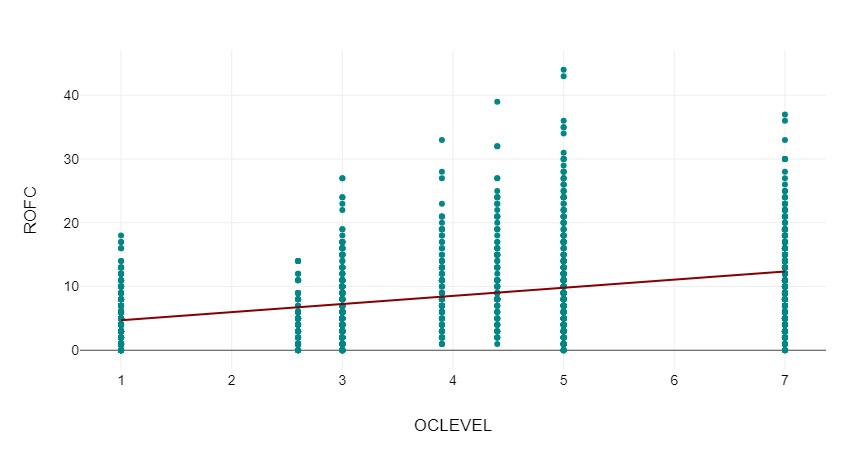


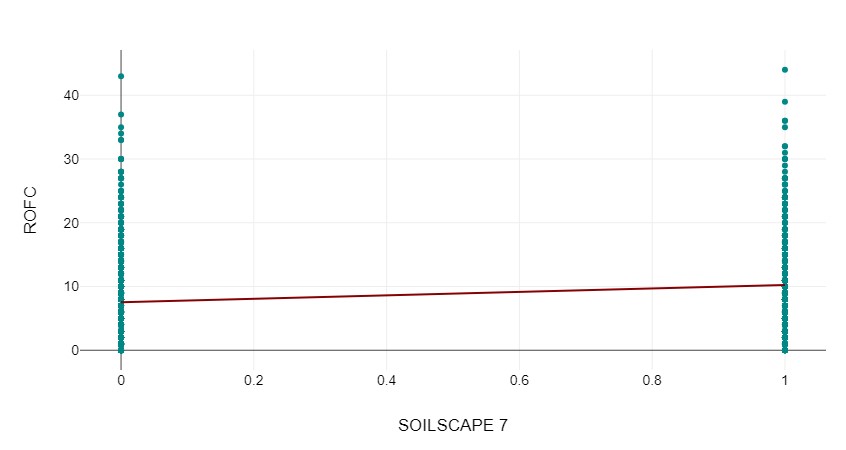


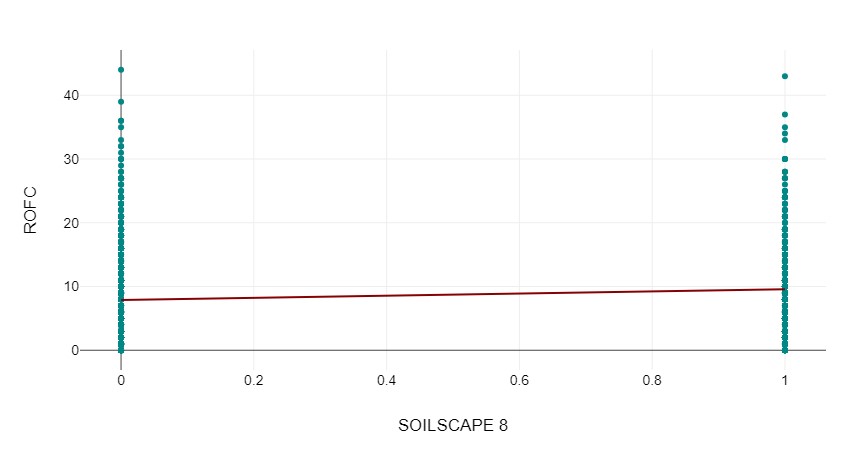


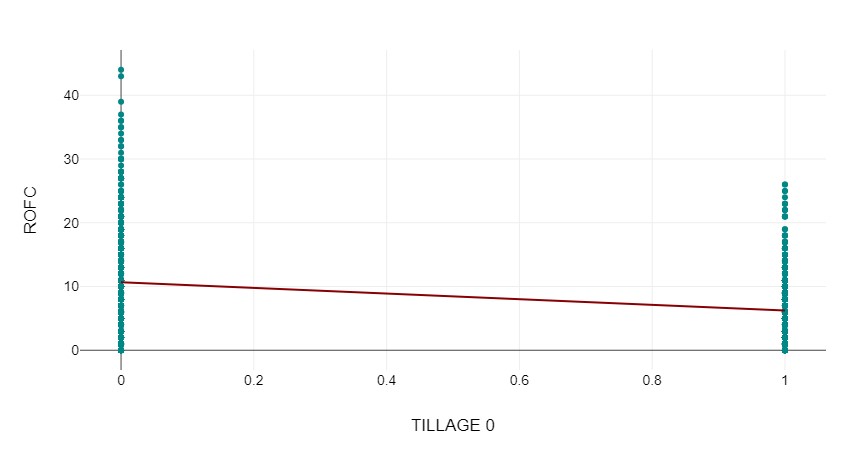


#### Normality of errors

## Tests for normal distribution of Residuum

|  | Statistics | p |
| --- | --- | --- |
| Kolmogorov-Smirnov | 0.05 | .002 |
| Kolmogorov-Smirnov (Lilliefors Corr.) | 0.05 | <.001 |
| Shapiro-Wilk | 0.97 | <.001 |
| Anderson-Darling | 5.8 | <.001 |


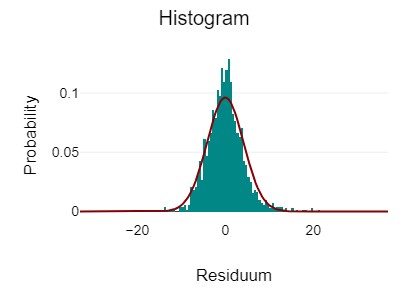


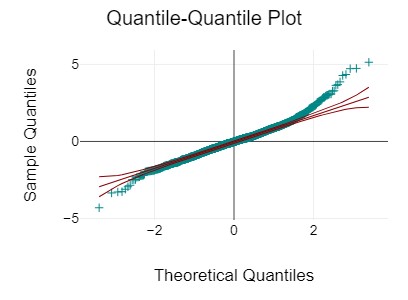


#### Autocorrelation of the Residual

## Durbin-Watson-Test

| Autocorrelation | Statistics | p |
| --- | --- | --- |
| 0.1 | 1.79 | <.001 |

The null hypothesis that the autocorrelation of the residual is zero is tested with a Durbin-Watson test. The calculated p-value of <.001 is smaller than 0.05, so that the null hypothesis is rejected and it can be assumed that there is autocorrelation of the residual.

#### Multicollinearity

Problematic if Tolerance < 0.10 or VIF > 10

| Model | Tolerance | VIF |
| --- | --- | --- |
| PRED | 0.7 | 1.42 |
| TMEAN | 0.54 | 1.86 |
| TWAD | 0.1 | 9.83 |
| HWAD | 0.13 | 7.42 |
| AWS | 0 | 305.55 |
| SOMC | 0.18 | 5.47 |
| GHG | 0.34 | 2.98 |
| PARID | 0.15 | 6.61 |
| OCLEVEL | 0.08 | 13.08 |
| SOILSCAPE 7 | 0.03 | 30.67 |
| SOILSCAPE 8 | 0.01 | 192.48 |
| TILLAGE 0 | 0.57 | 1.74 |

#### Heteroskedasticity

The variance of the residuals must be constant over the predicted values. Your data must therefore not exhibit heteroskedasticity.


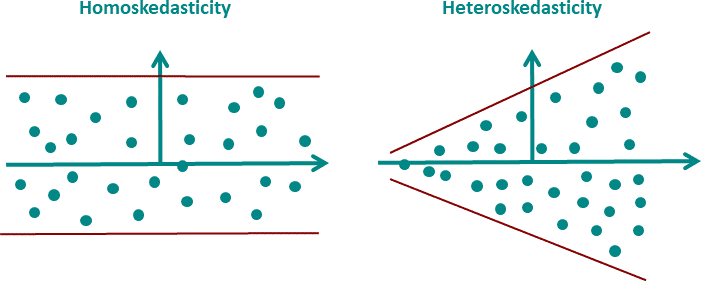


The model results in the following graph

 
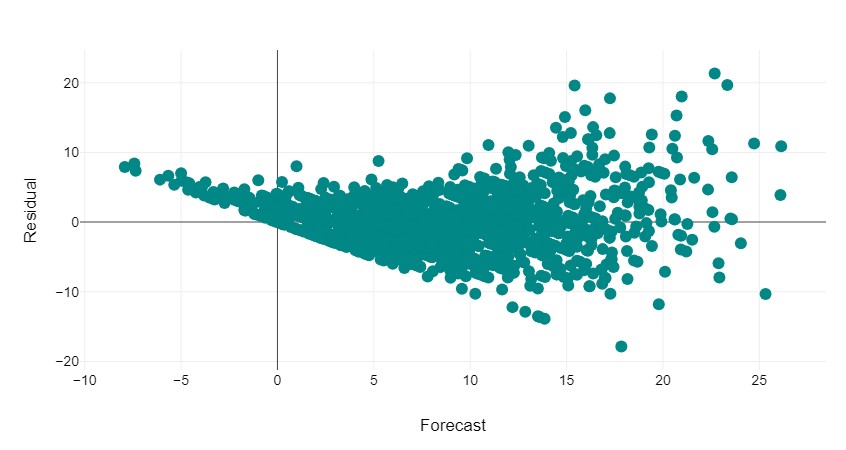


### Effect Size

#### Cohens f^2^

|  | f^2^ |
| --- | --- |
| PRED | 0.67 |
| TMEAN | 0.68 |
| TWAD | 0.68 |
| HWAD | 0.68 |
| AWS | 0.68 |
| SOMC | 0.68 |
| GHG | 0.68 |
| PARID | 0.68 |
| OCLEVEL | 0.68 |
| SOILSCAPE 7 | 0.68 |
| SOILSCAPE 8 | 0.68 |
| TILLAGE 0 | 0.68 |

1. **A multiple linear regression analysis was performed to examine the influence of the variables *PRED, TMEAN, TWAD, AWS, ROFC, SOMC, GHG, PARID, OCLEVEL, SOILSCAPE 7, SOILSCAPE 8 and TILLAGE 0* on the variable *HWAD*.**


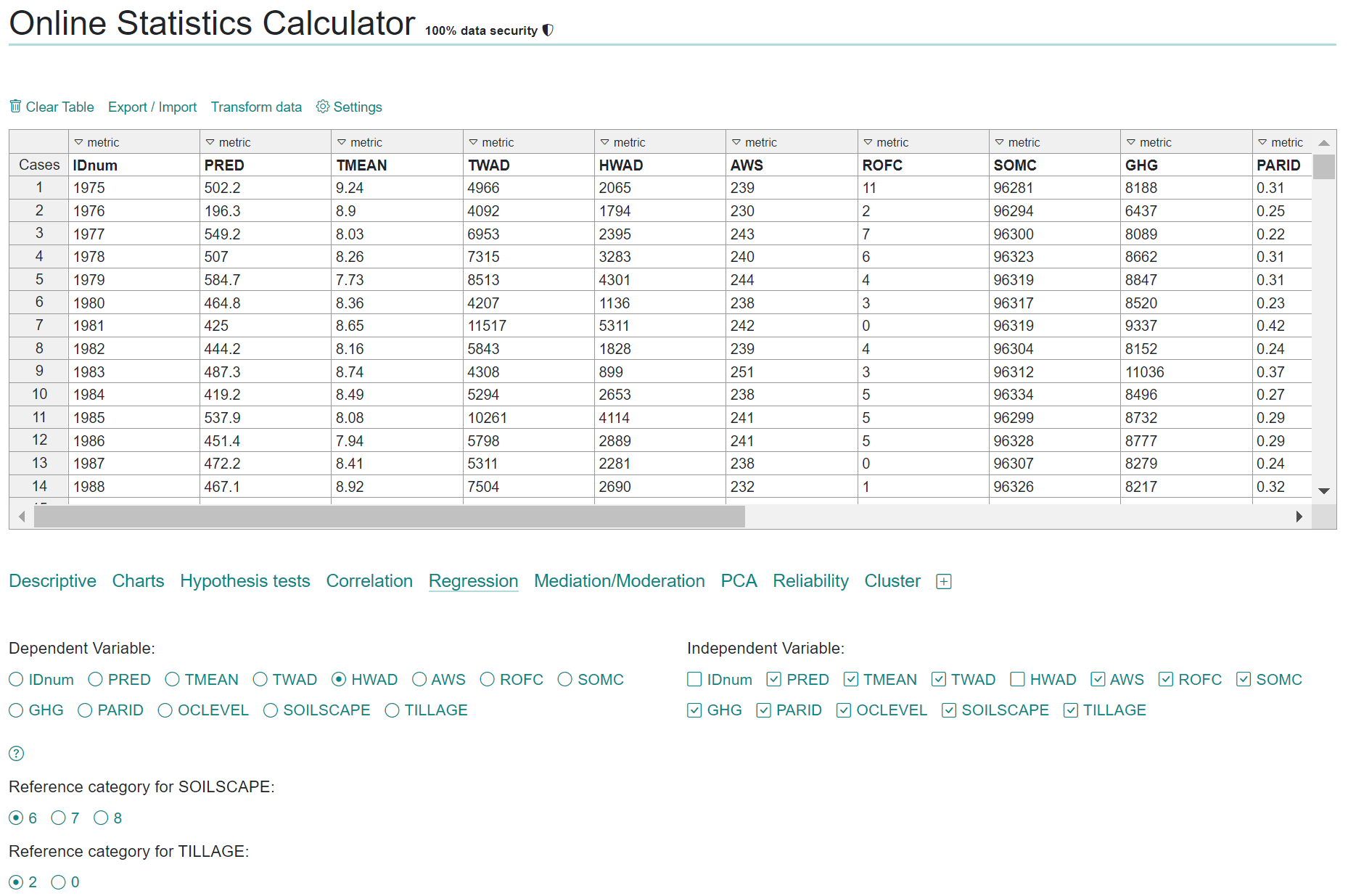


### Summary

#### Model Summary

The regression model showed that the variables *PRED, TMEAN, TWAD, AWS, ROFC, SOMC, GHG, PARID, OCLEVEL, SOILSCAPE 7, SOILSCAPE 8 and TILLAGE 0* explained 86.54% of the variance from the variable *HWAD*. An ANOVA was used to test whether this value was significantly different from zero. Using the present sample, it was found that the effect was significantly different from zero, *F*=764.64, *p* = <.001, *R^2^* = 0.87.

#### Regression coefficients

The following regression model is obtained:

HWAD = 10221.35 - 1.82 · PRED - 478.54 · TMEAN + 0.31 · TWAD - 28.02 · AWS - 4.36 · ROFC + 0 · SOMC - 0 · GHG + 3425.99 · PARID + 377.36 · OCLEVEL - 2386.43 · SOILSCAPE 7 + 6021.35 · SOILSCAPE 8 - 142.85 · TILLAGE 0

- ***Constant*:**When all independent variables are equal to zero, the value of the variable *HWAD* is 10221.35.
- ***PRED*:**If the value of the variable *PRED* changes by one unit, the value of the variable *HWAD* changes by -1.82.
- ***TMEAN*:**If the value of the variable *TMEAN* changes by one unit, the value of the variable *HWAD* changes by -478.54.
- ***TWAD*:**If the value of the variable *TWAD* changes by one unit, the value of the variable *HWAD* changes by 0.31.
- ***AWS*:**If the value of the variable *AWS* changes by one unit, the value of the variable *HWAD* changes by -28.02.
- ***ROFC*:**If the value of the variable *ROFC* changes by one unit, the value of the variable *HWAD* changes by -4.36.
- ***SOMC*:**If the value of the variable *SOMC* changes by one unit, the value of the variable *HWAD* changes by 0.
- ***GHG*:**If the value of the variable *GHG* changes by one unit, the value of the variable *HWAD* changes by 0.
- ***PARID*:**If the value of the variable *PARID* changes by one unit, the value of the variable *HWAD* changes by 3425.99.
- ***OCLEVEL*:**If the value of the variable *OCLEVEL* changes by one unit, the value of the variable *HWAD* changes by 377.36.
- ***SOILSCAPE 7*:**If the value of the variable *SOILSCAPE 7* changes by one unit, the value of the variable *HWAD* changes by -2386.43.
- ***SOILSCAPE 8*:**If the value of the variable *SOILSCAPE 8* changes by one unit, the value of the variable *HWAD* changes by 6021.35.
- ***TILLAGE 0*:**If the value of the variable *TILLAGE 0* changes by one unit, the value of the variable *HWAD* changes by -142.85.

#### Standardized regression coefficients

The standardized coefficients beta are independent of the measured variable and are always between -1 and 1. The larger the amount of beta, the greater the contribution of the respective independent variable to explain the dependent variable *HWAD*. In this model, the variable *"AWS"* has the greatest influence on the variable *HWAD*.

#### p-value

The calculated regression coefficients refer to the sample used for the calculation of the regression analysis, therefore it is of interest whether the individual coefficients only deviate from zero by chance or whether they also deviate from zero in the population. To test this, the null hypothesis was made for each coefficient that was equal to zero in the population.

The standard error now indicates how much the respective coefficient will scatter on average when the regression analysis is calculated for a further sample. The test statistic t is then calculated from the standard error and the coefficient.

***PRED*:** The p-value for the coefficient of *PRED* was <.001. Thus, the p-value is smaller than the significance level of 0.05 and the null hypothesis that the coefficient of *PRED* is zero in the population is rejected. Thus, it is assumed that the coefficient for the variable *PRED* in the population is different from zero.

***TMEAN*:** The p-value for the coefficient of *TMEAN* was <.001. Thus, the p-value is smaller than the significance level of 0.05 and the null hypothesis that the coefficient of *TMEAN* is zero in the population is rejected. Thus, it is assumed that the coefficient for the variable *TMEAN* in the population is different from zero.

***TWAD*:** The p-value for the coefficient of *TWAD* was <.001. Thus, the p-value is smaller than the significance level of 0.05 and the null hypothesis that the coefficient of *TWAD* is zero in the population is rejected. Thus, it is assumed that the coefficient for the variable *TWAD* in the population is different from zero.

***AWS*:** The p-value for the coefficient of *AWS* was <.001. Thus, the p-value is smaller than the significance level of 0.05 and the null hypothesis that the coefficient of *AWS* is zero in the population is rejected. Thus, it is assumed that the coefficient for the variable *AWS* in the population is different from zero.

***ROFC*:** The p-value for the coefficient of *ROFC* was .322. Thus, the p-value is greater than the significance level of 0.05 and the null hypothesis that the coefficient of *ROFC* is zero in the population is not rejected. Thus, it is assumed that the coefficient for the variable *ROFC* in the population is not different from zero.

***SOMC*:** The p-value for the coefficient of *SOMC* was .27. Thus, the p-value is greater than the significance level of 0.05 and the null hypothesis that the coefficient of *SOMC* is zero in the population is not rejected. Thus, it is assumed that the coefficient for the variable *SOMC* in the population is not different from zero.

***GHG*:** The p-value for the coefficient of *GHG* was .488. Thus, the p-value is greater than the significance level of 0.05 and the null hypothesis that the coefficient of *GHG* is zero in the population is not rejected. Thus, it is assumed that the coefficient for the variable *GHG* in the population is not different from zero.

***PARID*:** The p-value for the coefficient of *PARID* was <.001. Thus, the p-value is smaller than the significance level of 0.05 and the null hypothesis that the coefficient of *PARID* is zero in the population is rejected. Thus, it is assumed that the coefficient for the variable *PARID* in the population is different from zero.

***OCLEVEL*:** The p-value for the coefficient of *OCLEVEL* was <.001. Thus, the p-value is smaller than the significance level of 0.05 and the null hypothesis that the coefficient of *OCLEVEL* is zero in the population is rejected. Thus, it is assumed that the coefficient for the variable *OCLEVEL* in the population is different from zero.

***SOILSCAPE 7*:** The p-value for the coefficient of *SOILSCAPE 7* was <.001. Thus, the p-value is smaller than the significance level of 0.05 and the null hypothesis that the coefficient of *SOILSCAPE 7* is zero in the population is rejected. Thus, it is assumed that the coefficient for the variable *SOILSCAPE 7* in the population is different from zero.

***SOILSCAPE 8*:** The p-value for the coefficient of *SOILSCAPE 8* was <.001. Thus, the p-value is smaller than the significance level of 0.05 and the null hypothesis that the coefficient of *SOILSCAPE 8* is zero in the population is rejected. Thus, it is assumed that the coefficient for the variable *SOILSCAPE 8* in the population is different from zero.

***TILLAGE 0*:** The p-value for the coefficient of *TILLAGE 0* was .004. Thus, the p-value is smaller than the significance level of 0.05 and the null hypothesis that the coefficient of *TILLAGE 0* is zero in the population is rejected. Thus, it is assumed that the coefficient for the variable *TILLAGE 0* in the population is different from zero.

## Number of Cases

| n (Valid number of cases) | 1440 |
| --- | --- |

## Model Summary

| R | R^2^ | Adjusted R^2^ | Standard error of the estimate |
| --- | --- | --- | --- |
| 0.93 | 0.87 | 0.86 | 692.56 |

A multiple linear regression analysis was performed to examine the influence of the variables *PRED, TMEAN, TWAD, AWS, ROFC, SOMC, GHG, PARID, OCLEVEL, SOILSCAPE 7, SOILSCAPE 8 and TILLAGE 0* on the variable *HWAD*. Here's how to interpret the Model Summary.

###### **R (Correlation Coefficient)**

R is the correlation between the observed values of the dependent variable *HWAD* and the predictions made by the model using the independent variables. This R value of 0.93 indicates a very high positive correlation between the observed values and the prediction made by this model.

###### **R² (R-squared)**

R² is the proportion of the variance in the dependent variable that can be explained by the independent variables in the regression model. This R² value of 0.87 means that 86.54% of the variance in your dependent variable is explained by the independent variables in your model. In other words, 86.54% of the change in *HWAD* can be predicted from the independent variables.

###### **Adjusted R²**

Adjusted R-squared adjusts the R² value based on the number of variables in the model and the number of observations. It's a more accurate measure when you have multiple independent variables. Here, it suggests that after adjusting for the number of predictors, about 86.43% of the variance in the dependent variable is accounted for.

###### **Standard Error of the Estimate**

This value indicates the average distance that the observed values fall from the regression line. Essentially, it's a measure of the accuracy of predictions made with your regression model. A standard error of 692.56 means that the predicted values are, on average, 692.56 units away from the actual values. Whether this is a small or large error depends on the context and scale of your dependent variable.

###### **Summary**

In summary, your model shows a very high positive relationship between the observed values and the prediction, explains 86.54% of the variance in the dependent variable, but the predictions are on average 692.56 units away from the actual values, which may or may not be significant depending on the context of your data.

## ANOVA

| Model | df | F | p |
| --- | --- | --- | --- |
| Regression | 12 | 764.64 | <.001 |

## Coefficients

|  | Unstandardized Coefficients | Standardized Coefficients |  |  |  | 95% confidence interval for B | |
| --- | --- | --- | --- | --- | --- | --- | --- |
| Model | B | Beta | Standard error | t | p | lower bound | upper bound |
| (Constant) | 10221.35 |  | 644.69 | 15.85 | <.001 | 8956.46 | 11486.23 |
| PRED | -1.82 | -0.09 | 0.26 | -7.1 | <.001 | -2.33 | -1.32 |
| TMEAN | -478.54 | -0.15 | 41.79 | -11.45 | <.001 | -560.54 | -396.55 |
| TWAD | 0.31 | 0.77 | 0.01 | 32.74 | <.001 | 0.3 | 0.33 |
| AWS | -28.02 | -1.87 | 2.49 | -11.27 | <.001 | -32.9 | -23.15 |
| ROFC | -4.36 | -0.02 | 4.4 | -0.99 | .322 | -12.99 | 4.28 |
| SOMC | 0 | 0.03 | 0 | 1.1 | .27 | 0 | 0 |
| GHG | 0 | -0.01 | 0.01 | -0.69 | .488 | -0.02 | 0.01 |
| PARID | 3425.99 | 0.16 | 535.52 | 6 |  |  |  |

## Residuals Statistics

|  | Min | Q1 | Median | Q3 | Max | Mean | SD |
| --- | --- | --- | --- | --- | --- | --- | --- |
| Residual | -2444.49 | -536.69 | 75.52 | 489.8 | 1844.39 | 0 | 689.66 |
| Std. Residual | -3.54 | -0.78 | 0.11 | 0.71 | 2.67 | 0 | 1 |

### Assumptions

#### Linearity

To calculate a linear regression, there must be a linear relationship between the dependent and independent variables. In linear regression, a straight line is laid through the data; this only makes sense if there is linearity.

The following graphs result from this model:


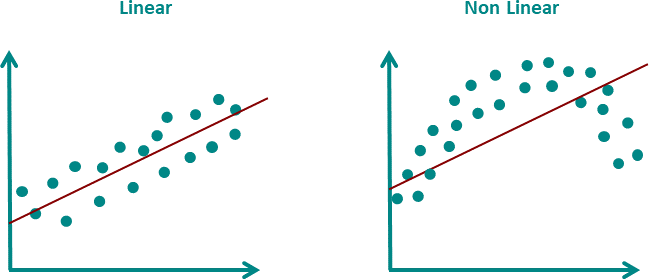


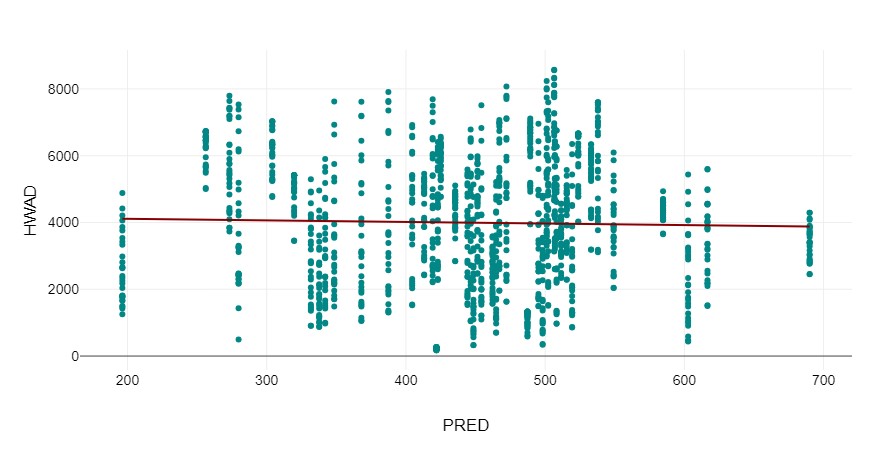


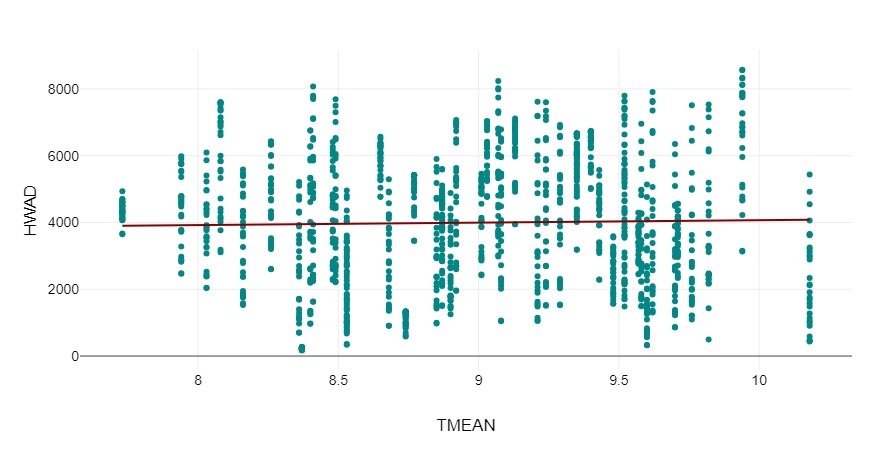


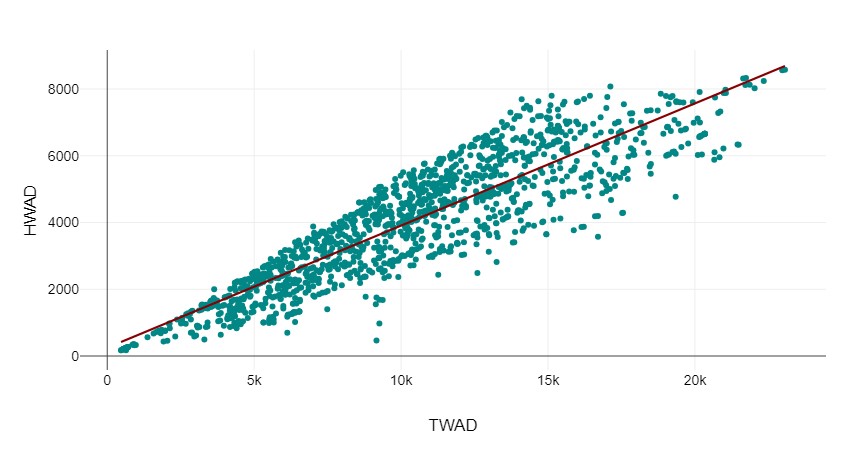


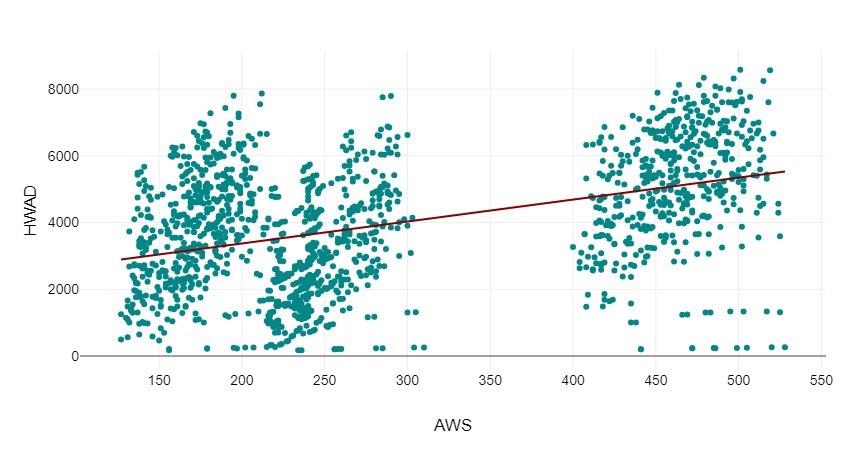


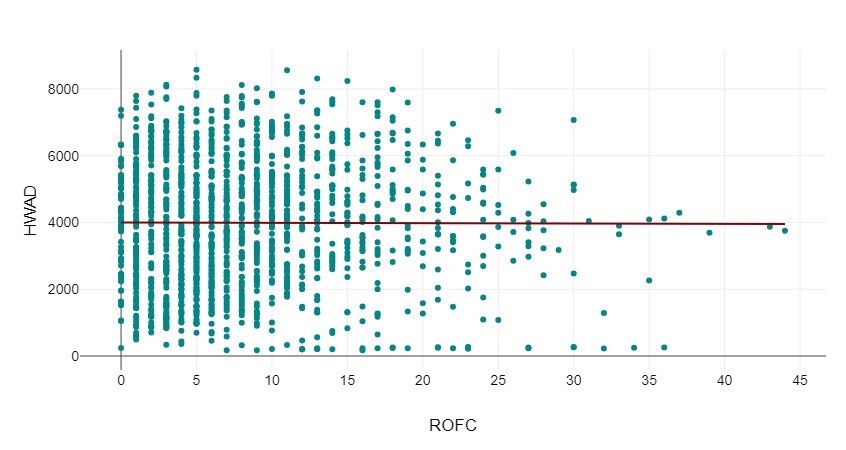


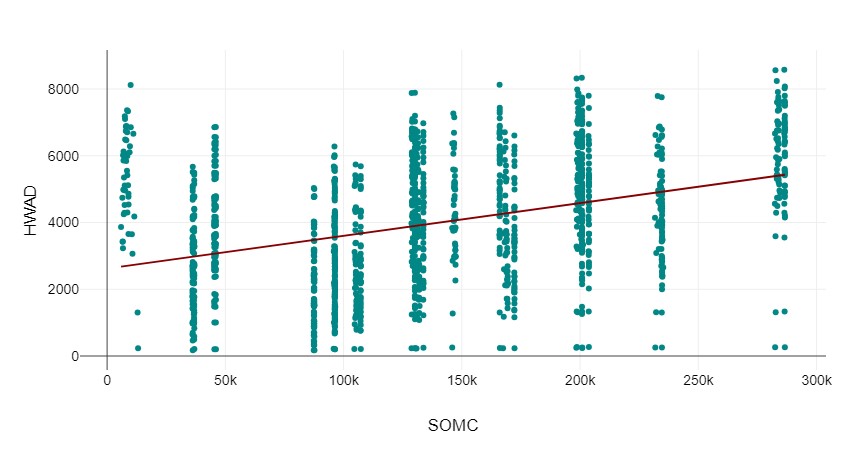


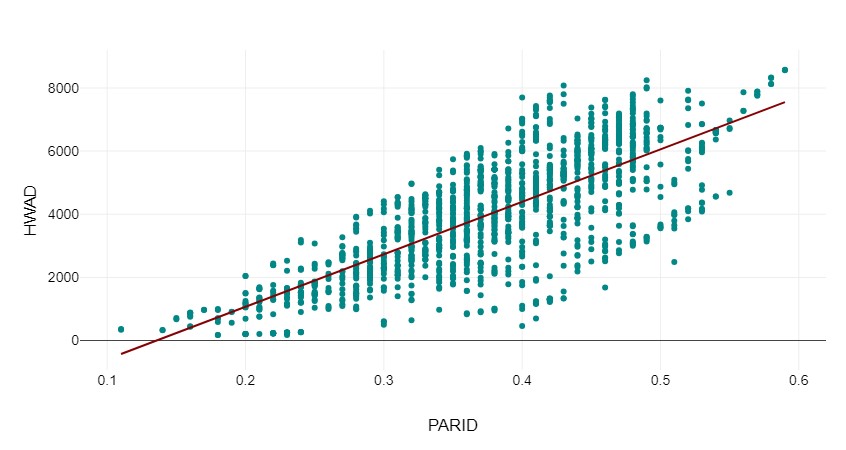


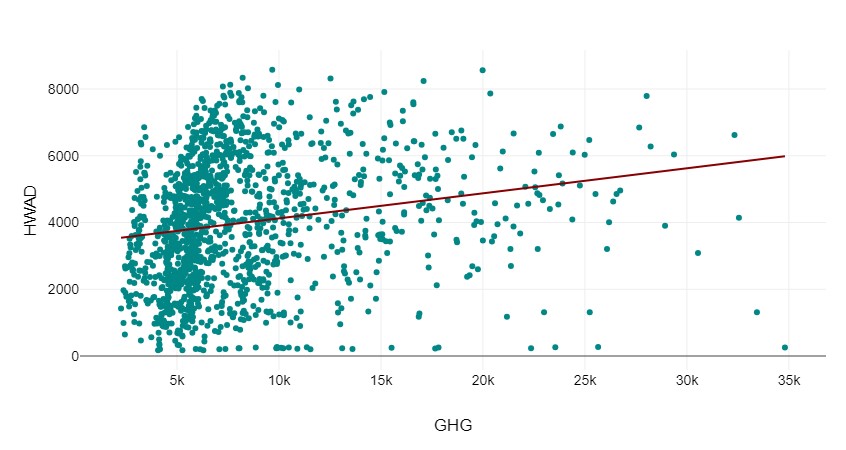


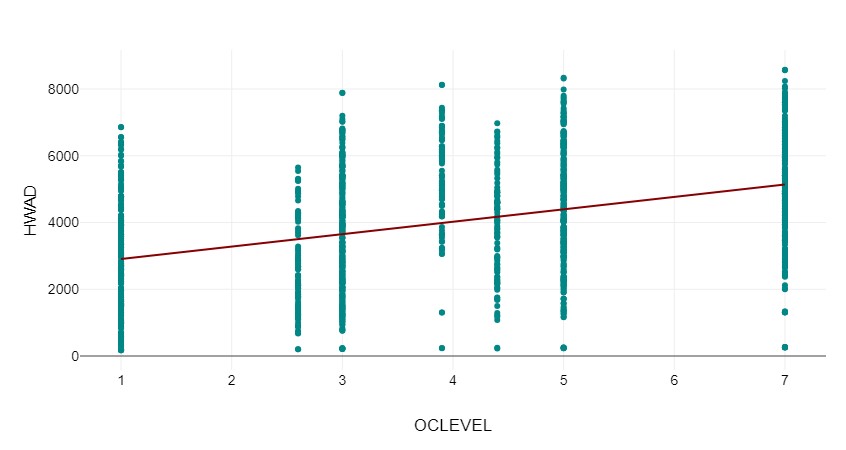


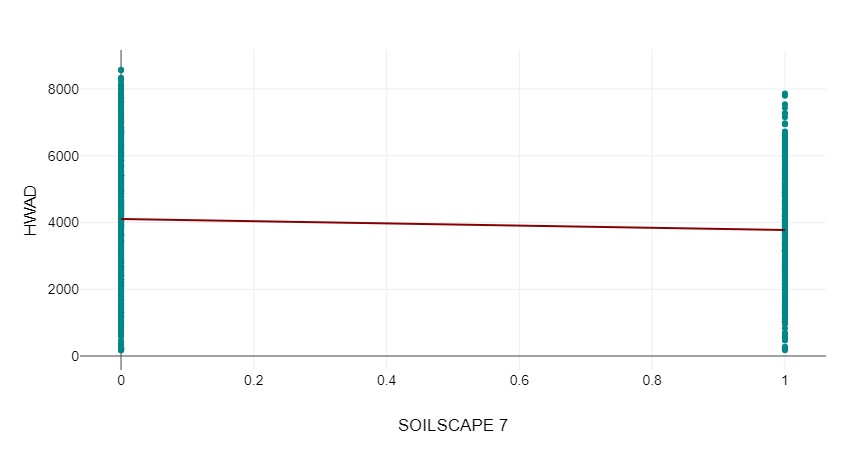


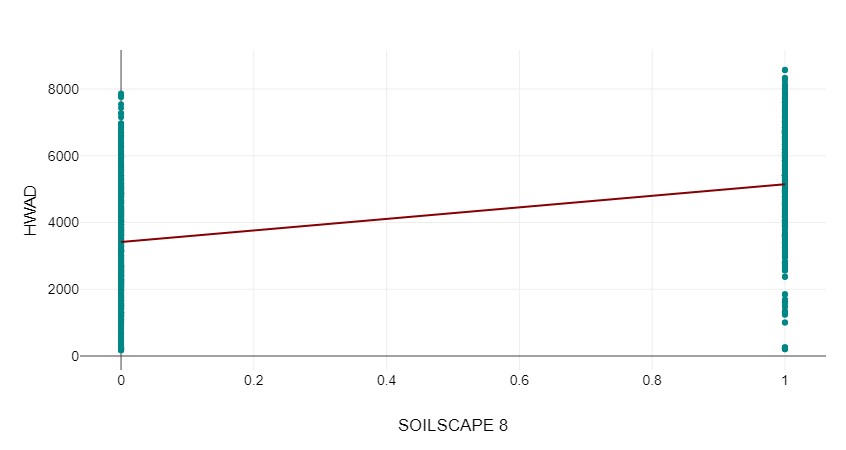


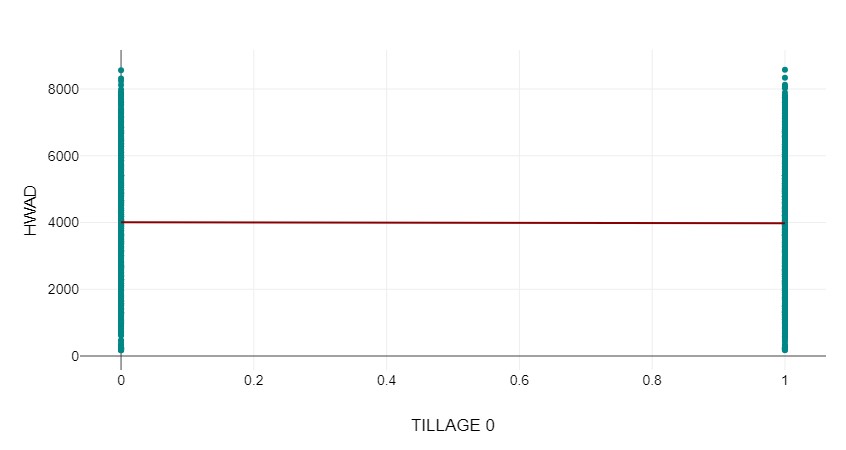


#### Normality of errors

## Tests for normal distribution of Residuum

|  | Statistics | p |
| --- | --- | --- |
| Kolmogorov-Smirnov | 0.05 | .002 |
| Kolmogorov-Smirnov (Lilliefors Corr.) | 0.05 | <.001 |
| Shapiro-Wilk | 0.99 | <.001 |
| Anderson-Darling | 3.83 | <.001 |


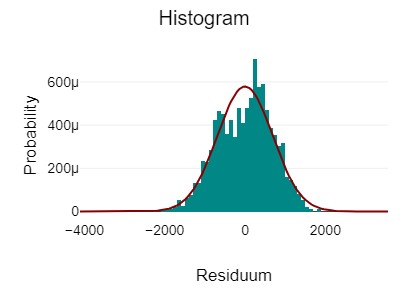


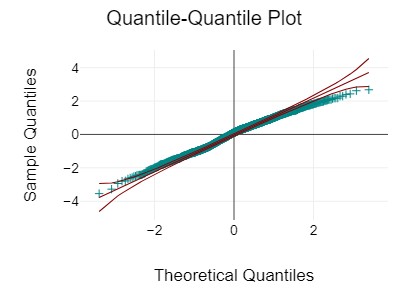


#### Autocorrelation of the Residual

## Durbin-Watson-Test

| Autocorrelation | Statistics | p |
| --- | --- | --- |
| 0.04 | 1.92 | .101 |

The null hypothesis that the autocorrelation of the residual is zero is tested with a Durbin-Watson test. The calculated p-value of .101 is greater than 0.05, so that the null hypothesis is not rejected and it can be assumed that there is no autocorrelation of the residual.

#### Multicollinearity

Problematic if Tolerance < 0.10 or VIF > 10

| Model | Tolerance | VIF |
| --- | --- | --- |
| PRED | 0.53 | 1.89 |
| TMEAN | 0.57 | 1.77 |
| TWAD | 0.17 | 5.82 |
| AWS | 0 | 290.76 |
| ROFC | 0.36 | 2.79 |
| SOMC | 0.18 | 5.48 |
| GHG | 0.33 | 3 |
| PARID | 0.16 | 6.43 |
| OCLEVEL | 0.08 | 11.9 |
| SOILSCAPE 7 | 0.03 | 30.72 |
| SOILSCAPE 8 | 0.01 | 178.88 |
| TILLAGE 0 | 0.53 | 1.88 |

#### Heteroskedasticity


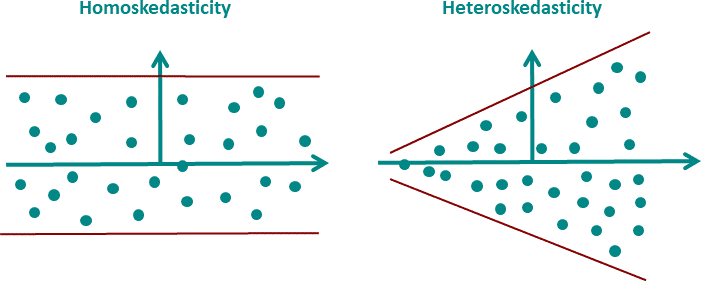
The variance of the residuals must be constant over the predicted values. Your data must therefore not exhibit heteroskedasticity.

This model results in the following graph


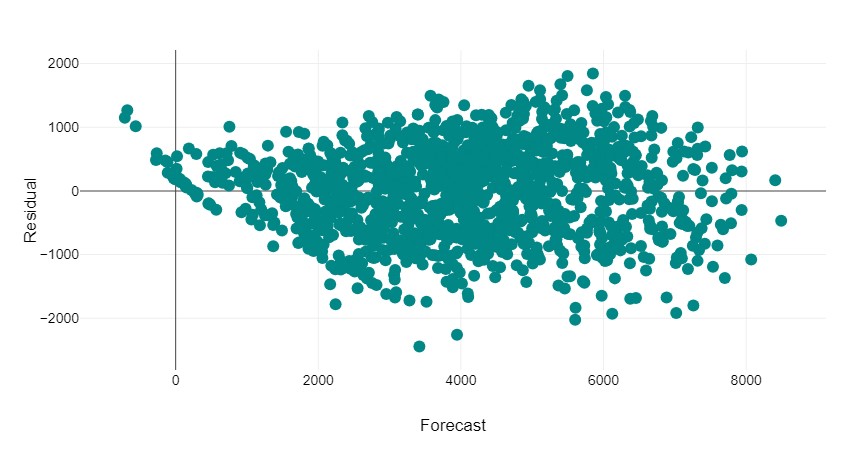


### Effect Size

#### Cohens f^2^

|  | f^2^ |
| --- | --- |
| PRED | 3.53 |
| TMEAN | 3.53 |
| TWAD | 3.53 |
| AWS | 3.53 |
| ROFC | 3.53 |
| SOMC | 3.53 |
| GHG | 3.53 |
| PARID | 3.54 |
| OCLEVEL | 3.54 |
| SOILSCAPE 7 | 3.53 |
| SOILSCAPE 8 | 3.53 |
| TILLAGE 0 | 3.54 |

1. **A multiple linear regression analysis was performed to examine the influence of the variables *PRED, TMEAN, TWAD, HWAD, AWS, ROFC, SOMC, PARID, OCLEVEL, SOILSCAPE 7, SOILSCAPE 8 and TILLAGE 0* on the variable *GHG*.**


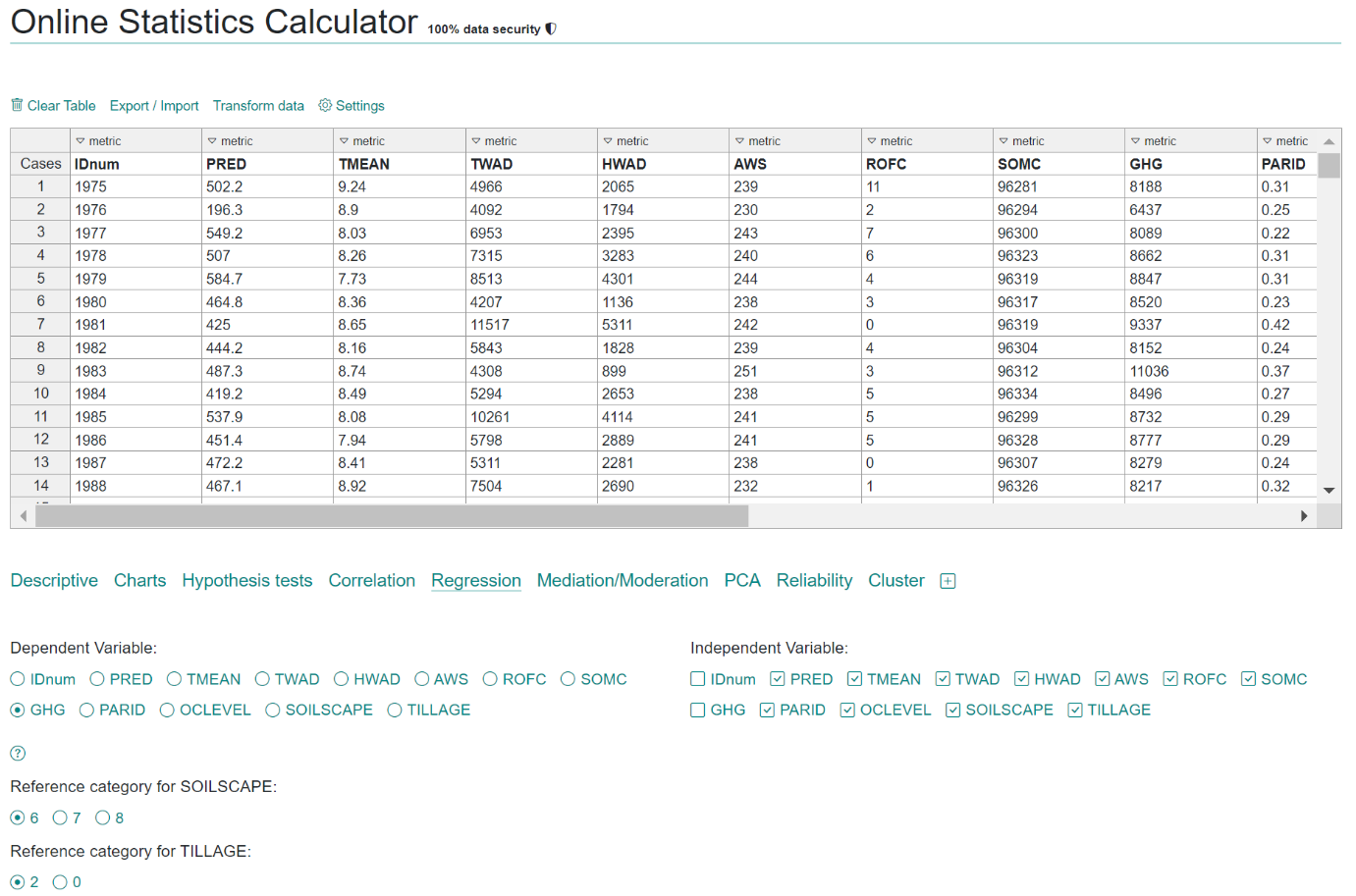


### Summary

#### Model Summary

The regression model showed that the variables *PRED, TMEAN, TWAD, HWAD, AWS, ROFC, SOMC, PARID, OCLEVEL, SOILSCAPE 7, SOILSCAPE 8 and TILLAGE 0* explained 66.67% of the variance from the variable *GHG*. An ANOVA was used to test whether this value was significantly different from zero. Using the present sample, it was found that the effect was significantly different from zero, *F*=237.86, *p* = <.001, *R^2^* = 0.67.

#### Regression coefficients

The following regression model is obtained:

GHG = -16543.29 + 0.16 · PRED - 159.03 · TMEAN - 0.03 · TWAD - 0.07 · HWAD + 102.8 · AWS + 50.2 · ROFC + 0 · SOMC + 8225.46 · PARID + 98.49 · OCLEVEL + 5911.34 · SOILSCAPE 7 - 23917.47 · SOILSCAPE 8 - 3446.46 · TILLAGE 0

- ***Constant*:**When all independent variables are equal to zero, the value of the variable *GHG* is -16543.29.
- ***PRED*:**If the value of the variable *PRED* changes by one unit, the value of the variable *GHG* changes by 0.16.
- ***TMEAN*:**If the value of the variable *TMEAN* changes by one unit, the value of the variable *GHG* changes by -159.03.
- ***TWAD*:**If the value of the variable *TWAD* changes by one unit, the value of the variable *GHG* changes by -0.03.
- ***HWAD*:**If the value of the variable *HWAD* changes by one unit, the value of the variable *GHG* changes by -0.07.
- ***AWS*:**If the value of the variable *AWS* changes by one unit, the value of the variable *GHG* changes by 102.8.
- ***ROFC*:**If the value of the variable *ROFC* changes by one unit, the value of the variable *GHG* changes by 50.2.
- ***SOMC*:**If the value of the variable *SOMC* changes by one unit, the value of the variable *GHG* changes by 0.
- ***PARID*:**If the value of the variable *PARID* changes by one unit, the value of the variable *GHG* changes by 8225.46.
- ***OCLEVEL*:**If the value of the variable *OCLEVEL* changes by one unit, the value of the variable *GHG* changes by 98.49.
- ***SOILSCAPE 7*:**If the value of the variable *SOILSCAPE 7* changes by one unit, the value of the variable *GHG* changes by 5911.34.
- ***SOILSCAPE 8*:**If the value of the variable *SOILSCAPE 8* changes by one unit, the value of the variable *GHG* changes by -23917.47.
- ***TILLAGE 0*:**If the value of the variable *TILLAGE 0* changes by one unit, the value of the variable *GHG* changes by -3446.46.

#### Standardized regression coefficients

The standardized coefficients beta are independent of the measured variable and are always between -1 and 1. The larger the amount of beta, the greater the contribution of the respective independent variable to explain the dependent variable *GHG*. In this model, the variable *"AWS"* has the greatest influence on the variable *GHG*.

#### p-value

The calculated regression coefficients refer to the sample used for the calculation of the regression analysis, therefore it is of interest whether the individual coefficients only deviate from zero by chance or whether they also deviate from zero in the population. To test this, the null hypothesis was made for each coefficient that was equal to zero in the population.

The standard error now indicates how much the respective coefficient will scatter on average when the regression analysis is calculated for a further sample. The test statistic t is then calculated from the standard error and the coefficient.

***PRED*:** The p-value for the coefficient of *PRED* was .881. Thus, the p-value is greater than the significance level of 0.05 and the null hypothesis that the coefficient of *PRED* is zero in the population is not rejected. Thus, it is assumed that the coefficient for the variable *PRED* in the population is not different from zero.

***TMEAN*:** The p-value for the coefficient of *TMEAN* was .37. Thus, the p-value is greater than the significance level of 0.05 and the null hypothesis that the coefficient of *TMEAN* is zero in the population is not rejected. Thus, it is assumed that the coefficient for the variable *TMEAN* in the population is not different from zero.

***TWAD*:** The p-value for the coefficient of *TWAD* was .525. Thus, the p-value is greater than the significance level of 0.05 and the null hypothesis that the coefficient of *TWAD* is zero in the population is not rejected. Thus, it is assumed that the coefficient for the variable *TWAD* in the population is not different from zero.

***HWAD*:** The p-value for the coefficient of *HWAD* was .488. Thus, the p-value is greater than the significance level of 0.05 and the null hypothesis that the coefficient of *HWAD* is zero in the population is not rejected. Thus, it is assumed that the coefficient for the variable *HWAD* in the population is not different from zero.

***AWS*:** The p-value for the coefficient of *AWS* was <.001. Thus, the p-value is smaller than the significance level of 0.05 and the null hypothesis that the coefficient of *AWS* is zero in the population is rejected. Thus, it is assumed that the coefficient for the variable *AWS* in the population is different from zero.

***ROFC*:** The p-value for the coefficient of *ROFC* was .005. Thus, the p-value is smaller than the significance level of 0.05 and the null hypothesis that the coefficient of *ROFC* is zero in the population is rejected. Thus, it is assumed that the coefficient for the variable *ROFC* in the population is different from zero.

***SOMC*:** The p-value for the coefficient of *SOMC* was .265. Thus, the p-value is greater than the significance level of 0.05 and the null hypothesis that the coefficient of *SOMC* is zero in the population is not rejected. Thus, it is assumed that the coefficient for the variable *SOMC* in the population is not different from zero.

***PARID*:** The p-value for the coefficient of *PARID* was <.001. Thus, the p-value is smaller than the significance level of 0.05 and the null hypothesis that the coefficient of *PARID* is zero in the population is rejected. Thus, it is assumed that the coefficient for the variable *PARID* in the population is different from zero.

***OCLEVEL*:** The p-value for the coefficient of *OCLEVEL* was .456. Thus, the p-value is greater than the significance level of 0.05 and the null hypothesis that the coefficient of *OCLEVEL* is zero in the population is not rejected. Thus, it is assumed that the coefficient for the variable *OCLEVEL* in the population is not different from zero.

***SOILSCAPE 7*:** The p-value for the coefficient of *SOILSCAPE 7* was <.001. Thus, the p-value is smaller than the significance level of 0.05 and the null hypothesis that the coefficient of *SOILSCAPE 7* is zero in the population is rejected. Thus, it is assumed that the coefficient for the variable *SOILSCAPE 7* in the population is different from zero.

***SOILSCAPE 8*:** The p-value for the coefficient of *SOILSCAPE 8* was <.001. Thus, the p-value is smaller than the significance level of 0.05 and the null hypothesis that the coefficient of *SOILSCAPE 8* is zero in the population is rejected. Thus, it is assumed that the coefficient for the variable *SOILSCAPE 8* in the population is different from zero.

***TILLAGE 0*:** The p-value for the coefficient of *TILLAGE 0* was <.001. Thus, the p-value is smaller than the significance level of 0.05 and the null hypothesis that the coefficient of *TILLAGE 0* is zero in the population is rejected. Thus, it is assumed that the coefficient for the variable *TILLAGE 0* in the population is different from zero.

## Number of Cases

| n (Valid number of cases) | 1440 |
| --- | --- |

## Model Summary

| R | R^2^ | Adjusted R^2^ | Standard error of the estimate |
| --- | --- | --- | --- |
| 0.82 | 0.67 | 0.66 | 2814.18 |

## ANOVA

| Model | df | F | p |
| --- | --- | --- | --- |
| Regression | 12 | 237.86 | <.001 |

|  | Unstandardized Coefficients | Standardized Coefficients |  |  |  | 95% confidence interval for B | |
| --- | --- | --- | --- | --- | --- | --- | --- |
| Model | B | Beta | Standard error | t | p | lower bound | upper bound |
| (Constant) | -16543.29 |  | 2807.09 | -5.89 | <.001 | -22050.8 | -11035.78 |
| PRED | 0.16 | 0 | 1.06 | 0.15 | .881 | -1.92 | 2.24 |
| TMEAN | -159.03 | -0.02 | 177.39 | -0.9 | .37 | -507.08 | 189.02 |
| TWAD | -0.03 | -0.03 | 0.05 | -0.64 | .525 | -0.13 | 0.07 |
| HWAD | -0.07 | -0.03 | 0.11 | -0.69 | .488 | -0.29 | 0.14 |
| AWS | 102.8 | 2.65 | 10.18 | 10.09 | <.001 | 82.82 | 122.79 |
| ROFC | 50.2 | 0.07 | 17.84 | 2.81 | .005 | 15.2 | 85.2 |
| SOMC | 0 | 0.04 | 0 | 1.12 | .265 | 0 | 0.01 |
| PARID | 8225.46 | 0.15 | 2196.28 | 3.75 | <.001 | 3916.37 | 12534.56 |
| OCLEVEL | 98.49 | 0.04 | 132.12 | 0.75 | .456 | -160.73 | 357.71 |
| SOILSCAPE 7 | 5911.34 | 0.57 | 895.39 | 6.6 | <.001 | 4154.58 | 7668.09 |
| SOILSCAPE 8 | -23917.47 | -2.32 | 2108.47 | -11.34 | <.001 | -28054.28 | -19780.65 |
| TILLAGE 0 | -3446.46 | -0.36 | 182.42 | -18.89 | <.001 | -3804.36 | -3088.56 |

## Coefficients

|  | Min | Q1 | Median | Q3 | Max | Mean | SD |
| --- | --- | --- | --- | --- | --- | --- | --- |
| Residual | -5991.82 | -1787.47 | -604.89 | 1507.66 | 16217.44 | 0 | 2802.42 |
| Std. Residual | -2.14 | -0.64 | -0.22 | 0.54 | 5.82 | 0 | 1 |

## Residuals Statistics

This table is a summary of the residuals from a regression analysis. Residuals are the differences between the observed values and the values predicted by the regression model. Let's interpret each component:

###### **Min**

The min value of -5991.82 is the smallest residual in your data. It means the largest underestimation made by your model was by -5991.82 units.

###### **Q1 (First Quartile)**

25% of the residuals are less than -1787.47. These points are underestimations by the model.

###### **Median**

This is the median residual. Half of your residuals are lower than -604.89, and half are higher. A median close to zero suggests that, on the whole, the model is not systematically overestimating or underestimating.

###### **Q3 (Third Quartile)**

75% of the residuals are less than 1507.66. These are overestimations by the model.

###### **Max**

The Max value of 16217.44 is the largest residual. It indicates the largest overestimation by the model was by 16217.44 units.

###### **Mean**

The average of the residuals is 0. If the mean is close to zero in linear regression models it indicates that the model is unbiased on average.

###### **SD (Standard Deviation)**

This value indicates the typical size of the residuals. A smaller standard deviation would indicate that the predictions are generally closer to the actual values.

###### **Standardized Residuals**

Standardized residuals are used to identify outliers in your data. Generally, a standardized residual greater than about 3 or less than about -3 might be considered an outlier. In your data, the range of standardized residuals suggests that there are some outliers (the min is -2.14 and the max is 5.82)

### Assumptions

#### Linearity

To calculate a linear regression, there must be a linear relationship between the dependent and independent variables. In linear regression, a straight line is laid through the data; this only makes sense if there is linearity.


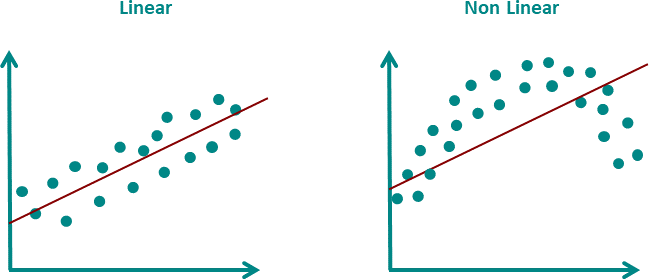


The following graphs result from this model:


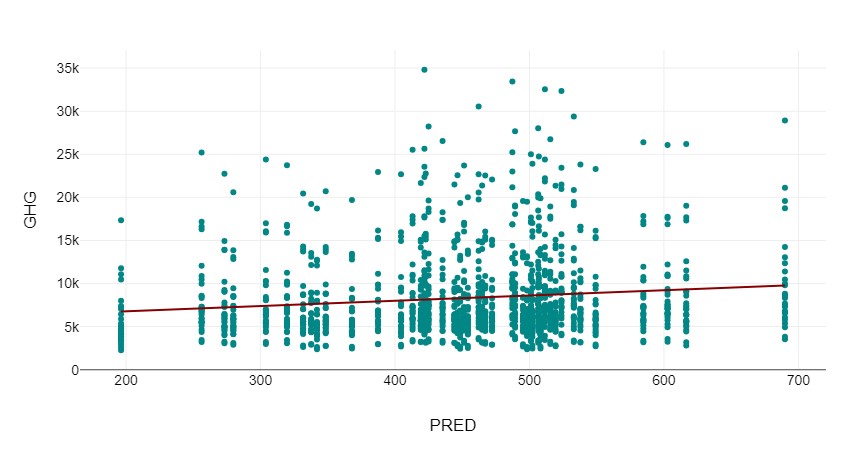


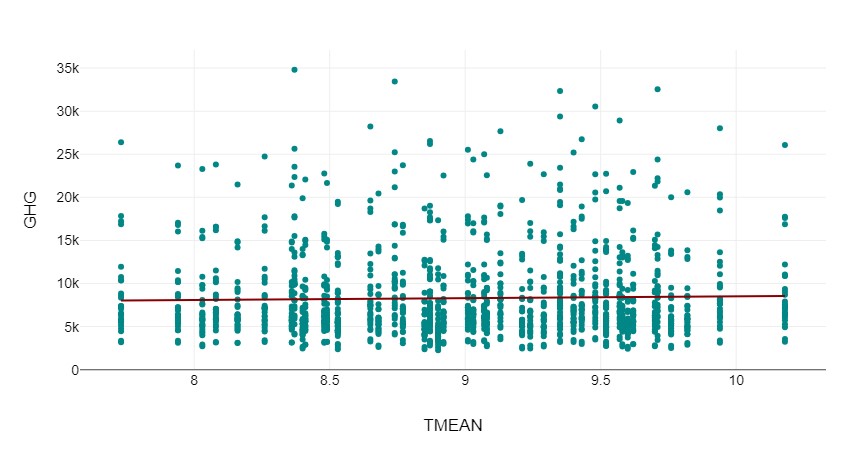


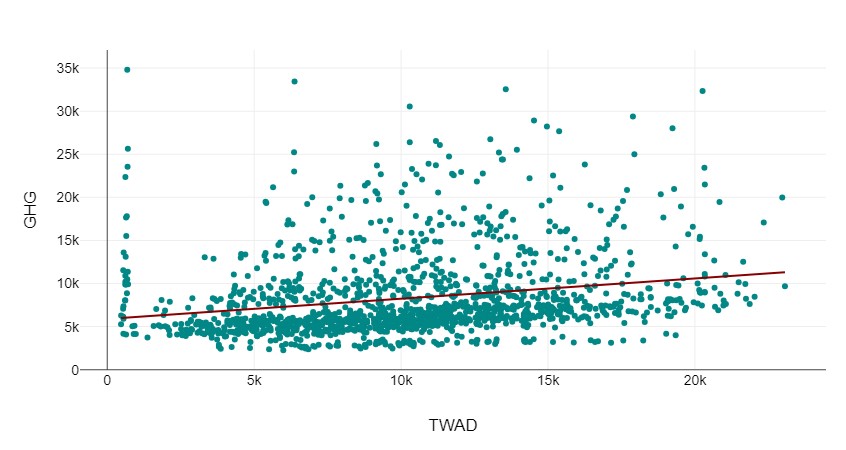


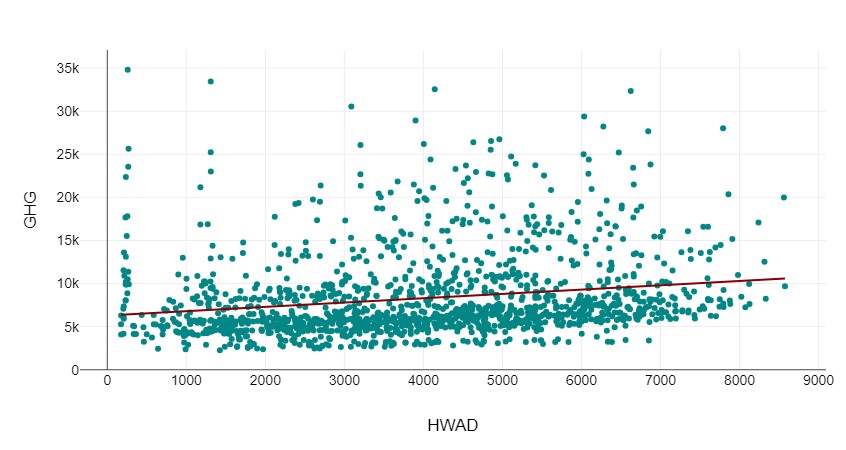


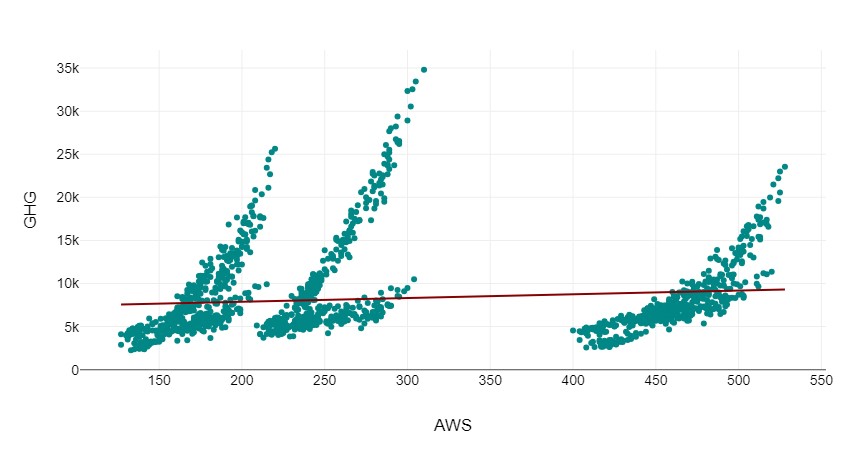


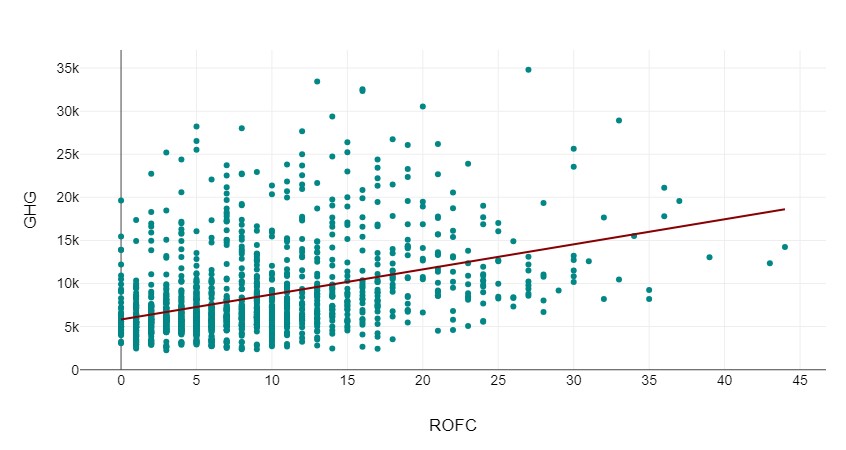


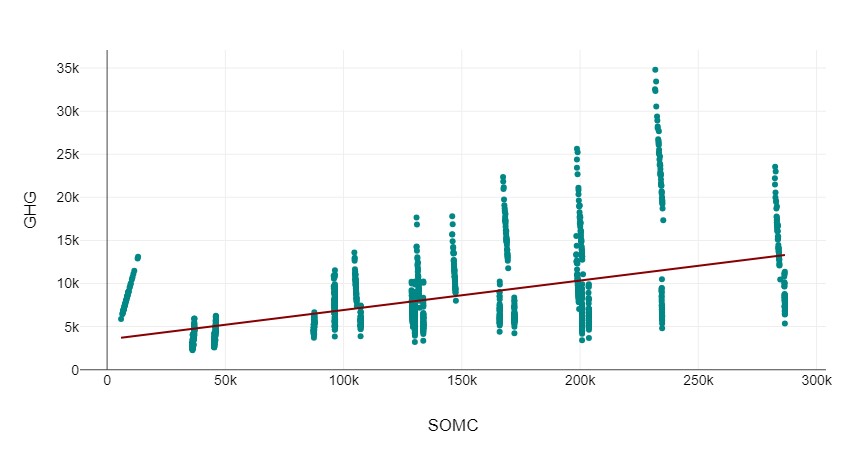


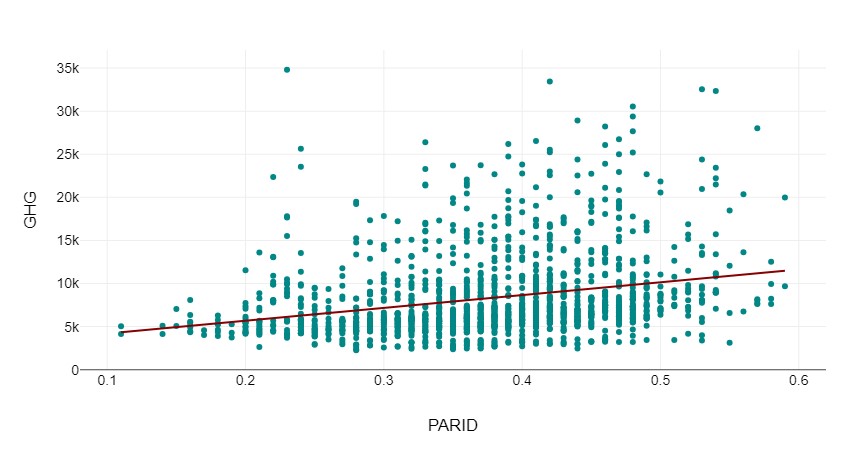


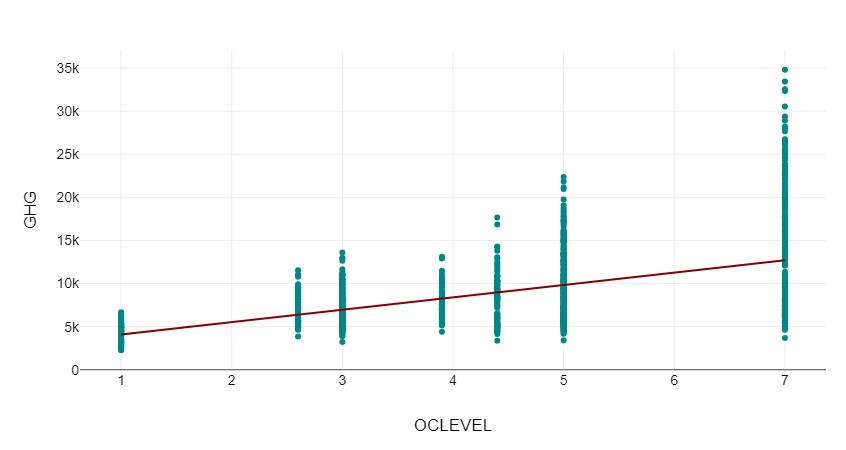


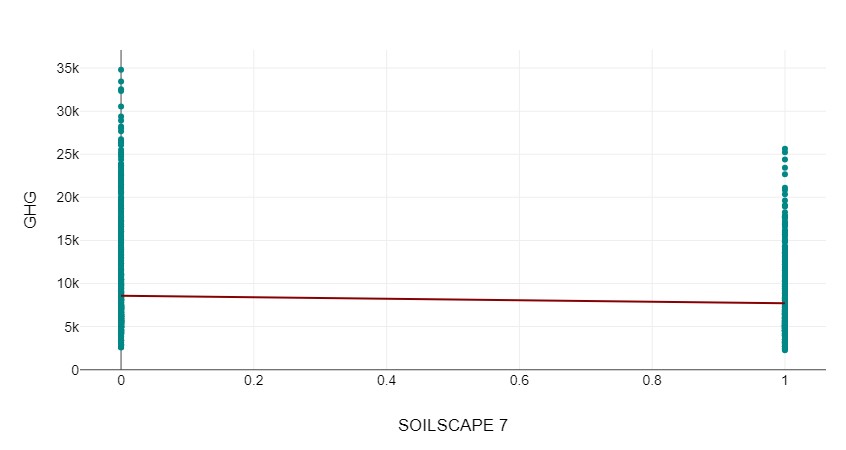


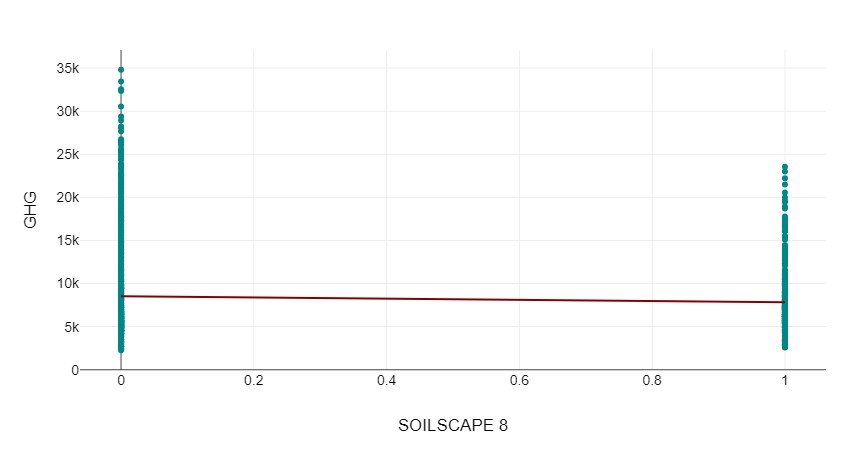


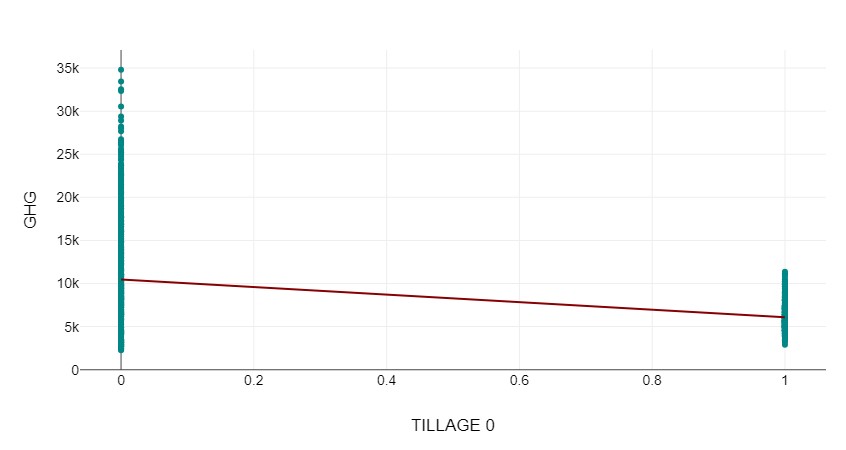


#### Normality of errors

## Tests for normal distribution of Residuum

|  | Statistics | p |
| --- | --- | --- |
| Kolmogorov-Smirnov | 0.1 | <.001 |
| Kolmogorov-Smirnov (Lilliefors Corr.) | 0.1 | <.001 |
| Shapiro-Wilk | 0.93 | <.001 |
| Anderson-Darling | 24.34 | <.001 |


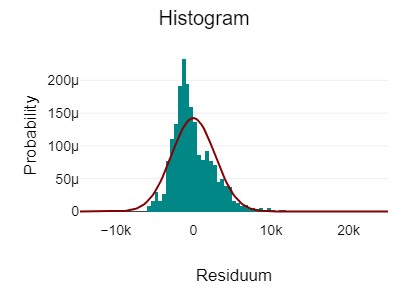


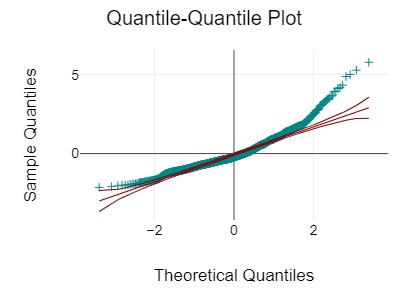


#### Autocorrelation of the Residual

## Durbin-Watson-Test

| Autocorrelation | Statistics | p |
| --- | --- | --- |
| 0.81 | 0.38 | <.001 |

The null hypothesis that the autocorrelation of the residual is zero is tested with a Durbin-Watson test. The calculated p-value of <.001 is smaller than 0.05, so that the null hypothesis is rejected and it can be assumed that there is autocorrelation of the residual.

#### Multicollinearity

Problematic if Tolerance < 0.10 or VIF > 10

| Model | Tolerance | VIF |
| --- | --- | --- |
| PRED | 0.51 | 1.96 |
| TMEAN | 0.52 | 1.93 |
| TWAD | 0.1 | 10.19 |
| HWAD | 0.13 | 7.43 |
| AWS | 0 | 295.55 |
| ROFC | 0.36 | 2.77 |
| SOMC | 0.18 | 5.48 |
| PARID | 0.15 | 6.55 |
| OCLEVEL | 0.08 | 13.13 |
| SOILSCAPE 7 | 0.03 | 32.39 |
| SOILSCAPE 8 | 0.01 | 179.63 |
| TILLAGE 0 | 0.66 | 1.51 |

#### Heteroskedasticity

The variance of the residuals must be constant over the predicted values. Your data must therefore not exhibit heteroskedasticity.


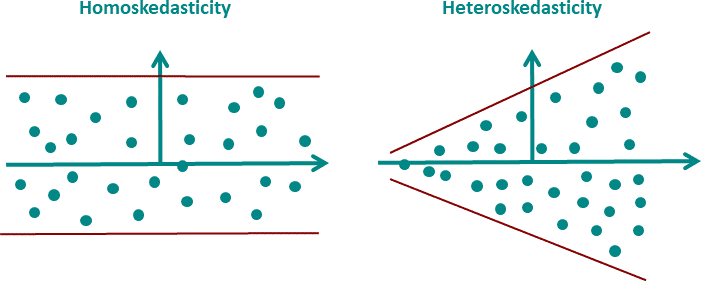


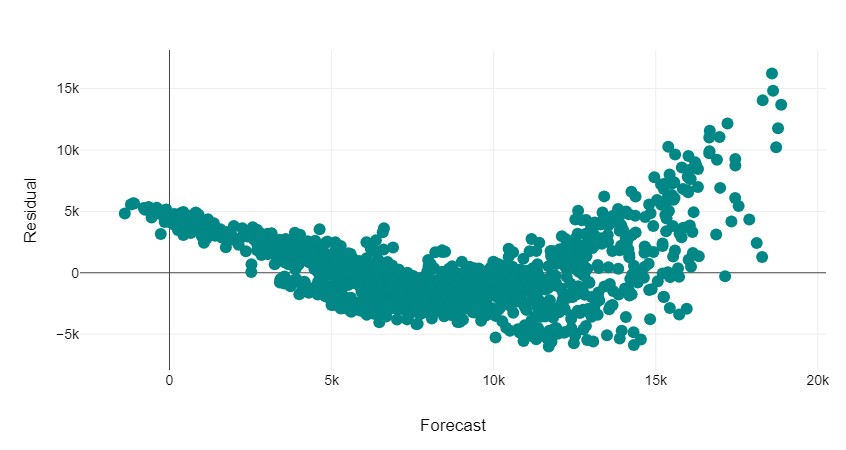
This model results in the following graph

### Effect Size

#### Cohens f^2^

|  | f^2^ |
| --- | --- |
| PRED | 0.19 |
| TMEAN | 0.19 |
| TWAD | 0.19 |
| HWAD | 0.19 |
| AWS | 0.19 |
| ROFC | 0.19 |
| SOMC | 0.19 |
| PARID | 0.19 |
| OCLEVEL | 0.19 |
| SOILSCAPE 7 | 0.19 |
| SOILSCAPE 8 | 0.19 |
| TILLAGE 0 | 0.19 |
